# “Shining a light on chronic pain”: A qualitative study of stakeholder views towards chronic pain at work and the Pain-at-Work Toolkit

**DOI:** 10.1371/journal.pone.0351938

**Published:** 2026-07-02

**Authors:** Angela Claire Thornton, Elaine Wainwright, Wendy J. Chaplin, Victoria Abbott-Fleming, Daniel F. McWilliams, David A. Walsh, Gordon Taylor, Paul McNamee, Karen Walker-Bone, Holly Blake

**Affiliations:** 1 School of Health Sciences, University of Nottingham, Nottingham, United Kingdom; 2 NIHR Nottingham Biomedical Research Centre, Nottingham University Hospitals NHS Trust, Queens Medical Centre, Nottingham, United Kingdom; 3 Aberdeen Centre for Arthritis and Musculoskeletal Health (Epidemiology Group), School of Medicine, Medical Sciences and Nutrition, University of Aberdeen, Aberdeen, United Kingdom; 4 Centre for Pain Research, University of Bath, Bath, United Kingdom; 5 British Pain Society, London, United Kingdom; 6 School of Medicine, University of Nottingham, Nottingham, United Kingdom; 7 Arthritis UK Pain Centre, University of Nottingham, Nottingham, United Kingdom; 8 College of Medicine and Health, University of Exeter, Exeter, United Kingdom; 9 Health Economics Research Unit, Institute of Applied Health Sciences, University of Aberdeen, Aberdeen, United Kingdom; 10 Monash Centre for Occupational and Environmental Health, Monash University, Australia; The University of York, UNITED KINGDOM OF GREAT BRITAIN AND NORTHERN IRELAND

## Abstract

**Purpose:**

The study explored the views of organisational stakeholders who participated in the feasibility trial of the Pain-at-Work Toolkit towards the implementation of the toolkit in ‘real-world’ workplace settings. This digital toolkit was co-created with healthcare professionals, employers, and people with chronic pain and aimed to inform and enable individuals to self-manage their chronic pain at work.

**Design/methodology/approach:**

A qualitative study using semi-structured interviews with 15 stakeholders from 12 organisations that participated in a feasibility trial of the Pain-at-Work Toolkit. Purposive sampling was used to ensure the inclusion of stakeholders with management or supportive roles who have responsibility for the health and wellbeing of employees.

**Findings:**

The findings illuminate three key themes: not all disabilities are visible; not all line managers are equal; and who has control? These surmise that invisible disabilities such as chronic pain are underestimated, poorly understood, and inconsistently provisioned for in organisational policies. It highlights the key role that line managers play in employee disclosure and access to support but demonstrates that line managers vary in their delivery of support to employees. Lastly, it explores stakeholder perceptions of the roles of the employer relative to the employee with chronic pain. It confirms the need for additional resources to plug organisational gaps and give workers tools to self-manage their pain at work.

**Originality:**

This study indicates the need for resources / supports to upskill line managers so they can intervene to proactively support employees with chronic pain to reduce sickness absence and presenteeism (working when unwell). The research demonstrates organisational stakeholders’ interest in a multi-faceted approach to help employees self-manage chronic pain in all types at work, such as that provided by the Pain-at-Work Toolkit. In addition, it clearly identifies the potential for complementary resources to educate and facilitate line managers to better support their staff.

## Introduction

In the United Kingdom, a systematic review and meta-analysis found that chronic pain – defined as pain that lasts or recurs for longer than 3 months [1] – affects 35% to 51% of the population, with a pooled estimate of 43.5% [2]. This equates to around 28 million adults, a figure predicted to increase, largely due to an ageing population. Looking specifically at the working population, the most recently available data, from the 2017 Health Survey for England indicate that 27% of people in employment experience chronic pain [3].

Chronic pain may affect an individual’s ability to work [4–6] which can impact on both the employee and employer. For the individual, any extended absence from work can have a negative impact on their physical and mental health [7–9]. For the employer, productivity losses due to absenteeism [10] and presenteeism [11], as well as impacts on co-workers, can generate a significant financial burden [12,13].

There is increasing recognition of the need to better support people with chronic pain in the workplace. A recent survey, showed almost half of organisations surveyed did not have disability policies; including those designed to support employees with chronic pain [14]. Therefore, strategies that help individuals to self-manage, particularly those which can be applied at work, could offer important potential benefits [15]. Existing options typically focus on a specific approach, for example, exercise [16] or mindfulness [17] or a specific pain such as neck [18] or back pain [19] rather than offering a range of strategies that could be applied to any cause of chronic pain.

Digital interventions are already used to support the self-management of chronic pain [20] and have been shown to be effective in low back pain [21,22] and musculoskeletal conditions [23]. However existing tools digital tools rarely focus specifically on workplace self-management strategies and where work-related content is included it is typically limited and lacks in-depth resources. The Pain-at-Work Toolkit was developed to address this identified gap by providing a digital resource specifically designed to support workplace self-management of chronic pain [24]. This digital toolkit was co-created with healthcare professionals, employers and people with chronic pain aiming to provide a comprehensive resource to enable the individual to better self-manage their pain at work, regardless of its cause.

In this paper, we report findings from a study with organisational stakeholders, selected because they play a key role in workforce health and wellbeing. Selected organisations had participated in a feasibility trial of the Pain-at-Work Toolkit.

## Methods

### Study design

This was a qualitative interview study, with individual semi-structured interviews. Reporting aligns with the consolidated criteria for reporting qualitative research (COREQ [25]) (S1 Table). Since the aim was to explore the individual stakeholder’s views rather than gain a global perspective [26] semi-structured interviews were conducted. This approach allowed the discussion to be focused on key topics while also allowing the researcher the flexibility and autonomy to explore relevant topics if and when they were raised [27].

### Recruitment and Interview Schedule

The interview topic guides for both the intervention and control groups were developed by the project team (HB, WJC, AT, EW) (S3 Text, S4 Text). A project researcher (AT) approached named organisational representatives from the Pain-at-Work feasibility trial via email in March 2025, at the end of their participation in the trial (6 months after baseline). If no initial response was received, up to two additional follow-up emails were sent in March and April 2025. At recruitment, potential stakeholders were informed about the study’s objectives, format, and available interview dates. They were assured that entry into the study was entirely voluntary and that their employment would not be affected by their decision. Fieldwork was conducted between April 1, 2025, and May 27, 2025. Interviews were conducted at a mutually convenient time using videoconferencing hosted by Teams (Microsoft Corporation) and with one researcher (AT) who also took field notes. Written informed consent and demographic data were obtained via Smart Survey accessed through an email link with additional verbal consent audio-recorded prior to the interview. All but two of the interviews were audio and video recorded. Detailed notes were taken for the two stakeholders who did not consent for interviews to be recorded. Study forms were held securely in locked cabinets and access was limited to authorised personnel. All data were held on secure password protected servers with restricted access. Full interview transcripts were automatically generated in Microsoft Stream. Each transcript was checked by AT to ensure the interview content was accurately represented.

### Stakeholders

The target population was individuals (stakeholders) employed in a role providing management or support for employees in an eligible organisation participating in the feasibility trial of the Pain-at-Work Toolkit [24]. Eligible organisations were defined as organisations located in England, from any sector (public, private or third) with 10 or more employees. Organisations we classed as small if they had 10–49 workers, medium-sized if 50–249, or large with 250 or above [28]. A total of 30 individuals from the 18 participating organisations were invited to take part in an interview. These individuals played a key role in supporting employee health and wellbeing at the participating worksites. Interviews were conducted with organisational stakeholders (n = 15), representing 12 organisations. Analysis was conducted at both the individual (stakeholder) level and, where appropriate, at the organisational level, to reflect shared or organisation-specific perspectives. Only stakeholders from organisations allocated to the intervention group (n = 7 organisations; n = 8 stakeholders) were asked questions relating to the Pain-at-Work Toolkit (see RQ3 below). The Pain-at-Work Toolkit is a digital resource designed to support employees in the self-management of chronic pain in the workplace. It comprises five sections covering understanding chronic pain, chronic pain and disability, information on workplace adjustments and support, self-management strategies and links to additional resources. The toolkit was made available to employees within organisations randomised to the intervention arm of the feasibility trial.

### Ethics

The University of Nottingham Faculty of Medicine and Health Sciences Research Ethics Committee granted ethics approval on March 31, 2023 (FMHS 237–0323). Minor amendments were noted to file on 12.04.2023, 27.06.2023, 14.11.2023, 25.10.2024, 19.03.2025, 08.04.2025, and 04.08.2025. Our study protocol was reviewed and approved by HRA and Health and Care Research Wales (HCRW) on July 4, 2024 (IRAS 336655). The trial was prospectively registered on May 1, 2023 (ClinicalTrials.gov NCT05838677).

## Research questions

The research questions (RQs) were:

RQ1: What is the organisational culture around supporting people with chronic conditions at work?

RQ2: What policies and practices do organisations have in place to support people who have chronic conditions (and specifically chronic pain) at work?

RQ3: How do organisations perceive the Pain-at-Work Toolkit as a mechanism for supporting people with chronic pain at work?

Stakeholders were also asked to estimate the number of employees in the organisation and the percentage of workers with chronic pain in their organisation at recruitment into the trial and then at interview. These data were collected to provide contextual insight into organisational stakeholders’ perceptions of the prevalence of chronic pain within their workforce.

## Analysis

Inductive data analysis following the principles of reflexive thematic analysis by Braun and Clarke was deployed [29]. This thematic analysis has six stages but the approach to analysis is organic and flexible whereby the researcher moves back and forward between the six phases rather than using a fixed, sequential approach [29–31]. The first step involved familiarisation with the data. The lead researcher (AT) who conducted the interviews with stakeholders, and checked the transcripts, undertook this process to ensure a deep and sustained engagement with the dataset. A data driven or inductive approach was used to generate initial codes which offered ‘answers’ to the key research questions. Codes were then organised into preliminary themes which reflected patterns of shared meaning. Themes were iteratively reviewed, refined, and clearly defined to ensure internal coherence and distinction between themes, with each theme anchored by a central organising concept [32]. This process was led by AT and informed through ongoing discussion with other members of the research team (HB and EW), who contributed to the refinement and interpretation of themes through critical reflection. To support rigour and transparency, the analysis process was verified against Braun and Clarke’s 15-point checklist [33] (See Supplementary material S2 Table). Verbatim quotations used in reporting were lightly edited by AT to correct spelling and grammar generated by auto transcription while preserving the original meaning.

Reflexive thematic analysis places the researcher at the heart of the process [29]. The study was informed by an interest and lived experience of how chronic pain is experienced and managed in the workplace and how stakeholder perceive they and the organisation they work for support employees. HB is a behavioural scientist and health psychologist with 30 years of experience in public health and applied health research. WJC and EW have a background in health psychology and a particular research interest in work and health, including chronic pain and work. AT has a background in psychology and over 35 years’ experience as an industry researcher specialising in healthcare.

## Information Power

The sufficiency of the sample of 15 was assessed using the information power model where interview quality is more important than quantity [34]. In this model, Malteraud et. al., specify five interconnected factors which impact on information power in qualitative studies. These factors comprise 1) study aim, 2) sample specificity, 3) extent of established theory, 4) quality of dialogue between participant and researcher and 5) analysis strategy. Participants were purposively sampled from organisations participating in the Pain-at-Work feasibility trial with the aim of recruiting stakeholders with relevant roles in supporting employee health and wellbeing. A total of 30 stakeholders were invited to participate of whom 15 were recruited. Recruitment continued until the available pool of eligible participants had all been approached and responses maximised. The study aim was focused, and the sample was specific, comprising organisational stakeholders with direct experience relevant to the research questions., There is also an existing body of literature on chronic pain in the workplace to support interpretation of the findings. The quality of the dialogue was considered to be high reflecting the experience of the interviewer and the richness of the data [35]. The analysis strategy involved identifying shared patterns to generate themes relevant to real world organisational policies and practice. During analysis, the research team determined that the data provided sufficient depth and richness of insight to address the study aims and that no substantially new themes were identified. On this basis the sample was judged to have sufficient information power.

## Results

Depending on the focus of analysis, findings are reported at either the individual (stakeholder, n = 15) or organisational level (n = 12 organisations represented). The final section summarising stakeholders’ responses relating to the Pain-at-Work Toolkit includes only the views of stakeholders from organisations that were allocated to the intervention group in the trial (n = seven organisations, eight stakeholders) since employees in the control group did not receive access to the toolkit. The data source is stated throughout.

### Organisational Characteristics

Twelve organisations from the Pain-at-Work feasibility trial were included, from which stakeholders for this qualitative study were recruited., Eleven organisations were classified as large, i.e., with >250 employees [28]. At the time of recruitment, seven organisations had been randomised to the intervention arm of the feasibility trial (with access to the Pain-at-Work Toolkit) and five to the control arm. The organisational stakeholders sample included representation from both public and private sector organisations. They were almost equally represented in the control group, whereas the intervention group were predominantly public sector.

A summary of organisational characteristics is shown in Table 1.

**Table 1:** Organisational characteristics (n = 12).

Characteristic	Summary
Sector	Public sector n = 8 Private sector n = 2 Third sector n = 2
Industry	Public administration/local government/ civil service n = 3 Healthcare n = 6 Professional services n = 1 Arts n = 1 Utilities n = 1
Organisation size	Large organisations (>250 employees) n = 11

### Stakeholder Characteristics

Fifteen stakeholders were interviewed for this study: eight individuals from seven organisations in the intervention arm of the feasibility trial of the Pain-at-Work Toolkit and seven stakeholders from five organisations in the control group. Interviews lasted between 30 and 58 minutes with a mean of 44 minutes. Stakeholders’ time in their current role ranged from a few months to 14 years, with an average of just over 5 years. Overall, the roles of stakeholders focused on wellbeing and occupational health, contributing to or leading strategy and policy around employee wellbeing. Duties included conducting employee assessments, giving advice about health and wellbeing and signposting, and/or supporting the employee to access other specialist services. These services included occupational health, physiotherapy, and a community pain clinic. Approximately one third of stakeholders reported current or previous line management responsibilities and drew on their experience during the interviews. A summary of stakeholder roles is shown in Table 2.

**Table 2:** Stakeholder roles (n = 15).

Category	Roles represented
Senior leadership / strategic roles	Director Head of Workforce Policy and Wellbeing Head of Occupational Health & Wellbeing Head of Wellbeing People Business Partner
Line management experience (current or prior)	~6
Occupational health and wellbeing including clinical roles	Occupational Health Advisor Senior Occupational Health Advisor Occupational Health & Wellbeing Wellbeing Lead & Occupational Health Technician Occupational Therapist Musculoskeletal (MSK) & Occupational Health (OH) Physiotherapist Lead Research Nurse Service Lead Senior Clinical Research Practitioner Practice Educator

Participant demographics are summarised in Table 3.

**Table 3:** Stakeholder demographics (n = 15).

Characteristic	Category	n (%)
Age (years) • Range 30–62 years • Mean 48.6 years		
Gender identity	Female Male	11 (73%) 4 (27%)
Ethnicity	White Indian	13 (87%) 2 (13%)

Information about the organisational context was collected at two time points: first at recruitment into the feasibility trial, and subsequently at the stakeholder interview. In most cases, the organisational representative (“champion”) who provided consent for participation in the feasibility trial differed from the stakeholder interviewed in this study.

### Thematic Analysis

**Fig 1:**
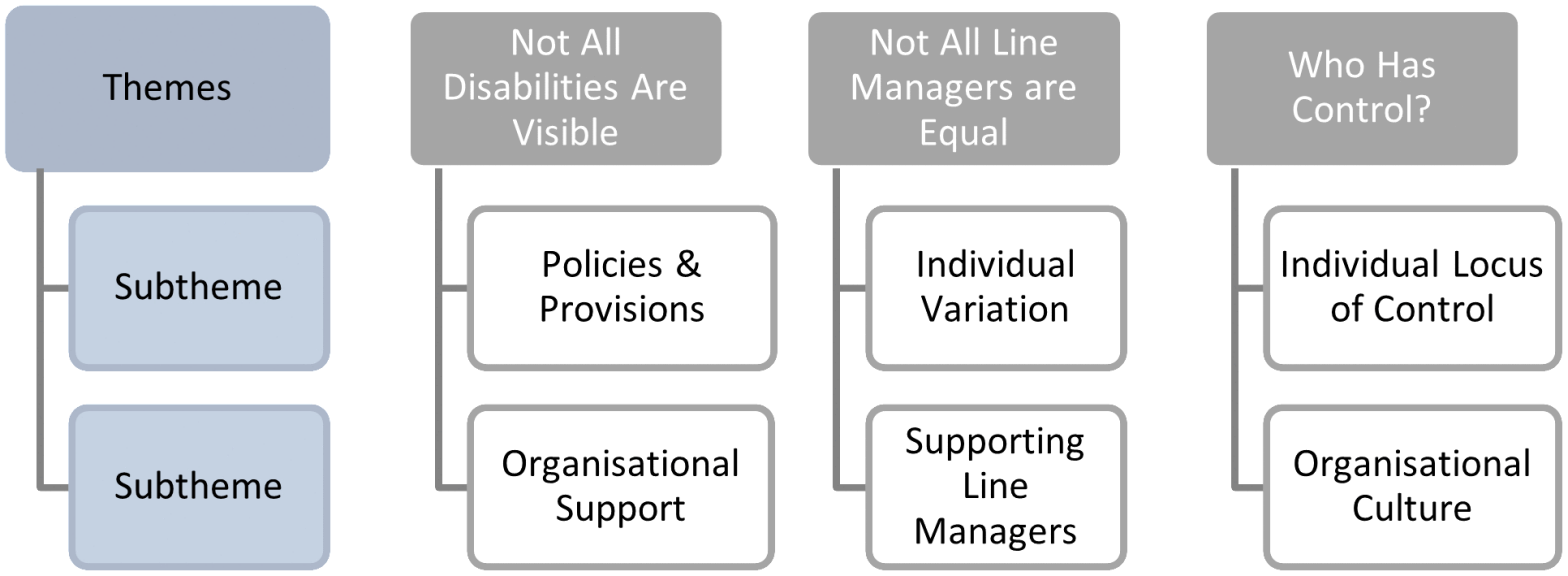
Themes.

As shown in Fig 1, three main themes (each with two subthemes) were identified from stakeholders’ views of their organisational policies and procedures for employees with chronic pain. Each theme and subtheme, where appropriate, is discussed in turn and illustrated by anonymised quotations with participant identifier and role. Participant IDs refer to individual stakeholders and are used to anonymise quotations; the views expressed reflect their perspectives based on their professional role within their organisation, rather than representing formal organisational positions.

#### Theme 1: Not All Disabilities Are Visible.

This theme captures the challenges associated with recognising and responding to chronic pain as an often invisible and unpredictable condition within the workplace. Stakeholders highlighted the difficulty in identifying employees in need of support and the implications this has for organisational responses.

This theme comprised two subthemes:

a) Policies and Provisions refer to the formal organisational structures, policies, and resources in place to support employees with chronic pain, including occupational health provision and reasonable adjustments.
b) Organisational Support captures the extent to which support is perceived to occur in practice, including managerial responsiveness.

a) Policies and Provisions

This subtheme explored the extent of organisational policies and provisions for chronic conditions, including pain, and how visible and accessible these are for employees. It also covers stakeholders’ perceptions of how supportive the organisational culture is, as well as any gaps in provision. This theme also addresses stakeholders’ motivations for participating in the Pain-at-Work Toolkit feasibility trial and stakeholder interviews.

By participating in the feasibility trial, organisations hoped to send a clear message to all staff that chronic pain should be taken seriously and, ideally, accommodations be put in place before it resulted in long-term sickness absence, thus benefitting both the individual employee and the employer. There was a willingness to learn from the Pain-at-Work feasibility trial and the resources available within the Pain-at-Work Toolkit and to use the results to inform evidence-based practice. Although this was only overtly stated by a couple of stakeholders, another potential contributing factor was that by being involved in the trial, organisations would improve their corporate image and metrics.

*“Saying that to everybody across the organisation, whether you’re an employee thinking about do I need to have a conversation about this or how am going to approach this conversation… but also if you’re thinking about managers, if somebody is talking to you and having that conversation saying that the organisation treats that as something that’s important and needs support”.* [ID15, Occupational Health & Wellbeing]

Several organisational policies and provisions were discussed within the subtheme *Policies and Provisions* (see Table 4). These represent examples reported by stakeholders rather than additional analytic categories.

**Table 4:** Policies and Provisions.

Policy	Number of Organisations Providing (n = 12):
Reasonable Adjustments	12
Sickness Absence	12
Support Services:	
• Occupational Health	10 (majority – internal)
• Health and Wellbeing	8
Physiotherapy	8
• Employee Networks	6
• Wellbeing/Work Adjustment Passport	4
• Employee Assistance Programme (EAP)	3

Since organisational policies were self-reported rather than systematically audited, the list shown below (ordered by frequency of mention), is not necessarily exhaustive. The terminology below reflects stakeholder language.

i) Specific Policies for Chronic Pain

Although 11 of the 12 organisations were classified as large, none of the organisations surveyed had specific policies to support employees with chronic pain. Broader policies were mentioned, which aimed to support those with other long-term conditions or disabilities. Consequently, chronic pain was not clearly referred to in organisational policy or specifically provided for. Chronic pain was widely perceived as a “hidden” or “invisible” condition, both because employees may not disclose their experiences and because organisational support is often contingent on such disclosure, with implications for the support that employees receive. This interpretation was further supported by the finding that seven of the 15 stakeholders had no idea of the proportion of employees with chronic pain, and the remaining eight could at best guess at anything from occasional cases to a high proportion of the workforce.

*“I don’t think there’s anything specific just for pain. I think it is wrapped up with everything else. It does come under sort of long-term conditions.”* [ID07, Head of Wellbeing]

*“In terms of policies and practices, it depends on what you mean by where they sit. I don’t think we still have anything that explicitly talks about it.”* [ID15, Occupational Health & Wellbeing]

Stakeholders acknowledged that invisible disabilities, such as chronic pain, (often compared to mental health conditions), were poorly understood and could be stigmatised for example through scepticism about the legitimacy of chronic pain and concerns about being judged or not taken seriously by employers, managers, or colleagues and received less support than visible disabilities like physical or sensory impairments. This reflects an awareness of the challenges associated with invisible conditions, rather than a detailed understanding of the prevalence of chronic pain and its impact within the workplace.

*“Stigma around some people with conditions like fibro(myalgia) is still quite apparent, and that’s even within healthcare, never mind a manager or an HR professional.”* [ID11, MSK & OH Physiotherapist]

This quote illustrates both an awareness of the challenges associated with invisible conditions and the persistence of scepticism and misunderstanding surrounding chronic pain.

*“If you can’t see it, it isn’t a broken leg. You haven’t got a bad back; you know that sort of thing, or you’re not in a wheelchair…I really don’t understand what the issue here is. And it’s a bit like mental health. You know the two go hand in hand.”* [ID07, Head of Wellbeing]

ii) Reasonable Adjustments

As required by the Equality Act 2010 [36], all 12 organisations made reasonable adjustments typically in conjunction with a personalised enhanced Display Screen Equipment (DSE) assessment often undertaken by the Health and Safety Team. Stakeholders were generally familiar with typical adjustments required for some types of chronic pain, mainly musculoskeletal. These included customised workstations such as ergonomic chairs, standing desks and adjustable footrests, and a few stakeholders specifically referenced the UK Government’s Access to Work scheme as a means of providing additional resources.

Stakeholders often described reasonable adjustments in relation to specific functional limitations rather than chronic pain as a condition, for example:

*“So, if it was about if it was about chronic pain it would depend on what kind of problem at work that was. But if it was about difficulty sitting for long periods of time then you might be directed to have an enhanced DSE assessment with the health and safety team.”* [ID01, Head of Workforce Policy and Wellbeing]

*“So, we do the DSE assessments, you know, so that’s about your screen, your keyboard, your mouse, your chair, things like this.”* [ID12, Lead Research Nurse]

Adapted working patterns for employees with chronic pain, such as frequent breaks, flexible working hours, and/or hybrid working arrangements, were also provided. These arrangements were typically implemented as part of reasonable adjustments in response to specific functional limitations associated with chronic pain. However, stakeholders noted that accommodating such adjustments could be challenging in practice, as explored further in relation to the role of line managers (Theme 2: Not All Line Managers Are Equal). However, some stakeholders noted that it could be challenging to accommodate these changes while still meeting operational demands. This, together with other comments about limited resources and high workloads in support services, such as occupational health, highlights the real-world challenges of accommodating an employee with chronic pain.

*“A typical adjustment, say, for someone with an arthritic condition…whether that’s in winter, cold conditions, or humid conditions, or if their pain is worse in the mornings is that they they’re allowed to start later, or if they have the ability to do that, to be able to work from home to avoid having to do that commute”.* [ID14, Occupational Therapist]

*“So, if I give you an example of the of a tailored work plan for somebody with back pain. So, what we might do is we introduce rest breaks to move around, which is sort of 5 minutes or 10 minutes in between each patient. We might reduce the amount of driving so they can do a mixture of hybrid working… And we alter their working rota so that they’re still seeing the same amount of patients or still having the same amount of patient contacts, but it’s spread out a bit more. The difficulty is you still got patients to see as well.”* [ID10, Service Lead]

Some stakeholders recognised the need for individualised adjustments rather than a “blanket” solution but acknowledged this was not always reflected in policy and practice. One individual who had lived experience of chronic pain had themselves experienced a “one size fits all” approach at work: *“Myself and several colleagues all have lower back pain after childbirth. We are all very different but we all got a blanket set of advice.”* [ID08, Senior Clinical Research Practitioner]

*“But, you know, reasonable adjustments are, gosh, they’re just so varied, aren’t they? It’s about how long can you sit? How does that affect you? … this is why I say it’s a really individual basis because you can’t put a blanket set of questions on one person. And if you do, all you do is make it a process.”* [ID12, Lead Research Nurse]

Two stakeholders, each representing a different organisation (both third sector), demonstrated a particular willingness to provide a wide range of equipment in offices to help with disabilities, including walking pads, TENS machines and heated pads and blankets. In one case, this appeared to be influenced by the values and lived experience of a senior leader. The other was working with estates and facilities to embed wellbeing into office design so that a range of needs were accommodated. This included considering how carpets impacted wheelchair users and chairs with arms for individuals who needed support to stand up, as well as chairs without arms for those who use walkers or sticks. These actions were the result of a proactive Occupational Health and Wellbeing Lead who had implemented learnings from a previous role. However, among stakeholders, this was the exception rather than the norm, with most organisations relying on standardised solutions.

iii) Sickness Absence

All of the 12 organisations had what is officially termed a ‘sickness absence policy’ although this exact terminology was used by less than half of the 15 stakeholders; the most common language being ‘sickness’ or ‘sick’. The widespread use of these terms may reflect unconscious attitudes—for example, framing sickness as the individual employee’s problem rather than something the organisation should support – and some stakeholders indicated that individuals were expected to take responsibility for their own health. A few stakeholders spontaneously referenced chronic conditions (although not necessarily chronic pain) noting that policies outlined expectations for both employees and line managers, particularly in managing extended or frequent absence. However, stakeholders acknowledged that this process was far from perfect since support was typically only offered when sickness absence had become an issue for an employee’s line manager rather than aiming for early intervention (e.g., a reactive rather than proactive approach). As one stakeholder noted, “*It shouldn’t just be when people have been off sick… how can we be having those open conversations in advance of that.*” [ID15, Occupational Health & Wellbeing]

This was further reflected in accounts suggesting that support is often only initiated once problems escalate: “*they have tried to self-manage for a while… it’s normally when they feel that there is a tipping point… and then the manager refers in*.” [ID09, Senior Occupational Health Advisor]

In contrast, examples of more supportive approaches were limited. One of the 12 organisations (third sector) also offered a ‘disability leave policy’ which, in addition to annual leave, allowed staff up to 5 paid days a year to attend medical appointments. This particular organisation was one of two that provided a particularly wide range of accommodations for workers with disabilities.

Analysis of stakeholder accounts indicated that chronic pain was sometimes associated with long-term health conditions, including long COVID, fibromyalgia and musculoskeletal conditions such as lower back pain and rheumatoid arthritis. However, while some chronic conditions could be recorded within organisational systems, sickness absence reports were not structured in such a way that employees with chronic pain could be easily identified. As a result, accurate data relied on individual disclosure which was influenced by both organisational culture and, as discussed subsequently, line manager practices (Theme 2: Not All Line Managers Are Equal).

*“We don’t have those figures and it’s in part because of the way we record the statistic, because quite often with chronic pain, it’s going to be something that comes up in conversation as a secondary order even if it actually turns out to be primary. I haven’t found it really is something that people lead with, and so it won’t be recorded necessarily either in their occupational health referral or with their sickness absence.”* [ID15, Occupational Health & Wellbeing]

*“So, we do have arthritis codified. We do have, you know, obviously injuries that occur in the workplace and I’ll just scan down here to see what we’ve got. Chronic fatigue and fibromyalgia, connective tissue disorder. Let’s have a look to see if we’ve got any chronic pain. We have muscles, skeletal disorder, broken down... fracture injury, lower and upper limb. Capture whether somebody’s had surgery for orthopaedic reasons. So, it’s not even close, we do have simply neurological. So yeah, so the answer is we don’t capture, don’t codify chronic or persistent pain.”* [ID09, Senior Occupational Health Advisor]

iv) Occupational Health

Ten of the 12 organisations provided occupational health services, the majority of which were provided internally but this may reflect the predomination of larger companies in the sample. Occupational Health was accessed mainly by referral from line managers and hence an individual’s line manager had considerable influence on employees’ access to support. Only two organisations allowed an employee to self-refer to occupational health; in the majority of cases this route was not available. Interestingly, one organisation had changed the process so that human resources rather than line managers could make staff referrals: the rationale being greater consistency and expertise; a theme which is explored in more detail later when we consider the impact of line managers on employee support at work (Theme 2: Not All Line Managers Are Equal).

*“I know in lots of organisations, then it might be the line manager that can do a direct referral to occupational health. As we’re trying to get this consistency of support and as we’re trying to support managers in how to approach this, we don’t actually do that at xxx at the moment that got changed a couple of years ago. So, you would talk to my HR colleagues and they would do that referral so they would be able to mediate and support and help.”* [ID15, Occupational Health & Wellbeing]

Several stakeholders in occupational health felt that a lack of staff and restrictions on the amount of time they were able to spend with employees limited the support they could provide. While all but two of the 12 organisations offered occupational health services, there was noticeable variation in their capacity. Examples of the ratio of occupational health staff to other workers ranged from one occupational health worker for approximately 3,500–5,700 employees to one occupational health worker for every 250 staff.

*“I think there is something around expanding the remit of occupational health. So, you know, having more staff to enable us to be able to see people once, see people again a month later and then maybe meet with the manager as well to provide more ongoing follow-up, I don’t think a one-off consultation report cuts it for people who have been living with chronic pain for potentially years”.* [ID14, Occupational Therapist]

v) Physiotherapy

Eight of the 12 organisations also mentioned employees having access to physiotherapy, which in a few cases was subcontracted to an external organisation, and regardless of provider, was most often accessed by a referral from occupational health. Relatively little was discussed about the role physiotherapy could play in chronic pain although individuals noted that employees needed to be motivated to do the exercises recommended by the physiotherapist and that physiotherapy needed to be supported by changes in the working environment typically made under the umbrella of reasonable adjustments.

*“When it comes to sort of managing pain, usually there is quite a lot of input required, particularly with the main sort of processes that we push, say things like physiotherapy.”* [ID03, Head of Occupational Health & Wellbeing]

*“Staff have access to physios via self-referral or referral by Occupational Health. They are an external provider, but we closely manage the process, including holding records on site.”* [ID06, Occupational Health Advisor]

vi) Employee Assistance Programme (EAP)

Three of the 12 organisations offered an Employee Assistance Programme (EAP) which provided independent resources and services such as counselling and advice on mental or physical health issues. Chronic pain was not mentioned specifically, so the ability of the programme to provide tailored advice is unknown.

*“People can call that, if they want to talk to someone about a medical concern they’ve got. That’s staffed by a nursing team so they can get some support there.”* [ID01, Head of Workforce Policy and Wellbeing]

*“We do have an online Employee Assistance Programme which offers employees the opportunity to speak to somebody independent of the organisation for a range of advice topics, whether that be health-related or psychosocial issues.”* [ID09, Senior Occupational Health Advisor]

vii) Health and Wellbeing

Eight of the 12 organisations offered Health and Wellbeing, although the scope and form of this varied. Examples included regular events, such as presentations and webinars, from both internal staff and external experts, covering a range of topics. Wellbeing advice and signposts to resources were also typically available on the organisation’s intranet, and individuals mentioned specific campaigns, for example, aligned to national ‘awareness’ days or weeks. Arthritis UK (a UK charity providing support for people with arthritis) and ‘Flippin’ Pain’ (a UK-based public health campaign focused on changing how people understand, talk about, and treat pain) were specifically mentioned by a couple of stakeholders as valuable resources for employees with chronic pain. Some stakeholders referred to staff who were designated as wellbeing champions or advocates and whose role was to offer advice and support to colleagues. However, mental health conditions appeared to be given a higher profile, with musculoskeletal (not specifically chronic pain) only being mentioned twice spontaneously.

viii) Employee Networks

Six of the 12 organisations had employee-led networks as a means of providing a safe space for staff to discuss, share experiences, and offer support to one another. These spanned a wide range of groups, typically under the banner of Equality, Diversity, and Inclusion (EDI), including gender, sex, and sexual orientation, parental leave, working practices, and disability. Chronic pain was most likely to be covered in networks for people with disabilities and/or long-term health conditions rather than a specific forum.

*“We tried to listen to what, you know what our demographic wants and needs, and we tried to make sure that they have a safe space within the organisation that they feel represented in.”* [ID04, People Business Partner]

*“So, for example, we have one which is titled xxx which is designed for employees with some form of underlying health issue or long-term health condition or you know those who find themselves subject to the need for workplace adjustments. It’s a peer-led work group.”* [ID09, Senior Occupational Health Advisor]

ix) Wellbeing/Work Adjustment Passport

Four of the 12 organisations offered what was either described as a ‘Wellbeing or Work Adjustment Passport,’ which seems comparable to a Health Adjustment Passport (Department of Work and Pensions). The work document was primarily described as a means of providing a record of the employee’s needs and adjustments should they change roles and/or managers. In one instance, it was a response to numerous queries from employees with long-term conditions, including chronic pain. In a couple of cases, the document was also seen as a way of structuring a conversation between the employee and their line manager about health and wellbeing, for example, by exploring how the condition affected them, what triggered it, what the individual could do to support themselves, what they needed from their manager and the organisation and examples of what was possible. Although atypical, this extension of use may have merit, particularly given the important role that line managers play in supporting employees at work (Theme 2: Not All Line Managers Are Equal).

x) Human Resources

Four of the 12 organisations mentioned Human Resources, although it seemed that some aspects of the role were shared with the Health and/or Wellbeing team.

*“So, for example, every month we will have a wellbeing conversation where every colleague’s invited to join, they are all recorded if you can’t join and we invite expert speakers in to speak, to talk to colleagues and that will be a range of topics, it will change every month. But sometimes we do have things that would support things around pain, you know, so physical wellbeing.”* [ID01, Head of Workforce Policy and Wellbeing]

*“We have a UK Health and Wellbeing Lead and a deputy lead to look after that mandate within the organisation. They run sort of supportive webinars for staff as well as sort of oversee or coordinate the various staff networks.”* [ID09, Senior Occupational Health Advisor]

Overall, while a range of policies and provisions were reported, stakeholders consistently highlighted a lack of specific recognition and tailored support for chronic pain within organisations.

b) Organisational Support

Stakeholders’ comments indicated inconsistent support for employees with chronic pain, both across and within organisations, which was potentially underpinned by a limited understanding of the nature of chronic pain and its possible impact on an employee’s working life. Six of the 12 organisations were said to be supportive of employees with chronic conditions generally. However, in the remaining half, stakeholders identified both strengths and weaknesses in the organisational culture. One key weakness was said to be inconsistent and inadequate support which stakeholders attributed to two main factors. Firstly, health was often seen as a binary construct by employers, where individuals were either well enough to work or needed to be off sick, rather than a scenario where an employee could be supported to stay in work. Secondly, the unseen, unpredictable symptoms of conditions with chronic pain, such as fibromyalgia, were poorly understood and often stigmatised.

*“I definitely think physical impairment is probably more recognised and accepted than mental health and chronic pain because people just don’t understand… If you can’t see it, it isn’t a broken leg. You haven’t got a bad back; you know that sort of thing or you’re not in a wheelchair.”* [ID07, Head of Wellbeing]

*“I would say it is really similar sense to - we do a lot of work in terms of kind of mental health - and I kind of see a similarity there in that if something’s not visually obvious, sometimes it can be really quickly dismissed.”* [ID03, Head of Occupational Health & Wellbeing]

*“And I think there’s a very long time there’s been a distinction, and especially this sort of mentality that we’re challenging all the time in our managers of whether I’m fit enough to be at work or they’re not.”* [ID01, Head of Workforce Policy and Wellbeing]

According to two representatives from the same organisation, one private sector company was very poor at supporting employees. This was attributed to the demographic of the workforce (typically older men) who were reluctant to disclose health issues. However, the culture was seen to be slowly improving, as evidenced by more employees seeking occupational health advice and support.

*“I think because you’ve got a culture of requirement to meet deadlines coupled with the culture of people not necessarily wanting to ask for help or a bit scared to kind of raise concerns.”* [ID03, Head of Occupational Health & Wellbeing]

Stakeholders typically felt that organisations needed to improve their support of employees with chronic pain. Currently, supportive intervention was said to occur when an employee’s attendance becomes an issue, but stakeholders wanted to see adjustments put in place earlier and, ideally, introduce preventative measures such as early identification of those individuals at risk. However, it was recognised that this relied on individuals being willing and feeling safe enough to disclose their health conditions, and line managers being more proactive in initiating such conversations within their teams, as well as being receptive to such discussions.

*“I think most organisations are guilty of not paying enough attention to something until it’s gone wrong. So, it’s all tertiary rehabilitation.”* [ID05, Wellbeing Lead & Occupational Health Technician]

A few also stated that even in a supportive workplace, making reasonable adjustments or gaining access to specialised resources was a lengthy process.

*“Xxx is really good at reasonable adjustments, but things do take a long time to happen.”* [ID08, Senior Clinical Research Practitioner]

Digital resources for self-management were the exception rather than the norm with only. one public sector organisation providing these. It offered mental health support via online yoga classes, the CALM application and a toolkit designed to help staff build psychological resilience but did not provide anything specifically for chronic pain.

In summary, stakeholders described organisational support as variable and often reactive, with a shared view that earlier and more consistent support for employees with chronic pain is needed.

#### Theme 2: not all line managers are equal.

This theme reflects variability in the role of line managers in supporting employees with chronic pain, highlighting differences in knowledge, attitudes, and willingness to provide effective support.

This theme comprised two subthemes:

a) Individual Variation refers to differences between line managers in terms of their attitudes, confidence, and willingness to engage and support employees experiencing chronic pain.
b) Supporting Line Managers captures the organisational structures and resources, if any, available to enable line managers to fulfil their role, including training, guidance, and support.

a) Individual Variation

As shown previously, there were many organisational roles involved in supporting the health and wellbeing of employees. However, stakeholders identified line managers as having a critical role in putting organisational values into practice at the local level.

*“Day-to-day they’re the person that has the biggest impact; they are the person that mediates the organisational values into your team. They’re the ones that are judging you. In want of a better word in terms of agreeing your objectives, and then how did, you do and if you do have any issues, they’re the ones that are supposed to be your first stopping point to get that support so they are hugely impactful.”* [ID15, Occupational Health & Wellbeing]

Approximately one third of stakeholders had management responsibilities or prior experience and felt that line managers generally tried to be supportive and make appropriate adjustments for their direct reports when required, although it was acknowledged that they had to balance this against what was practically feasible at work.

*“The managers are pretty good and pretty supportive in that because they’re involved in the process of referring that person in. They’re then more likely to listen to that advice because they’ve asked for it...doesn’t always work that way.”* [ID05, Wellbeing Lead & Occupational Health Technician]

Several commented that it could be challenging to meet individual needs without negatively impacting on the workload of other team members and also ensuring that everyone was appropriately supported. Furthermore, stakeholders observed that, as managers, they had to balance accommodations against work productivity.

*“And it means that other people are having to take the slack ultimately and increase their patients and increase their workload. And you know, a lot of the other team members might already be on the edge themselves of burnout or stress or had sickness leave, and that’s enough just to tip people over the edge.”* [ID14, Occupational Therapist]

*“It’s really difficult because as me, as [xxx] I would say, fine, you get up and stretch when you want you, you know you can move around. But for me as lead of the service you can’t do that, so it’s a balancing act between making reasonable adjustments that are helpful to the individual that don’t impact hugely on essentially productivity.”* [ID10, Service Lead]

Two individuals pointed out that, as line managers, they also needed support because managing individual adjustments in a team could be time intensive and sometimes challenging.

*“So, it depends as well on the workload of the line manager as to how far you can actually pursue that line of thought as well. So, if you have a team of five, you have more time to pursue you, if you’ve got a team of 50, that’s very tricky”.* [ID12, Lead Research Nurse]

Three quarters of stakeholders were concerned that the support line managers provided to their team varied depending on the individual manager and as a result support was inconsistent across the organisation. This was exacerbated by the fact that line managers were highly influential at all stages of the process; from an employee’s willingness to disclose, to providing reasonable adjustments and access to supportive services like occupational health. As mentioned previously, in the majority of cases, referrals such as to occupational health usually required the line manager to refer.

*“I would say that it’s hit and miss…So yeah, you know it depends on where you are, who your line manager is, the team that you work in and the job that you do, how much support is offered and what is available to you”.* [ID07, Head of Wellbeing]

*“The caveat I will put on it because it’s all about the delivery locally with the line manager. So, some line managers will look at the policy and go right every month I’m supposed to talk to everyone in my team about their health and wellbeing … However, in the real world, we have 4 1/2 thousand employees…So, do I think it happens exactly in exactly the same way in every team? No.”* [ID01, Head of Workforce Policy and Wellbeing]

Only a few organisations required line managers to be proactive and have regular (weekly or monthly) meetings with each team member which theoretically included checks on the employee’s health and wellbeing although it was not known if these took place. There were also a few cases where the organisation had developed guides/forms to help line managers have those potentially ‘difficult conversations’ about sickness absence, attendance, and adjustments and to signpost additional resources. One organisation had developed a toolkit specifically for managers above a certain level of seniority which was supplemented by a course which took place over several weeks. Another had designated representatives who attended all discussions between an individual and their line manager and acted as an advocate for the employee if required.

*“In terms of some of those wellbeing discussion tools can be quite helpful especially you know because it helps both sides have a structure and to help them work through that conversation.”* [ID15, Occupational Health & Wellbeing]

However, these kinds of early checks and balances were the exception rather than the rule and tools to support line managers in supporting their staff were not typically supplied by the organisation. Stakeholders recognised that line managers (like many other staff) typically did not understand chronic pain and were often inexperienced and under skilled in managing staff with complex, chronic conditions.

*“Another one of our huge priorities kind of moving forward is upscaling managers…a lot of our managers are excellent from an operational perspective. But we haven’t historically kind of provided the skills that you need to be a manager from the people perspective*.” [ID03, Head of Occupational Health & Wellbeing]

As a result of late disclosure and/or a lack of proactive engagement from line managers, the most likely scenario was that supportive interventions such as reasonable adjustments and occupational health were often only instigated when sickness absence had already become problematic.

*“We kind of catch up with the other wellbeing leads at the other [xx] networks quite regularly and they say pretty much the same thing; it needs that culture change to focus more on the prevention than just fixing things once they’ve gone wrong.”* [ID05, Wellbeing Lead & Occupational Health Technician]

*“I want to promote prevention much more as well, but it shouldn’t just be when people have been off sick and how do we support them to come back?”* [ID15, Occupational Health & Wellbeing]

Stakeholders recognised that individuals with chronic pain might hide this at work, being fearful to disclose symptoms which were invisible and often misunderstood and stigmatised. Some stakeholders also recognised that adjustments that worked for one employee might not help another and that the adjustments an individual required could vary over time as symptoms fluctuated. As a result, putting reasonable adjustments in place was perceived to be challenging.

*“It’s hard sometimes for managers to understand that how an employee presents could fluctuate without kind of rhyme nor reason, and so, for example, there are those symptoms which could deteriorate or improve without warning.”* [ID11, MSK & OH Physiotherapist]

Across stakeholder accounts, there was strong agreement that line manager support was highly variable and had a significant influence on employees’ access to and experience of workplace support. While flexible working and other adjustments were available, their implementation in practice was highly dependent on individual line managers, with variation in knowledge, capacity, and confidence shaping how effectively employees were supported.

(b) Supporting Line Managers

Stakeholder suggestions regarding support for line managers covered what content was needed and how it might be delivered.

During the discussion, some stakeholders spontaneously identified a need for additional resources to support managers – including in one case a version of the Pain-at-Work employees toolkit and this concept was hence explored further. Given the variability of line manager support and the lack of organisational tools, stakeholders reacted favourably to the concept of a developing a Pain-at-Work Toolkit specifically for managers hoping that a unified resource would educate managers and help them better support their staff. Stakeholders suggested that such a resource should include key information (e.g., conditions that can cause chronic pain, and the symptoms an employee might present with), guidance on communication and engagement (e.g., how to have constructive conversations with employees and practical examples (e.g., of reasonable adjustments and case studies showcasing how other organisations have supported staff to continue working, as well as signposting to additional resources. Individuals suggested that these resources should be aligned with the organisation’s specific policies and practices. These suggestions were intended to support managers, particularly those with limited experience or confidence in managing chronic conditions.

Stakeholders recognised that many of their suggestions reflected the current structure of the employee Pain-at-Work Toolkit [24], and an individual proposed this could be adapted to produce a managers version. This highlights a clear opportunity to extend existing resources to better support managers in their role.

*“Yes, short answer, yes. Long answer is yeah even at a basic level, which is advice… So yeah, so I’d definitely support that. It potentially could be digital, an online resource.”* [ID11, MSK & OH Physiotherapist]

*“When it comes to when it comes to support, there’s the age-old saying, isn’t here? You join an organisation; you leave the manager.”* [ID15, Occupational Health & Wellbeing]

*“As I said, we’ve got a lot of junior managers that are out there. I think they would find it (toolkit) useful because they’ve not got that experience, they haven’t got prior knowledge to draw on.”* [ID13, Practice Educator]

However, a few had reservations that managers would not have the time or inclination to engage with such resources.

*“You would get your sort of trailblazing managers who are incredibly passionate about staff welfare and wellbeing who would attend but I guess your managers who are preoccupied with all the other responsibilities they would be difficult to access.”* [ID09, Senior Occupational Health Advisor]

Stakeholders consistently emphasised the need for additional resources and training to support managers, particularly those with limited experience, in managing employees with chronic pain in the workplace.

#### Theme 3: Who Has Control?.

This theme explores how control over the management of chronic pain in the workplace is perceived and negotiated between individuals and organisations. Stakeholders described a dynamic interplay between personal responsibility and organisational influence, highlighting tensions around disclosure, self-management, and the provision of support.

This theme comprised two subthemes:

a) Individual Locus of Control refers to the extent to which employees are perceived as responsible for managing their own condition at work, including decisions around disclosure, seeking help, and engaging with available organisational support. This subtheme captures beliefs about personal agency, motivation, and capacity for self-management.
b) Organisational Culture reflects the broader organisational context that shapes how control is distributed and enacted, including norms around disclosure, expectations of employee responsibility, and the degree to which organisations create environments that enable or constrain support.

a) Individual Locus of Control

The extent to which an individual employee could self-manage their chronic pain at work was thought to vary. One theme that underpins many of the stakeholder comments is the influence of employees’ locus of control; that is, the extent to which individual employees feel they are in control of their pain. Locus of control is a psychological concept which refers to the individual’s perception that events are determined by their own behaviour rather than due to chance, luck or external factors [37]. According to Sowden, an internal locus of control is associated with a more pro-active approach to managing pain [38]. In this study, the concept can be applied to the extent an individual employee discloses their condition, actively seeks organisational help and support, and actively engages with this. The resources in the Pain-at-Work Toolkit were designed to encourage employees to self-manage their pain and support them in doing so. By providing employees with chronic pain self-management strategies the aim was to prevent individuals from working until they were at “breaking point” and/or taking repeated or extended sickness absence.

*“Trying to encourage people to take responsibility of the things that they can actually control, they can actually manage, I think are going to make a bigger difference than us just waiting for someone to come to us because they’re at breaking point.”* [ID05, Wellbeing Lead & Occupational Health Technician]

Stakeholders observed that there were differences in the way individuals would tackle the challenges of chronic pain, with some hiding their struggles, suffering in silence, and trying to push through. Individuals thought that older men were more likely to soldier on or that younger workers were more likely to ask for and/or expect the employer to find a solution.

*“It’s the kind of hidden thing, isn’t it? You kind of try and tone it down and say OK, I’ll just push through. I don’t want people to know that I’m finding that difficult.”* [ID15, Occupational Health & Wellbeing]

Several stakeholders felt that employees had to take some responsibility for self-managing their chronic pain. This included the need to disclose their condition, ask for help, and ideally state how the condition impacted their work and what they needed to help them stay in work.

*“I think there’s an individual responsibility as well, and that’s another thing we want to try and kind of push…and even if that is as far as saying, OK, admitting I’ve got pain, I should ask for help. That is again a message we want to try and get across.”* [ID03, Head of Occupational Health & Wellbeing]

*“I think the extent to which people self-manage pain varies, but I would like to see more of it. Of course, it depends on level and type of pain.”* [ID06 Occupational Health Adviser]

*“I think we live in a culture of dependency…And yeah, and we have a population that has forgotten how to self-care, and we’ve taught them that.”* [ID10 Service Lead]

Stakeholders generally agreed that while individual responsibility for self-management is important, employees vary in their capacity and willingness to engage with support.

b) Organisational Culture

Stakeholders recognised that their workplace culture significantly impacted employees, even those who proactively self-managed their condition, and admitted that organisations sometimes struggled to adapt organisational policies to individual needs. Stakeholders talked about employees being fearful of disclosing chronic pain, fearing that the employer would not be understanding, and as previously discussed, the attitude of an individual’s line manager was seen to be highly influential.

*“I guess people are somewhat reluctant to seek help and assistance, and they will quite happily kind of cover issues…I guess fear of disciplinary action would be a factor as well.”* [ID03, Head of Occupational Health & Wellbeing]

*“I think there’s a real fear that employers won’t be understanding. I think that’s real.”* [ID01, Head of Workforce Policy and Wellbeing]

Recognising that organisational support could be inconsistent, almost everyone felt that there was a need for additional tools to support employees in self-managing chronic pain at work. Stakeholders were generally positive towards digital resources providing education and support for self-management of chronic pain. However, a few noted that not everyone would be willing or able to access these and that additional modalities should be available.

*“It’s not a problem at all. I mean it’s (digital) where everything is. Where everything is, I think you can get better support.”* [ID02, Director]

*“I think even if you just factor in things like individual preference, neurodiversities as well as you need to factor in people’s familiarity with certain forms of media. So, for example older individuals might be less familiar with digital media.”* [ID03, Head of Occupational Health & Wellbeing]

There was broad agreement that organisational culture plays a critical role in shaping disclosure, access to support, and the effectiveness of self-management strategies.

## Overview of Stakeholders’ Responses to the Pain-at-Work Toolkit

Stakeholders from organisations in the intervention group of the Pain-at-Work feasibility trial [24] (n = 7 organisations, comprising eight stakeholders) were asked to comment on the implementation of the Pain-at-Work Toolkit within their organisation. As part of the feasibility trial, stakeholders had previously been involved in promoting the toolkit to employees. To support reflection during the interviews, stakeholders were provided with access to the toolkit and were invited to briefly remind themselves of its content prior to discussing their experiences and perceptions.

Positive responses about the Pain-at-Work Toolkit dominated, with the lived experience/case studies attracting commendation for their impact, and this and other content were believed to explain chronic pain and help people to better understand it. An individual specifically commented on the importance of including the impact of chronic pain on mental health and a couple of stakeholders also praised the content relating to reasonable adjustments and providing practical examples. Overall, the Pain-at-Work Toolkit appeared to systematise lots of useful information in one place and one well-informed stakeholder with lived experience of chronic pain commented that the resource even offered something new for those who were already knowledgeable about their condition. As mentioned previously, almost all stakeholders thought that there was a need for additional tools to support employees in self-managing chronic pain at work and believed that the Pain-at-Work Toolkit could address this unmet need.

*“The thing that this is filling is accepting that people can both be at work and in pain, and that is the reality is that people don’t want to be off sick all the time. They really want, obviously, ideally not to be in pain, but they have to work, and if they’re going to be at work, what are the things that they can be supported to either do for themselves or be supported by the organisation to mitigate the impact of that pain? And I don’t think there’s much really that speaks directly to that.”* [01 Head of Workforce Policy and Wellbeing]

Overall, the Pain-at-Work Toolkit was said to be comprehensive with stakeholders highlighting the value of existing content, such as the importance of planning and formalising reasonable adjustments in an individual’s work ‘passport’, as well as the role of pain medications (in pain management; stakeholders also debated the term ‘painkillers’, with some expressing concern that it could raise false expectations). The intervention aimed to facilitate functional improvement and enhance work engagement.

Negative comments were sparse and focused on ease of navigation, for example, toolkit users did not always notice the arrows which were used to move forward and back through the five sections. Otherwise only a few stakeholders suggesting minor amendments. These can be classed as either format or content related. Formatting included signposting or layering the information depending on the user’s prior knowledge of chronic pain and suggesting content based on the user’s trajectory. Suggested content included interactive elements to identify work triggers and patterns (for example, using pain diaries) and options to set work-related goals.

*“I think layering the information is something because many of our staff are extremely well informed and again, that’s probably not the norm but everybody found something. Everybody who looked found something new. But some of them had to go quite far in to find that new stuff. So, if it was kind of in strata’s, you know, basics, intermediate advanced.”* [ID02, Director]

## Discussion

This qualitative study comprised semi-structured interviews with 15 stakeholders from 12 organisations that had participated in a feasibility trial of the Pain-at-Work Toolkit [24]. The research questions explored the organisational culture around supporting people with chronic conditions (and specifically chronic pain) at work, the policies and practices in place and how the organisations in the intervention arm of the Pain-at-Work feasibility trial viewed the Pain-at-Work Toolkit as a mechanism for supporting individuals with chronic pain in the workplace.

Organisational stakeholders discussed the limitations in providing disability strategies for the workplace, the unique challenges in supporting employees with ‘non-visible’ disabilities, and the key role that line managers play in delivering organisational values, for example, in terms of providing and accessing support for their staff. While this aligns with prior research [4,5,14,39–46], our study makes a novel contribution by exposing the lack of specific provision for chronic pain in the workplace setting, and the challenges organisations face in meeting the needs of individuals with chronic pain.

As widely reported, non-visible disabilities such as chronic pain are often misunderstood and stigmatised [40,44–46]. Stakeholders acknowledged that the impact of these attitudes is that many employees were fearful of disclosing chronic conditions due to the perceived negative consequences at work such as fear of judgement, or concerns about how this may be perceived by managers and colleagues [47].

Stakeholders also demonstrated that as a non-visible disability, chronic pain is hidden on many levels. Across the themes; its prevalence was described as obscured within organisational systems for example since sickness absence data did not capture chronic pain explicitly. Support for chronic pain was embedded within broader policies rather than specifically addressed [14,39–41] and often inconsistently applied in practice. In addition, stakeholders highlighted that employees themselves may choose not to disclose chronic pain. Hence, while over one in four employees will experience chronic pain [48], stakeholder accounts in this study suggest that its true impact on both employee and organisations – particularly in terms of absenteeism, presenteeism and the individual’s mental and physical health is not fully recognised or systematically captured.

In common with other studies, we found that many organisations do not have disability workplace strategies and that, even in those that do, employees with chronic pain are not specifically accommodated for within general policies for other long-term or chronic conditions. This research also found that the predominantly large organisations included in the study commonly had a range of accommodations in place for long-term or chronic conditions, although not specifically for chronic pain. In line with a 2022 study [14], most organisations provided access to occupational health services. However, consistent with prior research [43], findings from our stakeholder study indicated that organisations tend to be ‘reactive’ rather than ‘proactive’ in dealing with chronic conditions in their workforce, with support typically initiated when frequent or extended sickness absence was already any issue. Furthermore, access to specialist support services, such as occupational health, was often slow and provision limited, for example, to one-off consultations or short-term treatments which limited the extent to which a complex condition like chronic pain could be effectively managed.

Despite its complexity, stakeholders frequently contextualised chronic pain as musculoskeletal. Notably, this framing, was common even among participants with clinical backgrounds and reasonable adjustments at work tended to focus on the employee’s physical environment, such as an enhanced Display Screen Equipment (DSE) assessment. Although flexible working patterns were sometimes provided, typically as part of reasonable adjustments, specific provisions for chronic pain tailored to the individual employee’s requirements were rarely evident.

While our stakeholders held organisational-level strategic and support roles, approximately a third also reported current or prior line management responsibilities. Both they and others recognised that managing employees who required significant and flexible workplace adjustments could be both time-intensive and, on occasion, emotionally draining. These individuals also spoke of the challenges in meeting organisational targets, for example, service delivery, while balancing the demands of reasonable adjustments for an individual with the workload and wellbeing of the broader team. However, stakeholders in the study readily acknowledged that line managers had a critical role in mediating organisational values and even in supportive organisations, local delivery of support to workers with chronic pain was not consistent. Stakeholder accounts highlighted the importance of line managers as an employee’s first point of contact when exploring reasonable adjustments in the workplace [14,49] and as ‘gatekeepers’ for employees accessing support [40,50,51]. Consistent with previous literature [47,52], variation in managers’ understanding and empathy – particularly toward non-visible disabilities such as chronic pain – was evident and influenced whether access to services such as occupational health was facilitated or hindered, sometimes described as a ‘line manager lottery’ [40].

As a result of this variability, three-quarters of stakeholders in our study identified line management as an area for improvement. This aligns with other studies that also identify a need for line manager training or resources [4,5]. Stakeholders emphasised the importance of better supporting managers through education about chronic pain and upskilling those with limited management experience who may find conversations with employees challenging.

Some stakeholders identified existing workplace resources, including guidance from professional bodies (e.g., ACAS, CIPD) and organisational websites (e.g., Business Disability Forum, Personnel Today, and the Reward and Employee Benefits Association). However, there was a clear interest in additional guidance and tools tailored to help managers support staff dealing with chronic pain in diverse work environments.

In addition to additional resources for managers, almost all participants in our stakeholder study felt that there was a need to better support employees to self-manage chronic pain. This appears to be compounded by gaps and variability in organisational support —including limited policies, inconsistent line manager support, and restricted access to services such as occupational health. Supporting self-management of chronic pain at work could benefit both employees and employers, however a 2022 study of 107 organisations found that the majority did not provide any self-management materials or education for people with chronic pain [14]. Furthermore, existing approaches to chronic pain self-management have typically focused on specific strategies (e.g., exercise or mindfulness) or particular conditions (e.g., low back pain), and existing digital interventions rarely address the challenges of managing chronic pain in the workplace. Stakeholders in our study emphasised the need for more comprehensive, workplace-relevant resources that incorporate a range of strategies and provide practical guidance for use in real-world work settings.

Reflecting these identified gaps in organisational resources for chronic pain, stakeholders in organisations that were randomised to the intervention arm of the feasibility trial were overwhelmingly positive towards the Pain-at-Work Toolkit [24] perceiving it as a comprehensive resource to support employees in self-managing chronic pain at work. In addition, stakeholder discussions highlighted a perceived gap in resources for line managers, suggesting a potential area for future development beyond the primary scope of this study. This represents a key target for implementation which we intend to address through the development of a targeted toolkit for managers, with potential to improve both employee wellbeing and organisational outcomes.

## Strengths & Limitations

This qualitative study drew on the experiences of 15 stakeholders with, on average, over five years’ experience in employee health and wellbeing. The interviews elicited rich, insightful data into organisational policies and provision for employees with chronic conditions specifically chronic pain. Reflexive thematic analysis enabled the research team to generate clear, consistent themes drawing on the invisibility of chronic pain, inequality in line management and locus of control. The study not only adds to the body of evidence into chronic conditions in the workplace but also provides novel findings situating and deepening our understanding of the challenges of managing and supporting employees with chronic pain at work.

However, stakeholders were all drawn from organisations who participated in the Pain-at-Work feasibility trial and as such may have an enhanced interest in employee health and wellbeing. Stakeholders were mostly from large organisations, defined as having > 250 employees with only one organisation of a smaller size. The findings may therefore not adequately reflect the views of stakeholders from small to medium-sized enterprises. However, the participant pool was defined by the composition of the larger feasibility trial cohort in terms of the type, size and sector of recruited organisations, and our contacts within them. The demographic profile of our sample (as reported in the Results, (mean age of 48.6 years and predominantly female) and minority representation from global ethnic majority backgrounds represents a limitation of the study. Further research is needed to capture the perspectives of younger stakeholders, male stakeholders, and those from more diverse ethnic backgrounds. Despite extensive notetaking, we had fewer verbatim quotes from the two stakeholders who did not consent to their interviews being recorded, which could have resulted in a loss of detail and nuance from those interviews [39].

## Implications for Policy and Practice

A greater understanding of chronic pain and hence more proactive identification and support might improve the speed and ease with which an employee could access organisational resource. In turn this expeditated pathway may facilitate more timely interventions and lead to better work related outcomes for employees with chronic pain and also their employers.

## Conclusion

This research highlights the challenges that employers face when looking to accommodate the needs of employees with chronic conditions specifically chronic pain and identifies some clear gaps in organisational resource. The Pain-at-Work Toolkit [24] which was co-created with diverse stakeholders including people living with chronic pain, healthcare professionals and employers [53] appears to address this gap and speak to the lack of digital interventions that focus on work-related self-management of chronic pain [54]. This digital toolkit offers resources to empower employees in managing their chronic pain, building their knowledge and confidence to navigate employee rights and access reasonable adjustments and support in the workplace. However, given the key role that managers play in providing and accessing support and in recognition of the complexities and challenges that managers face, a toolkit specifically for managers is currently being developed by the Pain-at-Work research team. A dyadic approach where both employer and employee have access to complementary guidance and resource may well provide a mechanism which benefits both parties by maximising the chances of an employee staying in the active workforce.

## Supporting information

S1 Table COREQ. (DOCX)

S2 Table 15 Point Checklist for Thematic Analysis. (DOCX)

S3 Text Interview Guide for Intervention Group Stakeholders. (DOCX)

S4 Text Interview Guide for Control Group Stakeholders. (DOCX)

S5 Table Qualitative Coding. (DOCX)
