# Supplementary material for: “Shining a light on chronic pain”: A qualitative study of stakeholder views towards chronic pain at work and the Pain-at-Work Toolkit
Source: PLoS One. 2026 Jul 2;21(7):e0351938. doi: 10.1371/journal.pone.0351938 (PMC13327183; doi:10.1371/journal.pone.0351938)
Supplement: S1 Table — (DOCX) [file pone.0351938.s001.docx]

**S1 Table: COREQ**

Consolidated criteria for reporting qualitative research (COREQ): a 32-item checklist for interviews and focus groups [Tong A, Sainsbury P, Craig J., 2007]

| **Topic and Item No.** | **Guide Questions/Description** | **Response** |
| --- | --- | --- |
| **Domain 1: Research team and reflexivity** | | |
| *Personal Characteristics* | | |
| 1. Interviewer/facilitator | Which author/s conducted the interview or focus group? | AT |
| 2. Credentials | What were the researcher’s credentials? E.g. PhD, MD | MSc and PhD. CCMRS |
| 3. Occupation | What was their occupation at the time of the study? | Researcher |
| 4. Gender | Was the researcher male or female? | Female |
| 5. Experience and training | What experience or training did the researcher have? | In addition to academic experience has over 35 years industry research experience. Specialist in qualitative research. Certified Member of Market Research Society, Previously on Board of British Healthcare Business Intelligence Association, and its Ethics Committee |
| *Relationship with participants* | | |
| 6. Relationship established | Was a relationship established prior to study commencement? | Researcher contacted potential participants via email at recruitment. No other relationship prior to study commencement |
| 7. Participant knowledge of the interviewer | What did the participants know about the researcher? e.g. personal goals, reasons for doing the research | Participants knew that the interviewer was a university researcher on the Pain-at-Work Trial working alongside HB and WJC. |
| 8. Interviewer characteristics | What characteristics were reported about the interviewer/facilitator? e.g. Bias, assumptions, reasons, and interests in the research topic | The interviewer was a White female, aged 59 years and was interested in the management of chronic pain in the workplace |
| **Domain 2: Study design** |  |  |
| *Theoretical framework* | | |
| 9. Methodological orientation and theory | What methodological orientation was stated to underpin the study? e.g. grounded theory, discourse analysis, ethnography, phenomenology, content analysis | Inductive reflexive thematic analysis |
| Participant selection | | |
| 10. Sampling | How were participants selected? e.g. purposive, convenience, consecutive, snowball | Purposive sample |
| 11. Method of approach | How were participants approached? e.g. face-to-face, telephone, mail, email | Participants were approached and recruited via an email from AT |
| 12. Sample size | How many participants were in the study? | 15 representing 12 organisations participating in Pain-at-Work Trial |
| 13. Non-participation | How many people refused to participate or dropped out? Reasons? | 1 participant withdrew from the study. 30 stakeholders were contacted and 15 agreed to participate |
| *Setting* | | |
| 14. Setting of data collection | Where was the data collected? e.g. home, clinic, workplace | Data were collected online via Microsoft Teams |
| 15. Presence of non-participants | Was anyone else present besides the participants and researchers? | No |
| 16. Description of sample | What are the important characteristics of the sample? e.g. demographic data, date | Organisational stakeholders identifying as having supportive role in health and wellbeing of workforce |
| *Data collection* | | |
| 17. Interview guide | Were questions, prompts, guides provided by the authors? Was it pilot tested? | Yes. The questioning guide was pilot tested with a colleague outside of the team |
| 18. Repeat interviews | Were repeat interviews carried out? If yes, how many? | No repeat interviews |
| 19. Audio/visual recording | Did the research use audio or visual recording to collect the data? | With the participant’s permission, interviews were audio and video-recorded using Microsoft Teams. 2 participants declined to be audio or video recorded so detailed notes were taken |
| 20. Field notes | Were field notes made during and/or after the interview or focus group? | Yes, by AT |
| 21. Duration | What was the duration of the interviews or focus group? | Interview length varied from 30 to 58 minutes with a mean of 44 minutes |
| 22. Data saturation | Was data saturation discussed? | Yes, but information power was used for assessment |
| 23. Transcripts returned | Were transcripts returned to participants for comment and/or correction? | No |
| **Domain 3: analysis and findings** |  |  |
| *Data analysis* | | |
| 24. Number of data coders | How many data coders coded the data? | One (coding and themes discussed with project team and external advisor who specialises in qualitative research) |
| 25. Description of the coding tree | Did authors provide a description of the coding tree? | No, but coding and development of themes was informed by data and reflexively refined |
| 26. Derivation of themes | Were themes identified in advance or derived from the data? | These were derived from the data (inductive approach) and reviewed with reference to existing literature on chronic pain |
| 27. Software | What software, if applicable, was used to manage the data? | None – analysis conducted manually in Microsoft Word due to AT’s preference |
| 28. Participant checking | Did participants provide feedback on the findings? | No |
| *Reporting* | | |
| 29. Quotations presented | Were participant quotations presented to illustrate the themes / findings? Was each quotation identified? e.g. participant number | Yes |
| 30. Data and findings consistent | Was there consistency between the data presented and the findings? | Yes |
| 31. Clarity of major themes | Were major themes clearly presented in the findings? | Yes |
| 32. Clarity of minor themes | Is there a description of diverse cases or discussion of minor themes? | Yes |
