# Supplementary material for: “Shining a light on chronic pain”: A qualitative study of stakeholder views towards chronic pain at work and the Pain-at-Work Toolkit
Source: PLoS One. 2026 Jul 2;21(7):e0351938. doi: 10.1371/journal.pone.0351938 (PMC13327183; doi:10.1371/journal.pone.0351938)
Supplement: S2 Table — (DOCX) [file pone.0351938.s002.docx]

**S2 Table: 15 Point Checklist for Thematic Analysis**

| **Process** | **Number** | **Criteria** |
| --- | --- | --- |
| Transcription | 1 | The data have been transcribed to an appropriate level of detail, and the transcripts have been checked against the tapes for ‘accuracy’**.** |
| Coding | 2 | Each data item has been given equal attention in the coding process. |
|  | 3 | Themes have not been generated from a few vivid examples (an anecdotal approach), but instead the coding process has been thorough, inclusive and comprehensive. |
|  | 4 | All relevant extracts for all each theme have been collated. |
|  | 5 | Themes have been checked against each other and back to the original data set. |
|  | 6 | Themes are internally coherent, consistent, and distinctive. |
| Analysis | 7 | Data have been analysed -/ interpreted, made sense of -/ rather than just paraphrased or described. |
|  | 8 | Analysis and data match each other -/ the extracts illustrate the analytic claims. |
|  | 9 | Analysis tells a convincing and well-organized story about the data and topic. |
|  | 10 | A good balance between analytic narrative and illustrative extracts is provided. |
| Overall | 11 | Enough time has been allocated to complete all phases of the analysis adequately, without rushing a phase or giving it a once-over-lightly. |
| Written Report | 12 | The assumptions about, and specific approach to, thematic analysis are clearly explicated. |
|  | 13 | There is a good fit between what you claim you do, and what you show you have done -/ i.e., described method and reported analysis are consistent. |
|  | 14 | The language and concepts used in the report are consistent with the epistemological position of the analysis**.** |
|  | 15 | The researcher is positioned as active in the research process; themes do not just emerge |

15 Point Checklist for Thematic Analysis [Braun V, Clarke V., 2006]
