# Supplementary material for: “Shining a light on chronic pain”: A qualitative study of stakeholder views towards chronic pain at work and the Pain-at-Work Toolkit
Source: PLoS One. 2026 Jul 2;21(7):e0351938. doi: 10.1371/journal.pone.0351938 (PMC13327183; doi:10.1371/journal.pone.0351938)
Supplement: S3 Text — (DOCX) [file pone.0351938.s003.docx]

**S3 Text: Interview Guide for Intervention Group Stakeholders**

**Introduction**: Hello, I am [name], a Project Researcher working on the Pain at Work (PAIN-AT-WORK) trial with [name] and [name]. Thank you for agreeing to talk to me. The interview will discuss organisational policies and practices to support someone with chronic or persistent pain and how the Pain at Work toolkit fits within your organisation.

**Consent:** Can I confirm that your informed consent is still valid and that you still wish to take part?

**Interview details:**  Participation is voluntary, and you can withdraw at any point without penalty. The interview will take around 45 to 60 minutes to complete but you can have a pause or breaks at any time.

With your permission, the interview will be recorded and then transcribed to make sure we have an accurate summary of what you have said. Recordings and transcripts and will be securely stored in password-protected computer files. Sometimes, I may use quotes that you provide during the interview in publications, but I will always make sure that you or your organisation would not be identifiable from these quotes.

**Confidentiality**: To confirm, everything you say during the interview will remain confidential; however, if you disclose a risk of harm to yourself or another person, I will need to report this to the appropriate person (Name - Study Chief Investigator).

**Rescheduling:** If we have a technical failure or lose contact, I will try to re-establish contact and wait for 10 minutes. After this time, I will email you to reschedule.

Do you have any questions before we begin?

**START THE RECORDING.**

Please can you confirm for the recording that you are happy to be recorded.

**MAIN INTERVIEW**

**SECTION 1: GENERAL PRACTICES FOR SUPPORTING STAFF WITH CHRONIC OR PERSISTENT PAIN**

I’d first like to ask you about policies and practices to support staff who have chronic conditions, and specifically chronic or persistent pain? I am asking about organisational policies generally not specifically about the PAIN-AT-WORK trial or toolkit.

**What policies and practices are in place to support people to self-manage chronic or persistent pain?**

**In your organisation, what might influence whether people self-manage their chronic or persistent pain?**

**How would you describe the organisational culture with respect to employees with chronic conditions, and specifically chronic or persistent pain?**

**What is your role in supporting people in your workplace who have chronic conditions, and specifically chronic or persistent pain?**

How long have you done this?

How many staff with chronic or persistent pain do you currently support?

What percentage of the total workforce would you say that is?

**Apart from yourself, who else is involved in providing support for people managing chronic or persistent pain at work?**

Internally e.g. occupational health, HR, trade union representatives

Externally

[If other people involved] - How do they provide support?

**Where do you find out more information to support people with chronic or persistent pain?**

Internal sources e.g. the organisation (which departments and/or colleagues, managers

External sources e.g. NHS website etc

**What is your experience of how people manage chronic or persistent pain or a painful condition at work?**

**To what extent, if at all, do you think people are able to self-manage their chronic or persistent pain at work?**

Why is that?

**To what extent, if at all, do you think there is a need for additional tools to support people in self-managing chronic or persistent pain at work?**

Why do you say that?

**How do you feel about the idea of supporting people at work with chronic or persistent pain/ chronic conditions through digital interventions (like the PAIN-AT-WORK toolkit), that they can use themselves to assist with self-management?**

Why do you say that?

**SECTION 2: PARTICIPATION IN THE TRIAL**

I’d like to ask you a few questions about how the trial itself.

**Why did you sign up to the trial in the first place?**

**How easy or difficult was it to get participation in the trial signed off?**

Why?

What barriers if any did you face?

[If barriers] – What would enable these to be overcome?

**Is there anything else that would encourage your organisation to participate in future trials like this?**

**SECTION 3: COMMUNICATING TO STAFF ABOUT THE PAIN-AT-WORK TOOLKIT**

Before we discuss the PAIN-AT-WORK toolkit in detail, let’s look at how the PAIN-AT-WORK toolkit was made available to employees.

**How did you inform your employees that the PAIN-AT-WORK toolkit was available?**

**Why did you choose this/these routes?** 

**Do you have any idea of how many people/what percentage of the workforce this information reached?**

**What difficulties, if any, did you face when communicating to employees about the PAIN-AT-WORK toolkit?**

**If you were involved in a similar trial in the future what, if anything, would you do differently in terms of reaching employees?**

Why?

**How would you feel about the PAIN-AT-WORK digital toolkit being sent directly to employees if they had signed up to receive it?**

Why do you say that?

**How might a direct delivery to employees work with staff members like yourself making it available?**

Check if both mechanisms would be preferable vs. Via Staff members only vs. Direct to employees only – Why?

**SECTION 4: EXPERIENCE OF STAFF USING THE PAIN-AT-WORK TOOLKIT**

**In your organisation what might have influenced whether people use the PAIN-AT-WORK toolkit or not?**

**How did you find the experience of supporting your staff to manage their chronic or persistent pain at work using the PAIN-AT-WORK toolkit?**

Positive, negative – why?

**What challenges, if any, came up as a result of any staff members using the PAIN-AT-WORK toolkit?**

Can you tell me more about these?

How did you get around these challenges, if at all?

**Overall, how well did the PAIN-AT-WORK toolkit fit with your organisation’s policies and practices?**

Why/why not?

**Has anything changed since your organisation joined the PAIN-AT-WORK Trial?**

Can you tell me more about the changes?

Positive, negative – why?

**In what ways, if at all, do you think that the PAIN-AT-WORK toolkit benefits people who experience chronic or persistent pain at work?**

**To what extent, if at all, do you think the PAIN-AT-WORK toolkit benefits people in terms of:**

Knowledge about self-managing their condition

Disclosure of disability at work

Their relationships at work/with colleagues

Their mental health and/or physical health

Their job satisfaction/ turnover intention

Work capacity/ productivity/ work ability

Sickness absence/ attending work despite being unwell or in more pain [presenteeism]

Work confidence/ motivation/ [Work self-efficacy]

**Did any members of staff share the PAIN-AT-WORK toolkit with you?**

[If not:] How helpful do you think that would have been for you to see the PAIN-AT-WORK toolkit? Why do you say that?

**SECTION 5: EVALUATION OF PAIN-AT-WORK TOOLKIT**

**Overall, what are your thoughts on the PAIN-AT-WORK toolkit?** *[If necessary, remind of content: the toolkit has 5 sections. These comprise Section 1: What is chronic or persistent pain, Section 2: Chronic or persistent pain and disability, Section 3: Work capacity, advice and support, Section 4: Self-management strategies, Section 5: Resources.*

*Can share link on Teams if required:* [https://www.nottingham.ac.uk/healthsciences/app-resources/pain-at-work/PAIN-AT-WORK-NHS4/](https://www.nottingham.ac.uk/healthsciences/app-resources/pain-at-work/PAW-NHS4/)

Overall positive or negative – why?

Strengths and weaknesses - ladder from features to benefits

Thoughts on the content

How well the idea was executed – why?

**Is there anything missing from the PAIN-AT-WORK toolkit?**

**Is there anything that you think should be included in the next iteration?**

**You said that... (there is/isn’t a need for additional tools to support people in self-managing chronic or persistent pain at work).**

**To what extent, if at all, do you think the PAIN-AT-WORK toolkit meets this need?**

Why?

**Is there anything else you would have wanted to know about the PAIN-AT-WORK toolkit to be able to support your staff member or members to use it?**

Why?

**How would you feel about a version of the PAIN-AT-WORK toolkit being made available to line managers?**

Why?

**On a scale of 1 to 10,** (where 1 is extremely unlikely and 10 is extremely likely? h**ow likely are you to recommend the PAIN-AT-WORK Toolkit to other people with chronic or persistent pain in your workplace?**

Why do you say that?

[If likely to recommend] – which job roles/ sectors/types of people, would you recommend it to?

[If not likely to recommend] - why not? What would encourage you to recommend it?

**On a scale of 1 to 10, how likely are you to recommend the PAIN-AT-WORK Toolkit to other organisations?**

Why do you say that?

Who do you think would benefit most? E.g. type of organisation

**Is there anything else you would like to share before I collect demographic data?**

**SECTION 6: DEMOGRAPHICS**

Can I just check your role/job title? (use contact information if known/relevant)

What is your age? (don’t ask - captured on consent form)

Which gender do you identify with?

Female

Male

Prefer to self-describe in another way (specify)

Prefer not to say

Please indicate your ethnic origin:

White

Black-Caribbean

Black-African

Black-Other

Indian

Pakistani

Bangladeshi

Chinese

Other (please specify)

**SECTION 7: CLOSING**

That’s the end of the main interview; thank you for your time. The audio recording will be fully transcribed, so we have an accurate record of this interview. Recordings and transcripts will be securely stored in password-protected computer files. The results from you and other participants will be analysed and the reports disseminated but you and your organisation will not be identified.

Would you like to receive a summary of the results when they are available which will be around the end of November?

Finally, may I ask if you would be willing to pass my contact details onto anyone else in organisation who has used the PAIN-AT-WORK toolkit to support an employee or employees.

**STOP RECORDING**
