# Supplementary material for: “Shining a light on chronic pain”: A qualitative study of stakeholder views towards chronic pain at work and the Pain-at-Work Toolkit
Source: PLoS One. 2026 Jul 2;21(7):e0351938. doi: 10.1371/journal.pone.0351938 (PMC13327183; doi:10.1371/journal.pone.0351938)
Supplement: S5 Table — (DOCX) [file pone.0351938.s005.docx]

# S5 Table. Qualitative Coding

# *Illustrative examples of coding framework and theme development*

Data extracts are mapped to initial codes, categories, and final themes to demonstrate the analytic process. Examples are illustrative and not exhaustive.

## Theme: Not All Disabilities Are Visible

| Data extract (illustrative) | Initial code | Category | Theme |
| --- | --- | --- | --- |
| “You don’t always know what somebody’s dealing with.” | Hidden conditions | Perceptions of chronic pain | Not All Disabilities Are Visible |
| “We don’t even have complete data on how many people would consider themselves disabled.” | Unknown prevalence | Perceptions of chronic pain | Not All Disabilities Are Visible |
| “It’s hard for managers to understand how symptoms can fluctuate.” | Unpredictable symptoms | Role and importance of line managers | Not All Disabilities Are Visible |
| “A lot of conditions are hidden… it’s harder for people to grasp.” | Invisible/hidden | Perceptions of chronic pain | Not All Disabilities Are Visible |
| “People are worried about being treated differently or penalised.” | Stigma and fear | Organisational culture | Not All Disabilities Are Visible |
| “If you talk about back pain people understand it better.” | Lack of understanding | Perceptions of chronic pain | Not All Disabilities Are Visible |

## Theme: Not All Line Managers Are Equal

| Data extract (illustrative) | Initial code | Category | Theme |
| --- | --- | --- | --- |
| “It depends on where you are and who your line manager is… it’s hit and miss.” | Variable attitudes/empathy | Role and importance of line managers | Not All Line Managers Are Equal |
| “Other people are having to take the slack… increasing workload.” | Impact on team members | Organisational culture | Not All Line Managers Are Equal |
| “It’s a balancing act between adjustments and productivity.” | Impact on output | Organisational culture | Not All Line Managers Are Equal |
| “We’ve got a lot of junior managers… they lack experience.” | Lack of experience | Role and importance of line managers | Not All Line Managers Are Equal |
| “How do we make sure support is consistent?” | Inconsistent support | Role and importance of line managers | Not All Line Managers Are Equal |
| “Providing training for managers is key.” | Need for training | Role and importance of line managers | Not All Line Managers Are Equal |

## Theme: Who Has Control?

| Data extract (illustrative) | Initial code | Category | Theme |
| --- | --- | --- | --- |
| “Organisations don’t pay attention until something goes wrong.” | Reactive approach | Organisational culture | Who Has Control? |
| “It depends on the person’s role and what adjustments are possible.” | Role dependency | Employee profile | Who Has Control? |
| “It depends on the level and type of pain.” | Pain variability | Employee profile | Who Has Control? |
| “Some people will suffer in silence and not disclose.” | Non-disclosure | Employee profile | Who Has Control? |
| “People are reluctant to seek help.” | Low support-seeking | Employee profile | Who Has Control? |
| “There is an individual responsibility to self-manage.” | Self-management capacity | Employee profile | Who Has Control? |
