# Supplementary material for: “Shining a light on chronic pain”: A qualitative study of stakeholder views towards chronic pain at work and the Pain-at-Work Toolkit
Source: PLoS One. 2026 Jul 2;21(7):e0351938. doi: 10.1371/journal.pone.0351938 (PMC13327183; doi:10.1371/journal.pone.0351938)
Supplement: Attachment — Submitted filename: Manuscript .docx [file pone.0351938.s006.docx]

# “Shining a light on chronic pain”: a qualitative study of stakeholder views towards chronic pain at work and the Pain-at-Work Toolkit

Angela Claire Thornton^1,3^, Elaine Wainwright^2^, Wendy J Chaplin^1,3^, Victoria Abbott-Fleming^4^, Daniel F McWilliams^5^, David A Walsh^5^, Gordon Taylor^6^, Paul McNamee^7^, Karen Walker-Bone^8^, Holly Blake^1,3^.

^1^School of Health Sciences, University of Nottingham, Nottingham, United Kingdom

^2^Aberdeen Centre for Arthritis and Musculoskeletal Health (Epidemiology Group), School of Medicine, Medical Sciences and Nutrition, University of Aberdeen, Aberdeen, United Kingdom

^3^NIHR Nottingham Biomedical Research Centre, Queens Medical Centre, Nottingham, UK.

^4^The Expert Patient and Carers Committee, British Pain Society, London, United Kingdom

^5^Injury, Recovery and Inflammation Sciences, School of Medicine, University of Nottingham, Nottingham, United Kingdom.

^6^ College of Medicine and Health, University of Exeter, Exeter, United Kingdom

^7^Health Economics Research Unit, Institute of Applied Health Sciences, University of Aberdeen, Aberdeen, United Kingdom

^8^Monash Centre for Occupational and Environmental Health, School of Public Health and Preventive Medicine, Monash University, Australia, United Kingdom

**Corresponding author:**

Professor Holly Blake, PhD CPsychol PFHEA FRSPH FBPsS

Professor of Behavioural Medicine

School of Health Sciences, University of Nottingham, Medical School,

Queen’s Medical Centre, Nottingham, NG7 2HA, UK.

Email: holly.blake@nottingham.ac.uk

Orcid ID: 0000-0003-3080-2306

# “Shining a light on chronic pain”: a qualitative study of stakeholder views towards chronic pain at work and the Pain-at-Work Toolkit.

**Abstract**

 Purpose

 The study explored the views of organisational stakeholders who participated in the feasibility trial of the Pain-at-Work Toolkit towards the implementation of the toolkit in ‘real-world’ workplace settings. This digital toolkit was co-created with healthcare professionals, employers, and people with chronic pain and aimed to inform and enable individuals to better self-manage their chronic pain at work.

Design/methodology/approach

 A qualitative study using semi-structured interviews with 15 stakeholders from 12 organisations that participated in a feasibility trial of the Pain-at-Work Toolkit. Purposive sampling was used to ensure the inclusion of stakeholders with management or supportive roles who have responsibility for the health and wellbeing of employees.

Findings

 The findings illuminate three key themes: not all disabilities are visible; not all line managers are equal; and who has control? These surmise that invisible disabilities such as chronic pain are underestimated, poorly understood, and inconsistently provisioned for in organisational policies. It highlights the key role that line managers play in employee disclosure and access to support but demonstrates that line managers vary in their delivery of support to employees. Lastly, it explores stakeholder perceptions of the roles of the employer relative to the employee with chronic pain. It confirms the need for additional resources to plug organisational gaps and give workers tools to self-manage their pain at work.

Originality

 This study indicates the need for resources /supports to upskill line managers so they can intervene to proactively support employees with chronic pain to reduce sickness absence and presenteeism (working when unwell). The research demonstrates organisational stakeholders’ interest in a multi-faceted approach to help employees self-manage chronic pain in all types at work, such as that provided by the Pain-at-Work Toolkit. In addition, it clearly identifies the potential for complementary resources to educate and facilitate line managers to better support their staff.

**Keywords**

Chronic pain, workforce, self-management, managers, organisations, digital health, occupational health

**Introduction**

In the United Kingdom, a systematic review and meta-analysis found that chronic pain - defined as pain that lasts or recurs for longer than 3 months [1] - affects 35% to 51% of the population, with a pooled estimate of 43.5% [2]. This equates to around 28 million adults, a figure predicted to increase, largely due to an ageing population. Looking specifically at the working population, the most recently available data, from the 2017 Health Survey for England [3], indicate that 27% of people in employment experience chronic pain.

Chronic pain may affect an individual’s ability to work [4, 5],[6] which can impact on both the employee and employer. For the individual, any extended absence from work can have a negative impact on their physical and mental health [7-9]. For the employer, productivity losses due to absenteeism [10] and presenteeism [11], as well as impacts on co-workers, can generate a significant financial burden [12, 13].

While there is a clear need to better support people with chronic pain at work. A recent survey study showed [14], almost half of organisations surveyed did not have disability policies; including those designed to support employees with chronic pain. Therefore, strategies that help individuals to self-manage, particularly those which can be applied at work, could offer important potential benefits [15]. Existing options typically focus on a specific approach, for example, exercise [16] or mindfulness [17] or a specific pain such as neck [18] or back pain [19] rather than offering a range of strategies that could be applied to any cause of chronic pain.

Digital interventions are already used to support the self-management of chronic pain [20] and have been shown to be effective in low back pain [21, 22] and musculoskeletal conditions [23]. However existing tools do not tend to focus specifically on workplace strategies or provide in depth resources. The Pain-at-Work Toolkit was developed to address this gap. This digital toolkit was co-created with healthcare professionals, employers and people with chronic pain [14] aiming to provide a comprehensive resource to enable the individual to better self-manage their pain at work, regardless of its cause.

An open-label, 2-arm multicentre pragmatic cluster-randomised feasibility trial of the Pain-at-Work Toolkit. This compared an intervention group with ‘treatment as usual’ controls in working adults with chronic pain in England [24]. This is ongoing and will be reported separately. In this paper, we report findings from an embedded qualitative interview study with organisational stakeholders, selected because they play a key role in health and wellbeing within the employing organisations participating in the trial. The study aimed to explore how individuals with chronic pain are supported in the workplace from the perspective of organisational stakeholders and examine the attitudes and experiences of organisational stakeholders.

**Methods**

Study Design

This was a qualitative interview study, with individual semi-structured interviews. Reporting aligns with the consolidated criteria for reporting qualitative research (COREQ) [25] (S1 Table). Since the aim was to explore the individual stakeholder’s views rather than gain a global perspective [26]; semi-structured interviews were conducted. This approach allowed the discussion to be focused on key topics while also allowing the researcher the flexibility and autonomy to explore relevant topics if and when they were raised [27].

Recruitment and Interview Schedule

The interview topic guides for both the intervention and control groups were developed by the project team (HB, WC, AT, EW) (S1 Text, S2 Text). A project researcher (AT) approached named organisational representatives from the Pain-at-Work feasibility trial via email in March 2025, at the end of their participation in the trial (6 months after baseline). If no initial response was received, up to two additional follow-up emails were sent in March and April 2025. At recruitment, potential stakeholders were informed about the study's objectives, format, and available interview dates. They were assured that entry into the study was entirely voluntary and that their employment would not be affected by their decision. Fieldwork was conducted between April 1, 2025, and May 27, 2025. Interviews were conducted at a mutually convenient time using videoconferencing hosted by Teams (Microsoft Corporation) and with one researcher (AT) who also took field notes. Written informed consent and demographic data were obtained via Smart Survey accessed through an email link with additional verbal consent audio-recorded prior to the interview. All but two of the interviews were audio and video recorded. Detailed notes were taken for the two stakeholders who did not consent for interviews to be recorded. Interviews lasted between 30 and 58 minutes with a mean of 44 minutes. Full interview transcripts were automatically generated in Microsoft Stream. Each transcript was checked by AT to ensure the interview content was accurately represented.

Stakeholders

The target population was individuals (stakeholders) employed in a role providing management or support for employees in an eligible organisation participating in the feasibility trial of the Pain-at-Work Toolkit [24]. Eligible organisations were defined as organisations located in England, from any sector (public, private or third) with 10 or more employees. Organisations we classed as small if they had 10-49 workers, medium-sized if 50-249, or large with 250 or above. A total of 30 individuals from the 18 participating organisations were invited to take part in an interview. These individuals played a key role in supporting employee health and wellbeing at the participating worksites. Fifteen stakeholders from 12 organisations consented to take part.

Ethics

The University of Nottingham Faculty of Medicine and Health Sciences Research Ethics Committee granted ethics approval on March 31, 2023 (FMHS 237-0323). Minor amendments were noted to file on 12.04.2023, 27.06.2023, 14.11.2023, 25.10.2024, 19.03.2025, 08.04.2025, and 04.08.2025. Our study protocol was reviewed and approved by HRA and Health and Care Research Wales (HCRW) on July 4, 2024 (IRAS 336655). The trial was prospectively registered on May 1, 2023 (ClinicalTrials.gov NCT05838677).

**Research Questions**

The research questions (RQs) were:

RQ1: What is the organisational culture around supporting people with chronic conditions at work?

RQ2: What policies and practices do organisations have in place to support people who have chronic conditions (and specifically chronic pain) at work?

RQ3: How do organisations perceive the Pain-at-Work Toolkit as a mechanism for supporting people with chronic pain at work?

Stakeholders were also asked to estimate the number of employees in the organisation and the percentage of workers with chronic pain in their organisation at recruitment into the trial and then at interview.

**Analysis**

Inductive data analysis following the principles of reflexive thematic analysis by Braun and Clarke was deployed [28]. This thematic analysis has six stages but the approach to analysis is organic and flexible whereby the researcher moves back and forward between the six phases rather than using a fixed, sequential approach [28-30]. The first step involved the researcher (AT) familiarising themselves with the data and since the same researcher (AT) interviewed stakeholders, checked transcripts, and collaboratively conducted analysis, they retained a close connection. AT took a data driven or inductive approach to generate initial codes which offered ‘answers’ to the key research questions. These generated preliminary themes which reflected patterns of shared meaning across interviews. These overarching themes were discussed and reviewed with two other researchers (HB and EW) and subsequently refined to ensure they accurately captured the data. These themes were then further defined, descriptively named and re-checked to ensure that they stood alone anchored by a unifying concept before findings were reported [31]. The analysis process was verified against Braun and Clarke’s 15-point checklist [32], to ensure the quality and rigour of thematic analysis (S2 Table). Analysis was conducted manually in Microsoft Word rather than using analysis software like NVivo. This decision was influenced by the researcher’s personal preference [33] and a desire to ensure the context of the qualitative data was retained [34]. Verbatims used in reporting were edited by AT to correct spelling and grammar generated by auto transcription but otherwise the original commentary was retained.

Reflexive thematic analysis places the researcher at the heart of the process rather than considering their subjectivity as bias or a lack of validity [28] and hence it is important to articulate the role of the researchers in the team. HB is a behavioural scientist with 30 years of experience in public health and applied health research. WJC and EW have a background in health psychology and a particular research interest in work and health, including chronic pain and work. AT has a background in psychology and over 35 years’ experience as an industry researcher specialising in healthcare.

**Information Power**

The sufficiency of the sample of 15 was assessed using the information power model where interview quality is more important than quantity [35]. In this model, Malteraud et. al., specify five interconnected factors which impact on information power in qualitative studies. These factors comprise 1) study aim, 2) sample specificity, 3) extent of established theory, 4) quality of dialogue between participant and researcher and 5) analysis strategy. In this instance, a larger purposive sample of 15 reflects the scope of the research questions and the specificity of the stakeholders who all hold pertinent roles looking after the health and wellbeing of the workforce. In addition, there is existing work on chronic pain in the workplace to underpin the data and boost information power. The research team perceived the quality of the dialogue to be high due to the experience of the researcher, and this is evident in the richness of the data [36]. The analysis strategy was to look across cases and ascertain realistic and pragmatic organisational policy. The 15 stakeholders came from a range of public and private sector organisations, even if information is limited from smaller organisations and those in the third or charity sector. On balance, we concluded that the qualitative sample of 15 had sufficient information power.

**Results**

Depending on the topic, results either refer to organisations (n=12) or stakeholders (n=15). Please note the final section summarising stakeholders’ responses relating to the Pain-at-Work Toolkit includes only the views of stakeholders from organisations that were allocated to the intervention group in the trial (n= seven organisations, eight stakeholders) since employees in the control group did not receive access to the toolkit. The data source is stated throughout.

Organisational Characteristics

Twelve organisations were recruited for this study, eleven of which were classified as large organisations, i.e. with >250 employees [37]. Seven organisations were cluster-randomised to the intervention arm of the trial and five to the control group. The sample included both public and private sector organisations, which were almost equally represented in the control group. In the intervention group, the public sector predominated. Details about the employment setting were collected from each organisation at two points. Firstly, at recruitment into the trial and then at the interview. However, in most cases the organisation representative (“gatekeeper”) who provided organisational consent for their worksite to be part of the trial was different to the interviewee.

Stakeholder Characteristics

Fifteen stakeholders were interviewed for this study: eight individuals from seven organisations in the intervention arm of the feasibility trial of the Pain-at-Work Toolkit and seven stakeholders from five organisations in the control group. Time in their current role ranged from a few months to 14 years, with an average of just over 5 years. Overall, the roles of stakeholders focused on wellbeing and occupational health, contributing to or leading strategy and policy around employee wellbeing. Duties included conducting employee assessments, giving advice about health and wellbeing and signposting, and/or supporting the employee to access other specialist services. These services included occupational health, physiotherapy, and a community pain clinic. Approximately one third of stakeholders also had line management responsibilities and drew on their experience when appropriate at the interview.

Thematic Analysis

As shown in Figure 1, three main themes (with six subthemes) were identified from stakeholders’ views of their organisational policies and procedures for employees with chronic pain. Each theme and subtheme, where appropriate, is discussed in turn and illustrated by anonymised quotations with participant identifier and role.

Figure 1: Themes

Theme 1: Not All Disabilities Are Visible

a) Policies and Provisions

This theme explored the extent of organisational policies and provisions for chronic conditions, including pain, and how visible and accessible these are for employees. It also covers stakeholders’ perceptions of how supportive the organisational culture is, as well as any gaps in provision. This theme addresses stakeholders’ motivations for participating in The Pain-at-Work Toolkit trial, specifically that invisible disabilities were often not disclosed or discussed. By participating in the feasibility trial, organisations hoped to send a clear message to all staff that chronic pain should be taken seriously and, ideally, accommodations be put in place before it resulted in long-term sickness absence, thus benefitting both the individual employee and the employer. There was a willingness to learn from the trial and the resources available within the toolkit and to use the results to inform evidence-based practice. Although this was only overtly stated by a couple of stakeholders, another factor was that by being involved in the trial, organisations would improve their corporate image and metrics.

*“Saying that to everybody across the organisation, whether you're an employee thinking about do I need to have a conversation about this or how am going to approach this conversation… but also if you're thinking about managers, if somebody is talking to you and having that conversation saying that the organisation treats that as something that's important and needs support”.* [ID15, Occupational Health & Wellbeing]

There were several organisational policies and provisions discussed (see Table 1).

Since organisational policies were self-reported rather than systematically audited, the list shown below (ordered by frequency of mention), is not necessarily exhaustive. The terminology below reflects stakeholder language.

Table 1: Policies and Provisions

| **Policy** | **Number of Organisations Providing (n=12):** |
| --- | --- |
| Reasonable Adjustments | 12 |
| Sickness Absence | 12 |
| **Support Services** |  |
| Occupational Health | 10 (majority – internal) |
| Health and Wellbeing | 8 |
| Physiotherapy | 8 |
| Employee Networks | 6 |
| Wellbeing/Work Adjustment Passport | 4 |
| Employee Assistance Programme (EAP) | 3 |

i)	Specific Policies for Chronic Pain

Although 11 of the 12 organisations were classified as large, none of the organisations surveyed had specific policies to support employees with chronic pain. Broader policies were mentioned, which aimed to support those with other long-term conditions or disabilities. Consequently, chronic pain was not clearly referred to in organisational policy or specifically provided for. Chronic pain was very much perceived to be a ‘hidden’ or ‘invisible disability’ which had implications for the support that employees received. This was reinforced by the finding that seven of the 15 stakeholders had no idea of the proportion of employees with chronic pain, and the remaining eight could at best guess at anything from occasional cases to a high proportion of the workforce.

*“I don't think there's anything specific just for pain. I think it is wrapped up with everything else. It does come under sort of long-term conditions.”* [ID07, Head of Wellbeing]

*“In terms of policies and practices, it depends on what you mean by where they sit. I don't think we still have anything that explicitly talks about it.”* [ID15, Occupational Health & Wellbeing]

Stakeholders acknowledged that invisible disabilities, such as chronic pain, which was often compared to mental health conditions, were poorly understood, could be stigmatised and received less support than visible disabilities like physical or sensory impairments.

*“Stigma around some people with conditions like fibro(myalgia) is still quite apparent, and that's even within healthcare, never mind a manager or an HR professional.”* [ID11, MSK & OH Physiotherapist]

*“If you can't see it, it isn't a broken leg. You haven't got a bad back; you know that sort of thing, or you’re not in a wheelchair…I really don't understand what the issue here is. And it's a bit like mental health. You know the two go hand in hand.”* [ID07, Head of Wellbeing]

ii)	Reasonable Adjustments

As required by the Equality Act 2010 [38], all 12 organisations made reasonable adjustments typically in conjunction with a personalised enhanced Display Screen Equipment (DSE) assessment often undertaken by the Health and Safety Team. Stakeholders were generally familiar with typical adjustments required for some types of chronic pain, mainly musculoskeletal. These included customised workstations such as ergonomic chairs, standing desks and adjustable footrests, and a few stakeholders specifically referenced the UK Government’s Access to Work scheme as a means of providing additional resources.

*“So, if it was about if it was about chronic pain it would depend on what kind of problem at work that was. But if it was about difficulty sitting for long periods of time then you might be directed to have an enhanced DSE assessment with the health and safety team.”* [ID01, Head of Workforce Policy and Wellbeing]

*“So, we do the DSE assessments, you know, so that's about your screen, your keyboard, your mouse, your chair, things like this.”* [ID12, Lead Research Nurse]

Adapted working patterns for employees with chronic pain, such as frequent breaks, flexible working hours, and/or hybrid working arrangements, were also provided. However, some stakeholders noted that it could be challenging to accommodate these changes while still meeting operational demands. This, together with other comments about limited resources and high workloads in support services, such as occupational health, highlights the real-world challenges of accommodating an employee with chronic pain.

*“A typical adjustment, say, for someone with an arthritic condition…whether that's in winter, cold conditions, or humid conditions, or if their pain is worse in the mornings is that they they're allowed to start later, or if they have the ability to do that, to be able to work from home to avoid having to do that commute”.* [ID14, Occupational Therapist]

*“So, if I give you an example of the of a tailored work plan for somebody with back pain. So, what we might do is we introduce rest breaks to move around, which is sort of 5 minutes or 10 minutes in between each patient. We might reduce the amount of driving so they can do a mixture of hybrid working… And we alter their working rota so that they're still seeing the same amount of patients or still having the same amount of patient contacts, but it's spread out a bit more. The difficulty is you still got patients to see as well.”* [ID10, Service Lead]

Some stakeholders recognised the need for individualised adjustments rather than a “blanket” solution but acknowledged this was not always reflected in policy and practice. One individual who had lived experience of chronic pain had themselves experienced a “one size fits all” approach at work.

*“But, you know, reasonable adjustments are, gosh, they're just so varied, aren't they?”* [ID12, Lead Research Nurse]

*“Myself and several colleagues all have lower back pain after childbirth. We are all very different but we all got a blanket set of advice.”* [ID08, Senior Clinical Research Practitioner]

Two stakeholders, each representing a different organisation (both third sector), demonstrated a particular willingness to provide a wide range of equipment in offices to help with disabilities, including walking pads, TENS machines and heated pads and blankets. In one case, a desire to accommodate the specific needs of workers with chronic pain was driven by the values and lived experience of one of the Directors. The other was working with estates and facilities to embed wellbeing into office design so that a range of needs were accommodated. This included considering how carpets impacted wheelchair users and chairs with arms for individuals who needed support to stand up, as well as chairs without arms for those who use walkers or sticks. These actions were the result of a proactive Occupational Health and Wellbeing Lead who had implemented learnings from a previous role. However, among stakeholders, this was the exception rather than the norm, with most organisations relying on standardised solutions.

iii)	Sickness Absence

All of the 12 organisations had what is officially termed a ‘sickness absence policy’ although this exact terminology was used by less than half of the 15 stakeholders; the most common language being ‘sickness’ or ‘sick with absence’ was less frequently used. The widespread use of sickness or sick may reflect unconscious attitudes—for example, framing sickness as the individual employee’s problem rather than something the organisation should take responsibility for - and some stakeholders indicated that they believed that people could do more to take responsibility for their own health. A few stakeholders spontaneously referenced chronic conditions (although not necessarily chronic pain). They stated that policies included the organisation’s expectations of both the employee and their line manager, as well as how sickness absence should be managed, particularly if it is extended or frequent. However, stakeholders acknowledged that this process was far from perfect, both in terms of it being punitive rather than supportive and that support was often only offered when sickness absence had become an issue for an employee’s line manager rather than aiming for early intervention (e.g., a reactive rather than proactive approach). One of the 12 organisations (third sector) also offered a ‘disability leave policy’ which, in addition to annual leave, allowed staff up to 5 paid days a year to attend medical appointments. This particular organisation was one of the two that provided a particularly wide range of accommodations for workers with disabilities.

*“We reviewed that (health and attendance policy) a year ago. But before it was primarily there to say how many days absence have you had. What is your line manager going to do about that and oh, you know the end of that is either you stop being absent or we sack you in a much more supportive way, but it wasn't very much focused on what can we do to stop people getting to the point where they need to absent themselves from work”.* [ID01 Head of Workforce Policy and Wellbeing]

*“I want to promote prevention much more as well; it shouldn't just be when people have been off sick and how do we support them to come back.”* [ID15, Occupational Health & Wellbeing]

Stakeholders were aware that chronic pain could present as a symptom of multiple long-term health conditions, including long COVID, fibromyalgia and musculoskeletal conditions such as lower back pain and rheumatoid arthritis. They also acknowledged that chronic pain frequently impacted on an individual’s ability to work. However, while chronic conditions such as fibromyalgia could be recorded by organisational systems, sickness absence reports were not set up in such a way that employees with chronic pain could be easily identified. Accurate data relied on an individual’s willingness to disclose chronic pain at work which can be influenced by several factors including the organisational culture and more specifically, as discussed subsequently, the individual’s line manager (Theme 2: Not All Line Managers Are Equal).

*“We don't have those figures and it's in part because of the way we record the statistic, because quite often with chronic pain, it's going to be something that comes up in conversation as a secondary order even if it actually turns out to be primary. I haven't found it really is something that people lead with, and so it won't be recorded necessarily either in their occupational health referral or with their sickness absence.”* [ID15, Occupational Health & Wellbeing]

*“So, we do have arthritis codified. We do have, you know, obviously injuries that occur in the workplace and I'll just scan down here to see what we’ve got. Chronic fatigue and fibromyalgia, connective tissue disorder. Let's have a look to see if we've got any chronic pain. We have muscles, skeletal disorder, broken down... fracture injury, lower and upper limb. Capture whether somebody's had surgery for orthopaedic reasons. So, it's not even close, we do have simply neurological. So yeah, so the answer is we don't capture, don't codify chronic or persistent pain.”* [ID09, Senior Occupational Health Advisor]

iv)	Occupational Health

Ten of the 12 organisations provided occupational health services, the majority of which were provided internally but this may reflect the sample composition of larger companies. Occupational Health was accessed mainly by referral from line managers and hence an individual’s line manager had considerable influence on employees’ access to support. Only two organisations allowed an employee to self-refer to occupational health; in the majority of cases this route was not available. Interestingly, one organisation had changed the process so that human resources rather than line managers could make staff referrals: the rationale being greater consistency and expertise; a theme which is explored in more detail later when we consider the impact of line managers on employee support at work (Theme 2: Not All Line Managers Are Equal).

*“I know in lots of organisations, then it might be the line manager that can do a direct referral to occupational health. As we're trying to get this consistency of support and as we're trying to support managers in how to approach this, we don't actually do that at xxx at the moment that got changed a couple of years ago. So, you would talk to my HR colleagues and they would do that referral so they would be able to mediate and support and help.”* [ID15, Occupational Health & Wellbeing]

Several stakeholders in occupational health felt that a lack of staff and restrictions on the amount of time they were able to spend with employees limited the support they could provide. While all but two of the 12 organisations offered occupational health services, there was noticeable variation in their capacity. Examples of the ratio of occupational health staff to other workers ranged from one occupational health worker for approximately 3,500 to 5,700 employees to one occupational health worker for every 250 staff.

*“I think there is something around expanding the remit of occupational health. So, you know, having more staff to enable us to be able to see people once, see people again a month later and then maybe meet with the manager as well to provide more ongoing follow-up, I don't think a one-off consultation report cuts it for people who have been living with chronic pain for potentially years”.* [ID14, Occupational Therapist]

v)	Physiotherapy

Eight of the 12 organisations also mentioned employees having access to physiotherapy, which in a few cases was subcontracted to an organisation called IPRS Health, and regardless of provider, was most often accessed by a referral from occupational health. Relatively little was discussed about the role physiotherapy could play in chronic pain although individuals noted that employees needed to be motivated to do the exercises recommended by the physiotherapist and that physiotherapy needed to be supported by changes in the working environment.

*“When it comes to sort of managing pain, usually there is quite a lot of input required, particularly with the main sort of processes that we push, say things like physiotherapy.”* [ID03, Head of Occupational Health & Wellbeing]

*“Staff have access to physios via self-referral or referral by Occupational Health. They are an external provider, but we closely manage the process, including holding records on site.”* [ID06, Occupational Health Advisor]

vi)	Employee Assistance Programme (EAP)

Three of the 12 organisations offered an Employee Assistance Programme (EAP) which provides independent resources and services such as counselling and advice on mental or physical health issues. Chronic pain was not mentioned specifically, so the ability of the programme to provide tailored advice is unknown.

*“People can call that, if they want to talk to someone about a medical concern they've got. That's staffed by a nursing team so they can get some support there.”* [ID01, Head of Workforce Policy and Wellbeing]

*“We do have an online Employee Assistance Programme which offers employees the opportunity to speak to somebody independent of the organisation for a range of advice topics, whether that be health-related or psychosocial issues.”*  [ID09, Senior Occupational Health Advisor]

vii)	Health and Wellbeing

Eight of the 12 organisations offer Health and Wellbeing, although the scope and form of this varies. Examples included regular events, such as presentations and webinars, from both internal staff and external experts, covering a range of topics. Wellbeing advice and signposts to resources were also typically available on the organisation’s intranet, and individuals mentioned specific campaigns, for example, aligned to national ‘awareness’ days or weeks. Versus Arthritis (a UK charity providing support for people with arthritis) and ‘Flippin’ Pain’ (a UK-based public health campaign focused on changing how people understand, talk about, and treat pain) were specifically mentioned by a couple of stakeholders as valuable resources for employees with chronic pain. Stakeholders from these organisations referred to staff who were designated as wellbeing champions or advocates whose role was to offer advice and support to colleagues. However, mental health conditions appeared to be given a higher profile, with musculoskeletal (not specifically chronic pain) only being mentioned twice.

viii)	Employee Networks

Six of the 12 organisations had employee-led networks as a means of providing a safe space for staff to discuss, share experiences, and offer support to one another. These spanned a wide range of groups, typically under the banner of Equality, Diversity, and Inclusion (EDI), including gender, sex, and sexual orientation, parental leave, working practices, and disability. Chronic pain was most likely to be covered in networks for people with disabilities and/or long-term health conditions rather than a specific forum.

*“We tried to listen to what, you know what our demographic wants and needs, and we tried to make sure that they have a safe space within the organisation that they feel represented in.”* [ID04, People Business Partner]

*So, for example, we have one which is titled xxx which is designed for employees with some form of underlying health issue or long-term health condition or you know those who find themselves subject to the need for workplace adjustments. It’s a peer-led work group.”* [ID09, Senior Occupational Health Advisor]

ix)	Wellbeing/Work Adjustment Passport

Four of the 12 organisations offered what was either described as a ‘Wellbeing or Work Adjustment Passport,’ which seems comparable to a Health Adjustment Passport (Department of Work and Pensions). The work document was primarily described as a means of providing a record of the employee’s needs and adjustments should they change roles and/or managers. In one instance, it was a response to numerous queries from employees with long-term conditions, including chronic pain. In a couple of cases, the document was also seen as a way of structuring a conversation between the employee and their line manager about health and wellbeing, for example, by exploring how the condition affected them, what triggered it, what the individual could do to support themselves, what they needed from their manager and the organisation and examples of what was possible. Although atypical, this extension of use may have merit, particularly given the important role that line managers play in supporting employees at work (Theme 2: Not All Line Managers Are Equal).

x)	Human Resources

Four of the 12 organisations mentioned Human Resources, although it seemed that some aspects of the role were shared with the Health and/or Wellbeing team.

*“So, for example, every month we will have a wellbeing conversation where every colleague's invited to join, they are all recorded if you can't join and we invite expert speakers in to speak, to talk to colleagues and that will be a range of topics, it will change every month. But sometimes we do have things that would support things around pain, you know, so physical wellbeing.”* [ID01, Head of Workforce Policy and Wellbeing]

*“We have a UK Health and Wellbeing Lead and a deputy lead to look after that mandate within the organisation. They run sort of supportive webinars for staff as well as sort of oversee or coordinate the various staff networks.”* [ID09, Senior Occupational Health Advisor]

Overall, while resources were widespread, there was little, if anything, specifically targeting chronic pain, and even these large organisations did not include digital tools in their armamentarium.

b) Organisational Support

Stakeholders’ comments indicated inconsistent support for employees with chronic pain, both across and within organisations, which is underpinned by a limited understanding of the nature of chronic pain and its potential impact on an employee’s working life. Six of the 12 organisations were said to be supportive of employees with chronic conditions, of which pain was often a component. However, in the remainder, stakeholders identified both strengths and weaknesses in the organisational culture. Inconsistent and inadequate support was attributed to two main factors. Firstly, health was often seen as a binary construct by employers, where individuals were either well enough to work or needed to be off sick, rather than a scenario where an employee could be supported to stay in work. Secondly, the unseen, unpredictable symptoms of conditions with chronic pain, such as fibromyalgia, were poorly understood and often stigmatised.

*“I definitely think physical impairment is probably more recognised and accepted than mental health and chronic pain because people just don't understand… If you can't see it, it isn't a broken leg. You haven't got a bad back; you know that sort of thing or you’re not in a wheelchair.”* [ID07, Head of Wellbeing]

*“I would say it is really similar sense to - we do a lot of work in terms of kind of mental health - and I kind of see a similarity there in that if something's not visually obvious, sometimes it can be really quickly dismissed.”* [ID03, Head of Occupational Health & Wellbeing]

*“And I think there's a very long time there's been a distinction, and especially this sort of mentality that we're challenging all the time in our managers of whether I'm fit enough to be at work or they're not.”* [ID01, Head of Workforce Policy and Wellbeing]

According to two representatives from the same organisation, one private sector company was very poor at supporting employees. This was attributed to the demographic of the workforce (typically older men) who were reluctant to disclose health issues. However, the culture was seen to be slowly improving, as evidenced by more employees seeking occupational health advice and support.

*“I think because you've got a culture of requirement to meet deadlines coupled with the culture of people not necessarily wanting to ask for help or a bit scared to kind of raise concerns.”* [ID03, Head of Occupational Health & Wellbeing]

Stakeholders felt that organisations needed to improve their support of employees with chronic pain. Currently, supportive intervention typically occurs when an employee’s attendance becomes an issue, but stakeholders want to see adjustments put in place earlier and, ideally, preventative measures such as early identification of those individuals at risk. However, this relied on individuals being willing and feeling safe enough to disclose their health conditions, and line managers being more proactive in initiating such conversations within their teams, as well as being receptive to such discussions.

*“I think most organisations are guilty of not paying enough attention to something until it's gone wrong. So, it's all tertiary rehabilitation.”* [ID05, Wellbeing Lead & Occupational Health Technician]

A few also stated that even in a supportive workplace, making reasonable adjustments or gaining access to specialised resources was a lengthy process.

*“Xxx is really good at reasonable adjustments, but things do take a long time to happen.”* [ID08, Senior Clinical Research Practitioner]

Digital resources for self-management were the exception rather than the norm. The one public sector organisation that offered these considered mental health via online yoga classes, the CALM application for mental health support and a toolkit designed to help staff build psychological resilience but did not provide anything for chronic pain.

Theme 2: Not All Line Managers Are Equal

a) Individual Variation

As shown previously, there were many organisational roles involved in supporting the health and wellbeing of employees. However, stakeholders identified line managers as having a critical role in putting organisational values into practice at the local level.

*“Day-to-day they're the person that has the biggest impact; they are the person that mediates the organisational values into your team. They're the ones that are judging you. In want of a better word in terms of agreeing your objectives, and then how did, you do and if you do have any issues, they're the ones that are supposed to be your first stopping point to get that support so they are hugely impactful.”* [ID15, Occupational Health & Wellbeing]

Approximately a third of stakeholders had management responsibilities themselves and felt that line managers generally tried to be supportive and make appropriate adjustments for their direct reports when required, although they had to balance this against what was practically feasible at work.

*“The managers are pretty good and pretty supportive in that because they're involved in the process of referring that person in. They're then more likely to listen to that advice because they've asked for it...doesn't always work that way.”* [ID05, Wellbeing Lead & Occupational Health Technician]

Several commented that it could be challenging to meet individual needs without negatively impacting on the workload of other team members and also ensuring that everyone was appropriately supported. Furthermore, they had to balance accommodations against work productivity.

*“And it means that other people are having to take the slack ultimately and increase their patients and increase their workload. And you know, a lot of the other team members might already be on the edge themselves of burnout or stress or had sickness leave, and that's enough just to tip people over the edge.”* [ID14, Occupational Therapist]

*“It's really difficult because as me, as xxx I would say, fine, you get up and stretch when you want you, you know you can move around. But for me as lead of the service you can't do that, so it's a balancing act between making reasonable adjustments that are helpful to the individual that don't impact hugely on essentially productivity.”* [ID10, Service Lead]

Two individuals pointed out that, as line managers, they also needed support because managing individual adjustments in a team could be time intensive and sometimes challenging.

*“So, it depends as well on the workload of the line manager as to how far you can actually pursue that line of thought as well. So, if you have a team of five, you have more time to pursue you, if you've got a team of 50, that's very tricky”.* [ID12, Lead Research Nurse]

Three quarters of stakeholders were concerned that the support line managers provided to their team varied depending on the individual manager and as a result support was inconsistent across the organisation. This was exacerbated by the fact that line managers were highly influential at all stages of the process; from an employee’s willingness to disclose, to providing reasonable adjustments and access to supportive services like occupational health. As mentioned previously, in the majority of cases, referrals such as to occupational health usually required the line manager to refer.

*“I would say that it's hit and miss…So yeah, you know it depends on where you are, who your line manager is, the team that you work in and the job that you do, how much support is offered and what is available to you”.* [ID07, Head of Wellbeing]

*“The caveat I will put on it because it's all about the delivery locally with the line manager. So, some line managers will look at the policy and go right every month I'm supposed to talk to everyone in my team about their health and wellbeing … However, in the real world, we have 4 1/2 thousand employees…So, do I think it happens exactly in exactly the same way in every team? No.”* [ID01, Head of Workforce Policy and Wellbeing]

Only a few organisations required line managers to be proactive and have regular (weekly or monthly) meetings with each team member which theoretically included checks on the employee’s health and wellbeing although it was not known if these took place. There were also a few cases where the organisation had developed guides/forms to help line managers have those potentially ‘difficult conversations’ about sickness absence, attendance, and adjustments and to signpost additional resources. One organisation had developed a toolkit specifically for managers above a certain level of seniority which was supplemented by a course which took place over several weeks. Another had designated representatives who attended all discussions between an individual and their line manager and acted as an advocate for the employee if required.

*“In terms of some of those wellbeing discussion tools can be quite helpful especially you know because it helps both sides have a structure and to help them work through that conversation.”* [ID15, Occupational Health & Wellbeing]

However, these kinds of early checks and balances were the exception rather than the rule and tools to support line managers in supporting their staff were not typically supplied by the organisation. Stakeholders recognised that line managers (like many other staff) typically did not understand chronic pain and were often inexperienced and under skilled in managing staff with complex, chronic conditions.

*“Another one of our huge priorities kind of moving forward is upscaling managers…a lot of our managers are excellent from an operational perspective. But we haven't historically kind of provided the skills that you need to be a manager from the people perspective*.” [ID03, Head of Occupational Health & Wellbeing]

As a result of late disclosure and/or a lack of proactive engagement from line managers, the most likely scenario was that supportive interventions such as reasonable adjustments and occupational health were often only instigated when sickness absence had already become problematic.

*“We kind of catch up with the other wellbeing leads at the other xx networks quite regularly and they say pretty much the same thing; it needs that culture change to focus more on the prevention than just fixing things once they've gone wrong.”* [ID05, Wellbeing Lead & Occupational Health Technician]

*“I want to promote prevention much more as well, but it shouldn't just be when people have been off sick and how do we support them to come back.”* [ID15, Occupational Health & Wellbeing]

Stakeholders recognised that individuals with chronic pain might hide this at work, being fearful to disclose symptoms which were invisible and often misunderstood and stigmatised. Some stakeholders also recognised that adjustments that worked for one employee might not help another and that the adjustments an individual required could vary over time as symptoms fluctuated. As a result, putting reasonable adjustments in place was perceived to be challenging.

*“It's hard sometimes for managers to understand that how an employee presents could fluctuate without kind of rhyme nor reason, and so, for example, there are those symptoms which could deteriorate or improve without warning.”* [ID11, MSK & OH Physiotherapist]

b) Supporting Line Managers

Given the variability of line manager support and the lack of organisational tools, stakeholders reacted favourably to the concept of a developing a Pain-at-Work Toolkit specifically for line managers hoping that a unified resource would educate line managers and help them better support their staff. Stakeholders suggested that such a resource should include basic concepts such as conditions that can cause chronic pain, which symptoms an employee might present with, how to engage constructively with employees with chronic, fluctuating conditions. Examples of reasonable adjustments could be provided and also case studies showcasing how others have supported staff to continue working, plus signposting resources /supports. Individuals suggested including the option to personalise the resources for the employee and reflect the organisation’s policies. Stakeholders recognised that many of their suggestions reflect the current structure of the employee Pain-at-Work Toolkit [24], and it was proposed this could be adapted to produce a Line Managers version.

*“Yes, short answer, yes. Long answer is yeah even at a basic level, which is advice… So yeah, so I'd definitely support that. It potentially could be digital, an online resource.”* [ID11, MSK & OH Physiotherapist]

*“I think that's an excellent idea because I think when it comes to when it comes to support, there's the age-old saying, isn't here? You join an organisation, you leave the manager.”* [ID15, Occupational Health & Wellbeing]

*“As I said, we've got a lot of junior managers that are out there. I think they would find it (toolkit) useful because they've not got that experience, they haven't got prior knowledge to draw on.”* [ID13, Practice Educator]

However, a few had reservations that managers would not have the time or inclination to engage with the resource.

*“You would get your sort of trailblazing managers who are incredibly passionate about staff welfare and wellbeing who would attend but I guess your managers who are preoccupied with all the other responsibilities they would be difficult to access.”*  [ID09, Senior Occupational Health Advisor]

Theme 3: Who Has Control?

a) Individual Locus of Control

The extent to which an individual employee could self-manage chronic pain at work was thought to vary. One theme that underpins many of the stakeholder comments is the influence of employees’ locus of control; that is, the extent to which individual employees feel they are in control of their pain. Locus of control is a psychological concept which refers to the individual's perception that events are determined by their own behaviour rather than due to chance, luck or external factors [39]. According to Sowden [40] an internal locus of control is associated with a more pro-active approach to managing pain. In this study, the concept can be applied to the extent an individual employee discloses their condition, actively seeks organisational help and support, and actively engages with this. It was intended that the resources in the Pain-at-Work Toolkit would encourage employees to self-manage their pain and support them in doing so. The intervention would provide employees with chronic pain self-management strategies to pace their activities, preventing individuals from working until they were at “breaking point” and/or taking repeated or extended sickness absence.

*“Trying to encourage people to take responsibility of the things that they can actually control, they can actually manage, I think are going to make a bigger difference than us just waiting for someone to come to us because they're at breaking point.”* [ID05, Wellbeing Lead & Occupational Health Technician]

Stakeholders observed that there were differences in the way individuals would tackle the challenges of chronic pain, with some hiding their struggles, suffering in silence, and trying to push through. Individuals thought that older men were more likely to soldier on or that younger workers were more likely to ask for and/or expect the employer to find a solution.

*“It's the kind of hidden thing, isn't it? You kind of try and tone it down and say OK, I'll just push through. I don't want people to know that I'm finding that difficult.”* [ID15, Occupational Health & Wellbeing]

Several stakeholders felt that employees had to take some responsibility for self-managing their chronic pain. This included the need to disclose their condition, ask for help, and ideally state how the condition impacted their work and what they needed to help them stay in work.

*“I think there's an individual responsibility as well, and that's another thing we want to try and kind of push…and even if that is as far as saying, OK, admitting I've got pain, I should ask for help. That is again a message we want to try and get across.”* [ID03, Head of Occupational Health & Wellbeing]

*“I think the extent to which people self-manage pain varies, but I would like to see more of it. Of course, it depends on level and type of pain.”* [ID06 Occupational Health Adviser]

*“I think we live in a culture of dependency…And yeah, and we have a population that has forgotten how to self-care, and we've taught them that.”* [ID10 Service Lead]

b) Organisational Culture

Stakeholders recognised that their workplace culture significantly impacted employees, even those who proactively self-managed their condition, and admitted that organisations sometimes struggled to adapt organisational policies to individual needs. Stakeholders talked about employees being fearful of disclosing chronic pain, fearing that the employer would not be understanding, and as previously discussed, the attitude of an individual’s line manager was highly influential in their experiences at work.

*“I guess people are somewhat reluctant to seek help and assistance, and they will quite happily kind of cover issues…I guess fear of disciplinary action would be a factor as well.”* [ID03, Head of Occupational Health & Wellbeing]

*“I think there's a real fear that employers won't be understanding. I think that's real.”* [ID01, Head of Workforce Policy and Wellbeing]

Recognising that organisational support could be inconsistent, almost everyone felt that there was a need for additional tools to support employees in self-managing chronic pain at work. Stakeholders were generally positive towards digital resources providing education and support for self-management of chronic pain. However, a few noted that not everyone would be willing or able to access these.

*“It’s not a problem at all. I mean it's (digital) where everything is. Where everything is, I think you can get better support.”* [ID02, Director]

*“I think even if you just factor in things like individual preference, neurodiversities as well as you need to factor in people's familiarity with certain forms of media. So, for example older individuals might be less familiar with digital media.”* [ID03, Head of Occupational Health & Wellbeing]

**Overview of Stakeholders’ Responses to the Pain-at-Work Toolkit**

Stakeholders from organisations in the intervention group of the Pain-at-Work feasibility trial (n = 7 organisations, comprising eight stakeholders) were asked to comment on the implementation of the Pain-at-Work Toolkit within their organisation. The findings will support the interpretation of the feasibility trial results and inform the design of a future definitive trial.

Positive responses about the Pain-at-Work Toolkit dominated, with the lived experience/case studies attracting commendation for their impact, and this and other content were believed to explain chronic pain and help people to better understand it. An individual specifically commented on the importance of including the impact of chronic pain on mental health and a couple of stakeholders also praised the content relating to reasonable adjustments and providing practical examples. Overall, the Pain-at-Work Toolkit appeared to systematise lots of useful information in one place and one well-informed stakeholder with lived experience of chronic pain commented that the resource even offered something new for those who were already knowledgeable about their condition. Almost all stakeholders thought that there was a need for additional tools to support employees in self-managing chronic pain at work and believed that the Pain-at-Work Toolkit seemed to address this unmet need.

*“The thing that this is filling is accepting that people can both be at work and in pain, and that is the reality is that people don't want to be off sick all the time. They really want, obviously, ideally not to be in pain, but they have to work, and if they're going to be at work, what are the things that they can be supported to either do for themselves or be supported by the organisation to mitigate the impact of that pain? And I don't think there's much really that speaks directly to that.”* [01 Head of Workforce Policy and Wellbeing]

Negative comments were sparse and focused on ease of navigation, for example, toolkit users didn’t always notice the ‘next’ arrows. Overall, the toolkit was said to be comprehensive, with only a few stakeholders suggesting minor amendments. These can be classed as either format or content related. Formatting included signposting or layering the information depending on the user’s prior knowledge of chronic pain and suggesting content based on the user’s trajectory. Suggested content included interactive elements to identify work triggers and patterns (for example, using pain diaries) and options to set work-related goals. Other views highlighted the value of existing content, such as the importance of planning and formalising reasonable adjustments in an individual’s work ‘passport’, as well as the role of pain medications (rather than ‘painkillers’, which could raise false expectations) and their role in pain management. The intervention aimed to facilitate functional improvement and enhance work engagement.

*“I think layering the information is something because many of our staff are extremely well informed and again, that's probably not the norm but everybody found something. Everybody who looked found something new. But some of them had to go quite far in to find that new stuff. So, if it was kind of in strata’s, you know, basics, intermediate advanced.”* [ID02, Director]

**Discussion**

This qualitative study comprised semi-structured interviews with 15 stakeholders from 12 organisations that had participated in a feasibility trial of the Pain-at-Work Toolkit. The research questions explored the organisational culture around supporting people with chronic conditions at work and what policies and practices organisations have in place to support people who have chronic conditions (and specifically chronic pain) at work. In addition, the study examined how participating organisations utilised the Pain-at-Work Toolkit as a mechanism for supporting individuals with chronic pain in the workplace. Organisational stakeholders discussed the limitations in providing disability strategies for the workplace, the unique challenges in supporting employees with ‘non-visible’ disabilities, and the key role that line managers play in delivering organisational values, for example, in terms of providing and accessing support for their staff. While this aligns with prior research [4, 5, 14, 41-48], our study makes a novel contribution by exposing the lack of specific provision for chronic pain in the workplace setting, and the challenges organisations face in meeting the needs of individuals with chronic pain. Importantly, we share stakeholder’s views on the Pain-at-Work Toolkit as one mechanism for addressing this gap in provisions. Overall, there was a lack of understanding of complex non-visible disabilities like chronic pain and insufficient resources to support an individual at work effectively. An awareness of the gaps in organisational resources for chronic pain means that stakeholders welcomed The Pain-at-Work Toolkit as a comprehensive resource to support an employee in self-managing their condition in the workplace.

In common with other studies, we found that many organisations do not have disability workplace strategies and that employees with chronic pain are not specifically accommodated for within general policies for other long-term or chronic conditions [14, 41-43]. The 2017 Health Survey for England [3], indicated that 27% of people in employment experience chronic pain. However, even these stakeholders - who were all actively involved in the health and wellbeing of the organisation’s workforce – did not have organisational data on the prevalence of chronic pain. Stakeholders demonstrated that as a non-visible disability [44], chronic pain is hidden on many levels; its prevalence is hidden among sickness absence data, employee support is hidden within organisational policies and chronic pain is also often hidden by the employees themselves. Hence, while over one in four employees will experience chronic pain [3], the true impact of this on both employee and employer in terms of absenteeism, presenteeism and the individual’s mental and physical health is not fully recognised or accounted for.

This research confirms that large organisations commonly have a range of accommodations in place for long-term or chronic conditions, although not specifically for chronic pain. In line with a 2022 study [14], most organisations provided access to occupational health services (albeit these were mostly large organisations, not SMEs). However, comparable with findings from prior research using an online survey and qualitative interviews [45], our research revealed that organisations tend to be ‘reactive’ rather than ‘proactive’ in dealing with chronic conditions in their workforce, only intervening when the employee was already taking frequent or extended sickness absence. Furthermore, access to specialist support services, such as occupational health, can be slow and provision limited, for example, to one-off consultations or short-term treatments. This is understandable given the demand for such services. Still, it is unlikely to allow a condition like chronic pain, which can become a separate condition, more complex than the pain caused by the original injury or disease [49] to be unpacked and effectively managed.

In spite of its complexity, stakeholders typically contextualised chronic pain as musculoskeletal and reasonable adjustments at work tended to focus on the employee’s physical environment, such as an enhanced Display Screen Equipment (DSE) assessment. Although flexible working patterns were sometimes provided, specific provisions for chronic pain tailored to the individual employee’s requirements were rarely evident. As reported elsewhere, non-visible disabilities such as chronic pain are often misunderstood and stigmatised [42, 46-48]. Stakeholders acknowledged that the impact of these attitudes is that many employees were fearful of disclosing chronic conditions due to the perceived negative consequences at work [50].

While our stakeholders held organisational-level support roles, a third of them were also line managers themselves or had prior management experience. Both they and others recognised that managing employees who required significant and flexible workplace adjustments could be both time-intensive and, on occasion, emotionally draining.’ These individuals also spoke of the challenges in meeting organisational targets, for example, for service delivery and the need to balance the demands of reasonable adjustments for an individual with the workload and wellbeing of the broader team. However, stakeholders in the study readily acknowledged that line managers had a critical role in mediating organisational values and even in supportive organisations, local delivery of support to workers with chronic pain was not consistent [50, 51].

Line managers are typically the individual’s first point of contact when exploring reasonable adjustments in the workplace [14, 52]; and they also act as a ‘gatekeeper’ for accessing support [42, 53, 54]. Depending on their understanding and empathy for the impact of chronic pain on work, line managers can either help or hinder access to supportive services such as occupational health, described by employees in one study as the ‘line manager lottery’ [42]. Prior research among employees indicated that line managers’ awareness, understanding and attitude towards chronic conditions such as pain varies considerably, and managers who have experienced a chronic condition themselves are often more willing to assist with access to support than those who have had no lived experience [42]. However, three-quarters of stakeholders in our study identified line management as an area for improvement. This aligns with other studies [4,5] that also identify a need for line manager training or resources. Stakeholders acknowledged that some line managers were less than sympathetic and supportive to hidden disabilities like chronic pain and did not understand the impact it could have on an individual’s life at home and at work. Many spoke of the need to educate line managers about chronic pain and how to best support employees, as well as to upskill those with limited management experience who might find such conversations challenging. Guidance on managing employees with chronic pain is currently limited to that provided by professional bodies (e.g., ACAS, CIPD) or on various organisational websites (e.g., Business Disability Forum, Personnel Today, and the Reward and Employee Benefits Association). Hence there is a clear need for guidance and tools tailored to help managers support staff dealing with chronic pain in diverse work environments.

Given the ‘gaps’ in organisational support, assisting people to self-manage chronic pain at work could benefit both the individual employees and improve organisational outcomes. However, a 2022 study of 107 organisations found that the majority did not provide any self-management materials or education for people with chronic pain [14]. Almost all participants in our stakeholder study felt that there was a need for additional tools although it is readily acknowledged that these individuals have chosen to be part of the trial and may not be representative of all stakeholders. A 2019 study found that more than half of employers were reactive rather than proactive when supporting employees with chronic pain. [14]. However, The Pain-at-Work Toolkit feasibility trial [24] was commended for ‘shining a light’ on the impact of chronic pain in the workplace and highlighting the importance of prevention and early intervention before repeated or prolonged sickness absence. The organisations that were randomised to the intervention arm of the trial were overwhelmingly positive towards the Pain-at-Work Toolkit [24], feeling it offered a comprehensive resource to enable employees to self-manage chronic pain at work. Stakeholders also reacted positively to the concept of producing resources specifically for line managers which would complement the existing employee-facing Pain-at-Work Toolkit.

**Strengths & Limitations:**

This qualitative study drew on the experiences of 15 stakeholders with on average over five years’ experience in employee health and wellbeing. The interviews elicited rich, insightful data into organisational policies and provision for employees with chronic conditions specifically chronic pain. Reflexive thematic analysis enabled the research team to generate clear, consistent themes drawing on the invisibility of chronic pain, inequality in line management and locus of control. The study not only adds to the body of evidence into chronic conditions in the workplace but also provides novel findings situating and deepening our understanding of the challenges of managing and supporting employees with chronic pain at work.

However, stakeholders were all drawn from organisations who participated in the Pain-at-Work feasibility trial and as such may have an enhanced interest in employee health and wellbeing. Stakeholders were mostly from large organisations, defined as having > 250 employees [37] with only one organisation of a smaller size. The findings may therefore not adequately reflect the views of stakeholders from small to medium-sized enterprises. However, the participant pool was defined by the composition of the larger feasibility trial [24] in terms of the type, size and sector of recruited organisations, and our contacts within them. Given the demographics of our sample, further research would be needed to understand the views of younger stakeholders (our stakeholders had a mean age of 48.6 years which might be expected for their level of seniority), male stakeholders (the majority in our study were female) and stakeholders from global ethnic majority backgrounds. Despite extensive notetaking, we had fewer verbatim quotes from the two stakeholders who did not consent to their interviews being recorded, which could have resulted in a loss of detail and nuance from those interviews [41]

**Implications for Policy and Practice:**

A greater understanding of chronic pain and hence more proactive identification and support might improve the speed and ease with which an employee could access organisational resource. In turn this expeditated pathway may facilitate more timely interventions and lead to better work related outcomes for employees with chronic pain and also their employers.

**Conclusion**

This research highlights the challenges that employers face when looking to accommodate the needs of employees with chronic conditions specifically chronic pain and identifies some clear gaps in organisational resource. The Pain-at-Work Toolkit [14 and 24] which was co-created with diverse stakeholders including people living with chronic pain, healthcare professionals and employers appears to address this gap and speak to the lack of digital interventions that focus on work-related self-management of chronic pain [55]. This digital toolkit offers resources to empower employees in managing their chronic pain, building their knowledge and confidence to navigate employee rights and access reasonable adjustments and support in the workplace. However, given the key role that line managers play in providing and accessing support and in recognition of the complexities and challenges that managers face, a toolkit specifically for these key stakeholders is currently being developed by the Pain-at-Work team. A dyadic approach where both employer and employee have access to complementary guidance and resource may well provide a mechanism which benefits both parties by maximising the chances of an employee staying in the active workforce.

**References**

1.	Barke A, Korwisi B, Jakob R, Konstanjsek N, Rief W, Treede R-D. Classification of chronic pain for the International Classification of Diseases (ICD-11): results of the 2017 international World Health Organization field testing. PAIN. 2022;163(2).

2.	Fayaz A, Croft P, Langford RM, Donaldson LJ, Jones GT. Prevalence of chronic pain in the UK: a systematic review and meta-analysis of population studies. BMJ Open. 2016;6(6):e010364.

3.	University College London DoEaPH, National Centre for Social Research. Health Survey for England, 20172017 27 June 2025. Available from: <http://doi.org/10.5255/UKDA-SN-8488-3>.

4.	Toye F, Kate S, Nick A, Michelle B, Eloise C, and Barker K. A synthesis of qualitative research exploring the barriers to staying in work with chronic musculoskeletal pain. Disability and Rehabilitation. 2016;38(6):566-72.

5.	Johannes CB, Le TK, Zhou X, Johnston JA, Dworkin RH. The Prevalence of Chronic Pain in United States Adults: Results of an Internet-Based Survey. The Journal of Pain. 2010;11(11):1230-9.

6.	Breivik H, Collett B, Ventafridda V, Cohen R, Gallacher D. Survey of chronic pain in Europe: Prevalence, impact on daily life, and treatment. European Journal of Pain. 2006;10(4):287-.

7.	Paul KI, Moser K. Unemployment impairs mental health: Meta-analyses. Journal of Vocational Behavior. 2009;74(3):264-82.

8.	Bryan ML, Bryce AM, Roberts J. The effect of mental and physical health problems on sickness absence. The European Journal of Health Economics. 2021;22(9):1519-33.

9.	Sieurin L, Josephson M, Ving�Rd EVA. Positive and negative consequences of sick leave for the individual, with special focus on part-time sick leave. Scandinavian Journal of Public Health. 2009;37(1):50-6.

10.	Agaliotis M, Mackey MG, Jan S, Fransen M. Burden of reduced work productivity among people with chronic knee pain: a systematic review. Occupational and Environmental Medicine. 2014;71(9):651.

11.	Patel AS, Farquharson R, Carroll D, Moore A, Phillips CJ, Taylor RS, et al. The Impact and Burden of Chronic Pain in the Workplace: A Qualitative Systematic Review. Pain Practice. 2012;12(7):578-89.

12.	Kronborg C, Handberg G, Axelsen F. Health care costs, work productivity and activity impairment in non-malignant chronic pain patients. The European Journal of Health Economics. 2009;10(1):5-13.

13.	Maniadakis N, Gray A. The economic burden of back pain in the UK. PAIN. 2000;84(1):95-103.

14.	Blake H, Somerset S, Greaves S. The Pain at Work Toolkit for Employees with Chronic or Persistent Pain: A Collaborative-Participatory Study. Healthcare. 2022;10(1):56.

15.	Shaw WS, H. TT, Mary G-L, Yueng-Hsiang H, K. NM, E. RS, et al. Adapting principles of chronic pain self-management to the workplace. Disability and Rehabilitation. 2012;34(8):694-703.

16.	Andersen LL, Persson R, Jakobsen MD, Sundstrup E. Psychosocial effects of workplace physical exercise among workers with chronic pain: Randomized controlled trial. Medicine. 2017;96(1).

17.	Hilton L, Hempel S, Ewing BA, Apaydin E, Xenakis L, Newberry S, et al. Mindfulness Meditation for Chronic Pain: Systematic Review and Meta-analysis. Annals of Behavioral Medicine. 2016;51(2):199-213.

18.	Aas RW, Tuntland H, Holte KA, Røe C, Lund T, Marklund S, et al. Workplace interventions for neck pain in workers. Cochrane Database of Systematic Reviews. 2011(4).

19.	Bell JA, Burnett A. Exercise for the Primary, Secondary and Tertiary Prevention of Low Back Pain in the Workplace: A Systematic Review. Journal of Occupational Rehabilitation. 2009;19(1):8-24.

20.	Macea DD, Gajos K, Daglia Calil YA, Fregni F. The Efficacy of Web-Based Cognitive Behavioral Interventions for Chronic Pain: A Systematic Review and Meta-Analysis. The Journal of Pain. 2010;11(10):917-29.

21.	Du S, Liu W, Cai S, Hu Y, Dong J. The efficacy of e-health in the self-management of chronic low back pain: A meta analysis. International Journal of Nursing Studies. 2020;106:103507.

22.	Garg S, Garg D, Turin TC, Chowdhury MFU. Web-Based Interventions for Chronic Back Pain: A Systematic Review. J Med Internet Res. 2016;18(7):e139.

23.	Slater H, Stinson JN, Jordan JE, Chua J, Low B, Lalloo C, et al. Evaluation of Digital Technologies Tailored to Support Young People’s Self-Management of Musculoskeletal Pain: Mixed Methods Study. J Med Internet Res. 2020;22(6):e18315.

24.	Blake H, Chaplin WJ, Wainwright E, Taylor G, McNamee P, McWilliams D, et al. The Web-Based Pain-at-Work Toolkit With Telephone Support for Employees With Chronic or Persistent Pain: Protocol for a Cluster Randomized Feasibility Trial. JMIR Res Protoc. 2023;12:e51474.

25.	Tong A, Sainsbury P, Craig J. Consolidated criteria for reporting qualitative research (COREQ): a 32-item checklist for interviews and focus groups. International Journal for Quality in Health Care. 2007;19(6):349-57.

26.	McGrath C, J. PP, and Liljedahl M. Twelve tips for conducting qualitative research interviews. Medical Teacher. 2019;41(9):1002-6.

27.	Adeoye-Olatunde OA, Olenik NL. Research and scholarly methods: Semi-structured interviews. JACCP: JOURNAL OF THE AMERICAN COLLEGE OF CLINICAL PHARMACY. 2021;4(10):1358-67.

28.	Braun V, and Clarke V. Reflecting on reflexive thematic analysis. Qualitative Research in Sport, Exercise and Health. 2019;11(4):589-97.

29.	Braun V, Clarke V. Successful qualitative research: A practical guide for beginners. 2013.

30.	Braun V, Clarke V. One size fits all? What counts as quality practice in (reflexive) thematic analysis? Qualitative Research in Psychology. 2021;18(3):328-52.

31.	Byrne D. A worked example of Braun and Clarke’s approach to reflexive thematic analysis. Quality & Quantity. 2022;56(3):1391-412.

32.	Braun V, Clarke V. Using thematic analysis in psychology. Qualitative Research in Psychology. 2006;3(2):77-101.

33.	Mattimoe R, Hayden M, Murphy B, Ballantine J. Approaches to analysis of qualitative research data: A reflection on the manual and technological approaches. Accounting, Finance & Governance Review. 2021;27(1):1-16.

34.	Thompson SM, Barrett PA. Summary oral reflective analysis: a method for interview data analysis in feminist qualitative research. ANS Adv Nurs Sci. 1997;20(2):55-65.

35.	Malterud K, Siersma VD, Guassora AD. Sample Size in Qualitative Interview Studies: Guided by Information Power. Qual Health Res. 2016;26(13):1753-60.

36.	LaDonna KA, Artino AR, Jr., Balmer DF. Beyond the Guise of Saturation: Rigor and Qualitative Interview Data. J Grad Med Educ. 2021;13(5):607-11.

37.	OECD. Main Economic Indicator2023 27 June 2025. Available from: <https://www.oecd.org/en/data/indicators/enterprises-by-business-size.html>). .

38.	Equality Act. 2010.

39.	Coughlin AM, Badura AS, Fleischer TD, Guck TP. Multidisciplinary treatment of chronic pain patients: its efficacy in changing patient locus of control. Archives of physical medicine and rehabilitation. 2000;81(6):739-40.

40.	Sowden M, Hatch A, Gray SE, Coombs J. Can four key psychosocial risk factors for chronic pain and disability (Yellow Flags) be modified by a pain management programme?: A pilot study. Physiotherapy. 2006;92(1):43-9.

41.	Cordero J, de Zúñiga TO, Rueda M. Disability and corporate social responsibility reporting: An analysis comparing reporting practices of 40 selected multinational enterprises: ILO; 2014.

42.	Blake H, Giannoulatou M, Chaplin WJ. Employees’ experiences of chronic pain in the workplace. Occupational Medicine. 2025.

43.	Khan N, Korac-Kakabadse N, Skouloudis A, Dimopoulos A. Diversity in the workplace: An overview of disability employment disclosures among UK firms. Corporate Social Responsibility and Environmental Management. 2019;26(1):170-85.

44.	Declercq J. It's Not Written All Over My Face: Constructing Chronic Pain as Invisible in Pain Clinic Consultations and Interviews. Clin J Pain. 2025;41(3).

45.	Blake H, Robinson DV, Chaplin WJ, editors. Views and experiences of employers towards workplace support for people with chronic pain; a mixed methods study to inform line manager training. 17th International Conference on Education and New Learning Technologies; 2025 30 June-2 July, 2025; Palma, Spain.

46.	Ysasi NA, Becton AB, Chen RK. Stigmatizing effects of visible versus invisible disabilities. 2018.

47.	Javaid SF, Yusuf R. Invisible disabilities. The Palgrave Encyclopedia of Disability: Springer; 2024. p. 1-7.

48.	Monsivais DB. Decreasing the stigma burden of chronic pain. Journal of the American Association of Nurse Practitioners. 2013;25(10):551-6.

49.	Fine PG. Long-Term Consequences of Chronic Pain: Mounting Evidence for Pain as a Neurological Disease and Parallels with Other Chronic Disease States. Pain Medicine. 2011;12(7):996-1004.

50.	Grant M, Rees S, Underwood M, Froud R. Obstacles to returning to work with chronic pain: in-depth interviews with people who are off work due to chronic pain and employers. BMC Musculoskeletal Disorders. 2019;20(1):486.

51.	Harwood R. Work and industrial relations in an age of austerity: disability, reasonable adjustments, and austerity. 2011.

52.	Cunningham I, James P, Dibben P. Bridging the Gap between Rhetoric and Reality: Line Managers and the Protection of Job Security for Ill Workers in the Modern Workplace. British Journal of Management. 2004;15(3):273-90.

53.	Blanco-Oliver A, Veronesi G, Kirkpatrick I. Board heterogeneity and organisational performance: The mediating effects of line managers and staff satisfaction. Journal of Business Ethics. 2018;152(2):393-407.

54.	Townsend K, Dundon T. Understanding the role of line managers in employment relations in the modern organisation. Employee relations. 2015;37(4):1-10.

55.	Blake H, Chaplin WJ, Gupta A. The effectiveness of digital interventions for self-management of chronic pain in employment settings: a systematic review. British medical bulletin. 2024;151(1):36-48.

**Supplementary files**

**S1 Table: COREQ**

**S2 Table: 15 Point Checklist for Thematic Analysis**

**S1 Text: Interview Guide for Intervention Group Stakeholders**

**S2 Text: Interview Guide for Control Group Stakeholders**
