# Supplementary material for: “Shining a light on chronic pain”: A qualitative study of stakeholder views towards chronic pain at work and the Pain-at-Work Toolkit
Source: PLoS One. 2026 Jul 2;21(7):e0351938. doi: 10.1371/journal.pone.0351938 (PMC13327183; doi:10.1371/journal.pone.0351938)
Supplement: Attachment — Submitted filename: Response to reviewers.docx [file pone.0351938.s008.docx]

27/04/2026

Dear Editors,

We would like to thank you and the reviewer for your careful evaluation of our manuscript and for the constructive and insightful comments provided. We have revised the manuscript in response to all points raised and believe these changes have strengthened the clarity and quality of the paper.

Below, we provide a point-by-point response to each comment. Reviewer comments are shown in italics, followed by our responses in regular text. All changes in the manuscript are highlighted/tracked. The line numbers specified refer to the clean version of the manuscript rather than the one with tracked changes in red.

Please note that we have added a Data Availability Statement after Keywords and before Introduction and that the Discussion has been substantially revised in line with reviewer comments.

**Comment 1(Introduction):**

While there is a clear need to better support people with chronic pain at work, a recent survey…*Consider paraphrasing?*

**Response**
Thank you for this suggestion. We have revised this sentence to improve clarity and reduce similarity with the original source.

**Change made (Introduction, line,94)**
“The revised sentence now reads: There is increasing recognition of the need to better support people with chronic pain in the workplace.

**Comment 2 (Introduction)**

However existing tools do not tend to focus specifically on workplace strategies or provide in depth resources. *Can you elaborate? Are there some that do?*

**Response**

We thank the reviewer for questioning this. To our knowledge the Pain-at-Work Toolkit is novel but we have clarified its positioning.

**Change made (Introduction, lines 104-105 inclusive)**

However existing tools digital tools rarely focus specifically on workplace self-management strategies and where work-related content is included it is typically limited and lacks in depth resources.

**Comment 3 (Introduction)**

The Pain-at-Work Toolkit was developed to address this gap. *Is there a gap or is this "a tool" for this particular purpose?*

**Response**

We agree that the Pain-at-Work Toolkit is a tool designed for this purpose; however, it was developed specifically in response to a gap in existing digital interventions, which rarely address workplace self-management of chronic pain. We have clarified this in the manuscript:

**Change made (Introduction, lines 105-107 inclusive)**

The Pain-at-Work Toolkit was developed to address this identified gap by providing a digital resource specifically designed to support workplace self-management of chronic pain.

**Comment 4 (Introduction)**

This digital toolkit was co-created with healthcare professionals, employers and people with chronic pain… *Not line managers or managers? If so why not?*

**Response**

We thank the reviewer for seeking clarification. The cited study (Blake et al., 2022) used the umbrella term “employers” to encompass a range of organisational roles and responsibilities. This included line managers, as well as middle managers/team leaders and senior managers, all of whom were represented in both the initial stakeholder consultation and the subsequent employer survey. We have clarified this interpretation in our response but retained the original wording in the manuscript for consistency with the cited study.

**Comment 5 (Introduction)**

…aiming to provide a comprehensive resource…*did you aim at producing or test the feasibility of the Pain-at-Work Toolkit? Or both?*

**Response**

We thank the reviewer for this comment. The statement referring to the toolkit as a “comprehensive resource” relates specifically to its development, rather than the feasibility study. The aim at this stage was to produce a resource to support individuals in self-managing chronic pain at work. The feasibility study, described separately in the manuscript, had a distinct aim: to assess the feasibility, acceptability, usability, and safety of evaluating the toolkit ahead of a definitive trial.

**Comment 6 (Introduction)**

An open-label, 2-arm multicentre pragmatic cluster-randomised feasibility trial of the Pain-at-Work Toolkit. This compared an intervention group with ‘treatment as usual’ controls in working adults with chronic pain in England [24]. This is ongoing and will be reported separately. *Is this relevant to the current paper? I find it more distracting than relevant.*

**Response**

We agree and have deleted accordingly.

**Change made (Introduction)**

**Comment 7 (Introduction)**
…feasibility trial of the Pain-at-Work Toolkit. *reference to published study protocol or online repository?*

**Response**

We agree and have cited the published study protocol.

**Change made (Introduction, lines 107)**

The Pain-at-Work Toolkit was developed to address this identified gap by providing a digital resource specifically designed to support workplace self-management of chronic pain[24].

**Comment 8 (Introduction)**

The study aimed to explore how individuals with chronic pain are supported in the workplace from the perspective of organisational stakeholders…*This study?*

**Response**

We thank the reviewer for highlighting this lack of clarity. We have removed this text to avoid ambiguity and to reduce repetition of the research objectives.

**Change made (Introduction)**

**Comment 9 (Methods, Study design)**

This was a qualitative interview study, with individual semi-structured interviews. Reporting aligns with the consolidated criteria for reporting qualitative research (COREQ) [25] (S1). Since the aim was to explore the individual stakeholder’s views rather than gain a global perspective [26]; semi-structured interviews were conducted. This approach allowed the discussion to be focused on key topics while also allowing the researcher the flexibility and autonomy to explore relevant topics if and when they were raised [27]. *I feel like you are repeating yourself somewhat in this section.*

**Response**

We thank the reviewer for this helpful observation. To reduce repetition, we have removed overlapping study details from the Introduction and streamlined the description of the qualitative approach in the Methods section.

**Comment 10 (Methods, Recruitment and Interview Schedule)**

All but two of the interviews were audio and video recorded. *How did you store the data and were there any personal data collected that may interfere with GDPR-regulations?*

**Response**

We thank the reviewer for this important point. The study involved the collection of qualitative interview data, which included personal data. All data were handled in accordance with the General Data Protection Regulation. Participants provided informed consent prior to participation, and all data were pseudonymised during analysis and anonymised for reporting. Data were stored securely in line with institutional data protection policies.

**Change made (Methods, lines 139-141 inclusive)**

Study forms were held securely in locked cabinets and access was limited to authorised personnel. All data were held on secure password protected servers with restricted access.

**Comment 11 (Methods, Recruitment and Interview Schedule)**

Interviews lasted between 30 and 58 minutes with a mean of 44 minutes. *To me this is a result?*

**Response**

We agree that this information is more appropriately reported as a result. We have therefore moved the description of interview duration to the Results section under stakeholder characteristics.

**Change made (Results, lines 253-254 inclusive)**

**Comment 12 (Results, Stakeholder Characteristics)**

The target population was individuals (stakeholders) employed in a role providing management or support for employees… *Maybe the answer to my earlier question about participants? Consider aligning.*

**Response**

We thank the reviewer for this enquiry. The earlier query relates to participants in the feasibility trial, whereas this section refers to a separate qualitative study involving organisational stakeholders, conducted for different purposes and with a distinct target population. We have more clearly differentiated the two studies where mentioned

**Comment 13 (Results, Stakeholder Characteristics)**

…eligible organisation participating in the feasibility trial of the Pain-at-Work Toolkit [24]. Eligible organisations were defined as organisations located in England, from any sector (public, private or third) with 10 or more employees…*Why this number (10)?*

**Response**

We thank the reviewer for this requesting clarification. The threshold of ≥10 employees was selected in the feasibility trial - small organizations were excluded because very small workforces are less likely to employ multiple employees with chronic pain, limiting recruitment feasibility. We have not added this explanation to the current manuscript as this detail will be published in the broader feasibility study paper.

**Comment 14 (Research Questions/RQs)**

RQ3: How do organisations perceive the Pain-at-Work Toolkit as a mechanism for supporting people with chronic pain at work? *(how) were the participants informed about or instructed in the use of the toolkit?*

**Response**

We thank the reviewer for this helpful question. We have now clarified how participants were introduced to the Pain-at-Work Toolkit in the section entitled Overview of Stakeholders’ Responses to the Pain-at-Work Toolkit. This information relates specifically to a subset of participants (n=8) from organisations in the intervention arm of the feasibility trial, who were asked to reflect on the implementation of the toolkit within their organisation. As this formed a relatively small and specific component of the study, it has been described in the Results section rather than in the main Methods section.

**Change made (Overview of Stakeholders’ Responses to the Pain-at-Work Toolkit, lines 899-905 inclusive)**

As part of the feasibility trial, stakeholders had previously been involved in promoting the toolkit to employees. To support reflection during the interviews, stakeholders were provided with access to the toolkit and were invited to briefly remind themselves of its content prior to discussing their experiences and perceptions.

**Comment 15 (Research Questions)**

Stakeholders were also asked to estimate the number of employees in the organisation and the percentage of workers with chronic pain in their organisation at recruitment into the trial and then at interview. *Why? Is this relevant to the aim of the study? Or was there a secondary/tertiary aim?*

**Response**

We thank the reviewer for this important question. These data were collected to provide contextual insight into organisational stakeholders’ perceptions of the prevalence of chronic pain within their workforce. While not a primary aim, this information helped to inform interpretation of the qualitative findings. Specifically, as reported in Theme 1 (“Not all disabilities are visible”), several stakeholders were unable to estimate the proportion of employees with chronic pain, while others provided only broad approximations. This finding reinforces the perception of chronic pain as an often unseen and poorly recognised condition in the workplace. To improve clarity, we have clarified the purpose of collecting these data in the manuscript.

**Change made (Research Questions, lines 180-183 inclusive)**

Stakeholders were also asked to estimate the number of employees in the organisation and the percentage of workers with chronic pain in their organisation at recruitment into the trial and then at interview. These data were collected to provide contextual insight into organisational stakeholders’ perceptions of the prevalence of chronic pain within their workforce.

**Comments 16, 17, 18, 19, 20, 21, 22, 23, 24, 25 (Analysis)**

**Comment 16**

The first step involved the researcher (AT) familiarising themselves with the data and since the same researcher (AT) interviewed stakeholders, checked transcripts, and collaboratively conducted analysis, they retained a close connection. *Only one researcher? Normally two or more would be involved in this phase.*

**Comment 17**

How did beliefs and experiences of AT influence this phase? *Did you (can you) elaborate on "where you come from" and how your (the teams') beliefs and experiences impacts/synthesises with the analysis and results?*

**Comment 18**

These generated preliminary themes which reflected patterns of shared meaning across interviews. *along with those of AT, I assume?*

**Comment 19**

These overarching themes were discussed and reviewed with two other researchers (HB and EW). *did they not familiarise themselves with the raw data (transcripts)? Or did they compare the preliminary themes to their own experiences and beliefs?*

**Comment 20**

These themes were then further defined… *with what / which underlying focus? I find it both necessary and highly interesting to know the impact of the researchers in this process.*

**Comment 21**

alone anchored by a unifying concept… *according to what standards or ideals? Did you have a specific theory in mind?*

**Comment 22**

Analysis was conducted manually in Microsoft Word rather than using analysis software like NVivo. *Since you have not informed the reader about all your "not choices", I suggest you delete this.*

**Comment 23**

This decision was influenced by the researcher’s personal preference [33] and a desire to ensure the context of the qualitative data was retained. *Is there any particular reason why this is included? I - personally - believe that NVivo makes the process easier and more structured, but it's by no means the "right way" to do it. However, I would suggest that you discuss if/how this can have impacted your results in the limitations section. Particularly, in the light of only having one researcher doing the initial analysis.*

**Comment 24**

Reflexive thematic analysis places the researcher at the heart of the process rather than considering their subjectivity as bias or a lack of validity [28] and hence it is important to articulate the role of the researchers in the team. *thank you -) Maybe move this section up?*

**Comment 25**

HB is a behavioural scientist with 30 years of experience in public health and applied health research. WJC and EW have a background in health psychology and a particular research interest in work and health, including chronic pain and work. AT has a background in psychology and over 35 years’ experience as an industry researcher specialising in healthcare. *Please elaborate a bit about how you see the topic, e.g., what are your preconceptions about the role of the managers, and what are you motivation to look into this particular topic (workplace interventions as a mean to support people with pain to remain at work)? This is likely to inform and influence your themes.*

**Response to comments 16 to 25 inclusive**

We thank the reviewer for these thoughtful and related comments regarding researcher involvement, analytic approach, reflexivity, and methodological transparency. We agree that transparency regarding analytic rigour is important, particularly given a single researcher led the analysis. In response, we have substantially revised the Analysis section to provide a more detailed account of the analytic process, including the role of reflexivity and the contribution of the wider research team in reviewing and refining codes and themes. Three researchers were involved and we have clarified this. While initial coding was conducted by one researcher (AT), developing interpretations were discussed and critically reviewed with other members of the research team (HB and EW), who contributed additional disciplinary perspectives and supported the refinement of themes. We have also expanded the reflexivity section to more explicitly describe how the researchers’ backgrounds, perspectives, and motivations informed the analytic process, in line with the principles of reflexive thematic analysis, where researcher subjectivity is understood as an integral component of knowledge production rather than a source of bias to be eliminated. We have removed the previous reference to software choice, as we consider this to reflect analytic approach rather than a determinant of methodological quality. While qualitative data analysis software such as NVivo can support data management and organisation, its use is not required for rigorous analysis, particularly within a reflexive thematic analysis framework. Given the expanded detail now provided on analytic procedures and reflexivity, we have not included software use as a limitation.

These revisions strengthen the transparency, coherence, and methodological rigour of the analytic approach.

**Change made (Analysis, lines 184-208 inclusive)**

**Comments 26 and 27 (Information Power)**

In this instance, a larger purposive sample of 15 reflects the scope of the research questions and the specificity of the stakeholders who all hold pertinent roles looking after the health and wellbeing of the workforce. *Did you know this when you were planning or did you stop looking for participants when you felt that you had reached data saturation? Either way, how did you end up with 15 participants?*

On balance, we concluded that the qualitative sample of 15 had sufficient information power. *This sounds quite pragmatic. Is that true?*

**Response**

We thank the reviewer for requesting confirmation. The sample size was not determined a priori using a fixed numerical target but was guided by principles of purposive sampling and information power. Participants were recruited from eligible organisations until the available pool of stakeholders had been approached and responses had been maximised. In practice, 15 stakeholders were recruited from a total of 30 invited participants. Recruitment ceased at this point as no further participants were available within the sampling frame. During analysis, the research team observed that sufficient depth and richness of data had been achieved to address the study aims, and that no substantially new themes were emerging, indicating that the sample held adequate information power.

We acknowledge that this process was necessarily pragmatic, but it was guided by the principles of information power, including the specificity of the sample, the focused study aim, the quality of dialogue, and the analytic approach. We have clarified this process in the manuscript to improve transparency.

**Comment 28 (Results)**

Results Depending on the topic, results either refer to organisations (n=12) or stakeholders (n=15). I *suppose this is the same data but analysed differently? Please elaborate in the methods section.*

**Response**

We thank the reviewer for this observation. The data derive from a single set of interviews with organisational stakeholders (n=15), representing 12 organisations. We have clarified this distinction in the Results section and added to the Methods section to improve transparency.

**Change made (Methods lines 152-154 inclusive and Results lines, 231-232 inclusive)**

**Methods**

Interviews were conducted with organisational stakeholders (n=15), representing 12 organisations. Analysis was conducted at both the individual (stakeholder) level and, where appropriate, at the organisational level, to reflect shared or organisation-specific perspectives.

**Results**

Depending on the focus of analysis, findings are reported at either the individual (stakeholder, n=15) or organisational level (n=12 organisations represented)).

**Comment 30 (Results)**

Please note the final section summarising stakeholders’ responses relating to the Pain-at-Work Toolkit includes only the views of stakeholders from organisations that were allocated to the intervention group in the trial (n= seven organisations, eight stakeholders) since employees in the control group did not receive access to the toolkit. The data source is stated throughout. *Consider elaborating in methods*

**Response**

We thank the reviewer for this helpful suggestion. We have now clarified in the Methods section that only stakeholders from organisations allocated to the intervention group (n=7 organisations; n=8 stakeholders) were asked questions relating to the Pain-at-Work Toolkit (RQ3), as participants in the control group did not receive access to the toolkit.

**Change made (Methods lines 154-161)**

Only stakeholders from organisations allocated to the intervention group (n=7 organisations; n=8 stakeholders) were asked questions relating to the Pain-at-Work Toolkit (RQ3). The Pain-at-Work Toolkit is a digital resource designed to support employees in the self-management of chronic pain in the workplace. It comprises five sections covering understanding chronic pain, chronic pain and disability, information on workplace adjustments and support, self-management strategies and links to additional resources. The toolkit was made available to employees within organisations randomised to the intervention arm of the feasibility trial.

**Comment 31 (Results, Organisational Characteristics)**

Twelve organisations were recruited for this study, eleven of which were classified as large organisations, i.e. with >250 employees [37]. Seven organisations were cluster-randomised to the intervention arm of the trial and five to the control group. *in this study or another?*

**Response**

We thank the reviewer for highlighting this lack of clarity. The organisations referred to here were recruited as part of the Pain-at-Work feasibility trial, from which stakeholders for this qualitative study were subsequently sampled. At the time of stakeholder recruitment, seven organisations had been randomised to the intervention arm (with access to the Pain-at-Work Toolkit) and five to the control arm. We have clarified this in the manuscript to distinguish between the feasibility trial and the qualitative study.

**Change made (Results, Organisational Characteristics, lines 239-241 inclusive)**

Twelve organisations from the Pain-at-Work feasibility trial were included, from which stakeholders for this qualitative study were recruited. Eleven organisations were classified as large i.e. with >250 employees[28]. At the time of recruitment, seven organisations had been randomised to the intervention arm of the feasibility trial (with access to the Pain-at-Work Toolkit) and five to the control arm.

**Comment 32: (Thematic Analysis)**

Figure 1: Themes *- I would like to see the initial themes and how they were transformed into the final themes (e.g., in a flow diagram?). This helps the reader understand what the initial concepts were, and how they fitted (or didn't) into the final themes. In my experience, initial themes do not fit perfectly, and the learning in this phase seems to reflect some of the values the team adds to the results.*

**Response**

We thank the reviewer for this helpful and constructive suggestion. We agree that illustrating the development of themes enhances transparency in the analytic process. We have now included a flow diagram (Supplementary files: S5) to illustrate how initial codes and preliminary themes were iteratively developed and refined into the final themes.

**Change made**

(Supplementary files – added S5: Flowchart of Thematic Analysis)

**Comment 33 (Results, Thematic Analysis)**

Theme 1: Not All Disabilities Are Visible. *Can you make a description (definition) of subthemes that match this theme to clearly differentiate them from subthemes that do not?*

**Response**

We have now added brief descriptions of each subtheme within the Results section to clarify how they relate to their respective overarching themes and how they are conceptually distinct. For example, within Theme 1, “Policies and Provisions” refers to formal organisational structures and resources available to support employees with chronic pain, whereas “Organisational Support” captures the broader cultural and practical support experienced within the workplace. These additions aim to provide clearer differentiation between subthemes and strengthen the interpretation of the findings.

**Change made (Results, lines inclusive)**

Theme 1: lines 285-295 inclusive

Theme 2: lines 648-654 inclusive)

Theme 3: lines 811-824 inclusive

**Comment 34 (Results, Theme 1a Policies and Provisions)**

*if somebody is talking to you and having that conversation saying that the organisation treats that as something that's important and needs support”.* [ID15, Occupational Health & Wellbeing]. *Do the IDs represent organisations or their personal experiences here/in general?*

Response

The participant IDs refer to individual stakeholders. The views expressed reflect their perspectives based on their professional role and experience within their organisation, including both personal insights and their understanding of organisational practices. We have clarified this in the manuscript.

**Change made (Results, Theme 1a Policies and Provisions, lines 277-279 inclusive)**

Participant IDs refer to individual stakeholders and are used to anonymise quotations; the views expressed reflect their perspectives based on their professional role within their organisation, rather than representing formal organisational positions.

**Comment 35 (Results, Theme 1, Table 4, Policies and Provisions)**

Table 4 - Support Services - *are these sub-sub-themes??*

**Response**

These are not intended to represent an additional level of analysis (i.e., sub-sub-themes). The table provides illustrative examples of organisational policies and provisions reported by stakeholders within the subtheme *Policies and Provisions*, rather than constituting further thematic categories.

**Comment 36 (Results, Theme 1, Table 4, Policies and Provisions)**

Table 4 - Support Services **-** *is this a headline or a result?*

**Response**

“Support Services” is intended as a category heading within Table 4, under which specific types of support services (e.g., Occupational Health, Health and Wellbeing, Physiotherapy) are listed. It does not represent a standalone result.

**Comment 37 (Results, Theme 1a, Policies and Provisions, Theme 1a Specific Policies for Chronic Pain)**

Chronic pain was very much perceived to be a ‘hidden’ or ‘invisible disability’ which had implications for the support that employees received. *at a personal level, i.e.., who has pain, or at the organisational level, i.e., we don't really look for it?*

**Response**

We thank the reviewer for this helpful comment. The perception of chronic pain as a “hidden” or “invisible” condition operates at both the individual and organisational levels. At the individual level, stakeholders described how employees may not disclose their condition, making it less visible. At the organisational level, support is often contingent on disclosure, meaning that organisations may not actively identify or record cases of chronic pain unless it is raised by the employee.

We have clarified this in the manuscript to better reflect these interconnected perspectives.

**Change made (Results, Theme 1a Policies and Provisions, Theme 1a Specific Policies for Chronic Pain lines 363-366 inclusive)**

Chronic pain was widely perceived as a “hidden” or “invisible” condition, both because employees may not disclose their experiences and because organisational support is often contingent on such disclosure, with implications for the support that employees receive

**Comment 38 (Results, Theme 1a Policies and Provisions, Theme 1a Specific Policies for Chronic Pain)**

This was reinforced by the finding that seven of the 15 stakeholders had no idea of the proportion of employees with chronic pain, and the remaining eight could at best guess at anything from occasional cases to a high proportion of the workforce. *Are you using this question to "triangulate", i.e., support the interview data?*

**Response**

This information was used to provide contextual support for the qualitative findings rather than formal triangulation. Specifically, it offers additional insight into stakeholders’ awareness of chronic pain within their organisations, which aligns with and reinforces the theme of chronic pain as an often unseen or unrecognised condition.

We have clarified this in the manuscript.

**Change made (Results, Theme 1a Policies and Provisions, Theme 1a Specific Policies for Chronic Pain, lines 334-337 inclusive)**

This interpretation was further supported by the finding that seven of the 15 stakeholders had no idea of the proportion of employees with chronic pain, and the remaining eight could at best guess at anything from occasional cases to a high proportion of the workforce.

**Comment 39 (Results, Theme 1a Policies and Provisions, Theme 1a Specific Policies for Chronic Pain,)**

Stakeholders acknowledged that invisible disabilities, such as chronic pain, which was often compared to mental health conditions, were poorly understood, could be stigmatised and received less support than visible disabilities like physical or sensory impairments. *Does signal that they are aware of the extent and impact on works lives?*

**Response**

We thank the reviewer for this important question. The data suggest that stakeholders were aware of the challenges associated with invisible disabilities, including stigma and lack of understanding, rather than demonstrating a detailed awareness of their prevalence or impact on working lives. As highlighted elsewhere in the manuscript, stakeholders often reported limited knowledge of the extent of chronic pain within their organisations, reflecting reliance on employee disclosure and limitations in organisational data capture. We have clarified this distinction in the manuscript.

**Change made (Results, Theme 1a Policies and Provisions, Theme 1a Specific Policies for Chronic Pain, lines 345-351 inclusive)**

Stakeholders acknowledged that invisible disabilities, such as chronic pain, (often compared to mental health conditions), were poorly understood and could be stigmatised for example through scepticism about the legitimacy of chronic pain and concerns about being judged or not taken seriously by employers, managers, or colleagues and received less support than visible disabilities like physical or sensory impairments. This reflects an awareness of the challenges associated with invisible conditions, rather than a detailed understanding of the prevalence of chronic pain and its impact within the workplace.

**Comment 39 (Results, Theme 1a Policies and Provisions, Theme 1a Specific Policies for Chronic Pain)**

could be stigmatised and received less support than visible disabilities like physical or sensory impairments. *in what way, and how does that relate to the toolkit?*

**Response**

We thank the reviewer for requesting clarification and context. Stakeholders described stigma in terms of negative perceptions and attitudes from employers, line managers, and colleagues, including scepticism about the legitimacy of chronic pain and concerns about being judged or not taken seriously.

This finding relates to broader perceptions of invisible conditions within the workplace rather than directly to the Pain-at-Work Toolkit. We have clarified the nature of stigma in the manuscript.

**Change made (Results, Theme 1a Policies and Provisions, Theme 1a Specific Policies for Chronic Pain, lines 345-349 inclusive)**

Stakeholders acknowledged that invisible disabilities, such as chronic pain, (often compared to mental health conditions), were poorly understood and could be stigmatised for example through scepticism about the legitimacy of chronic pain and concerns about being judged or not taken seriously by employers, managers, or colleagues and received less support than visible disabilities like physical or sensory impairments.

**Comment 40 (Results, Theme 1a Policies and Provisions, Theme 1a Specific Policies for Chronic Pain)**

“Stigma around some people with conditions like fibro(myalgia) is still quite apparent, and that's even within healthcare, never mind a manager or an HR professional.” [ID11, MSK & OH Physiotherapist]

*and manager?? or employee?*

**Response**

We thank the reviewer for this point. The quotation is from an organisational stakeholder working as a musculoskeletal (MSK) and occupational health physiotherapist who is discussing their view on the perception of other staff members such as a manager and HR professional.

**Comment 41 (Results, Theme 1a Policies and Provisions, Theme 1a Specific Policies for Chronic Pain)**

“If you can't see it, it isn't a broken leg. You haven't got a bad back; you know that sort of thing, or you’re not in a wheelchair…I really don't understand what the issue here is. And it's a bit like mental health. You know the two go hand in hand.” [ID07, Head of Wellbeing] *does show a fairly good understanding of the problem, right?*

**Response**

We thank the reviewer for this thoughtful question. The quotation reflects an awareness of the challenges associated with invisible conditions, including the comparison to mental health. However, it also highlights ongoing misunderstanding and scepticism around chronic pain, particularly in how it is perceived relative to more visible conditions.

**Comment 41 (Results, Theme 1a Policies and Provisions, Theme 1a Specific Policies for Chronic Pain – Reasonable Adjustments)**

“So, if it was about if it was about chronic pain it would depend on what kind of problem at work that was. But if it was about difficulty sitting for long periods of time then you might be directed to have an enhanced DSE assessment with the health and safety team.” [ID01, Head of Workforce Policy and Wellbeing] *??*

**Response**

We thank the reviewer for questioning this. This quotation is included to illustrate how stakeholders described the practical application of reasonable adjustments, which were often framed in relation to specific functional difficulties (e.g., sitting for prolonged periods) rather than chronic pain as a condition.

We have clarified the purpose of this quotation and repositioned in the manuscript to better highlight how adjustments are operationalised in practice.

**Change made (Results, Theme 1a Policies and Provisions, Theme 1a Specific Policies for Chronic Pain – Reasonable Adjustments, lines 370-379 inclusive)**

Stakeholders often described reasonable adjustments in relation to specific functional limitations rather than chronic pain as a condition, for example:

“So, if it was about if it was about chronic pain it would depend on what kind of problem at work that was. But if it was about difficulty sitting for long periods of time then you might be directed to have an enhanced DSE assessment with the health and safety team.” [ID01, Head of Workforce Policy and Wellbeing]

“So, we do the DSE assessments, you know, so that's about your screen, your keyboard, your mouse, your chair, things like this.” [ID12, Lead Research Nurse]

**Comment 42 (Results, Theme 1a Policies and Provisions, Theme 1a Specific Policies for Chronic Pain – Reasonable Adjustments)**

So, we do the DSE assessments, you know, so that's about your screen, your keyboard, your mouse, your chair, things like this.” [ID12, Lead Research Nurse] *where do the HCPs fit into the interview inclusion criteria? Are they company-based?*

**Response**

We thank the reviewer for requesting clarification on the sample. This participant was included as an organisational stakeholder, as they held a role within the organisation that involved responsibility for employee health and wellbeing. In this case, the Lead Research Nurse was employed within the organisation and contributed to workplace wellbeing initiatives, as well as being involved in decisions regarding organisational participation in research.

**Comment 43 (Results, Theme 1a Policies and Provisions, Theme 1a Specific Policies for Chronic Pain – Reasonable Adjustments)**

“So, if I give you an example of the of a tailored work plan for somebody with back pain. So, what we might do is we introduce rest breaks to move around, which is sort of 5 minutes or 10 minutes in between each patient. *I think it would be very useful with a table showing the kind of organisations you have included. One would suppose that a university or hospital (maybe even those occupied with work-related or pain-related research) may be more prone to get involved but will also bias the results? Similarly, an overview of the roles and education of the participants may shed some light on this as well?*

**Response**

We thank the reviewer for this helpful suggestion. We agree that additional contextual information regarding organisations and stakeholder roles is valuable. To minimise the risk of identification in a small qualitative sample, organisational characteristics and data on stakeholders’ roles are presented in aggregated form in additional tables in the Results section. Information on educational background was not systematically collected; however, stakeholder roles provide an indication of relevant professional expertise.

**Change made (Results, Table 2: Stakeholder Roles, starts line 263)**

**Comment 44 (Results, Theme 1a Policies and Provisions, Theme 1a Specific Policies for Chronic Pain – Reasonable Adjustments)**

“But, you know, reasonable adjustments are, gosh, they're just so varied, aren't they?” [ID12, Lead Research Nurse]

“Myself and several colleagues all have lower back pain after childbirth. We are all very different but we all got a blanket set of advice.” [ID08, Senior Clinical Research Practitioner]

*these are contrasting views, right*

**Response**

Thank you for this comment. These data are not intended to represent contrasting views; rather, they reflect stakeholder recognition of the need for individualised adjustments alongside examples where this was not consistently reflected in practice. We have revised the illustrative quote to better capture this point.

**Change made (Results, Theme 1a Policies and Provisions, Theme 1a Specific Policies for Chronic Pain – Reasonable Adjustments, lines 408-411)**

“But, you know, reasonable adjustments are, gosh, they're just so varied, aren't they? It's about how long can you sit? How does that affect you? … this is why I say it's a really individual basis because you can't put a blanket set of questions on one person. And if you do, all you do is make it a process.” [ID12, Lead Research Nurse]

**Comment 45 (Results, Theme 1a Policies and Provisions, Theme 1a Specific Policies for Chronic Pain – Reasonable Adjustments)**

*I think it would be very useful with a summary statement at the end of each subtheme that summarises the main points/viewpoints and to what extent your participants seem to agree.*

**Response**

We agree that clearer summary statements would strengthen the presentation of findings and have added brief concluding sentences to each subtheme to summarise the key points and indicate the level of consistency in stakeholder perspectives.

**Change made (Results for each subtheme)**

**Comment 46 (Results, Theme 1a Policies and Provisions, Theme 1a Specific Policies for Chronic Pain – Reasonable Adjustments)**

In one case, a desire to accommodate the specific needs of workers with chronic pain was driven by the values and lived experience of one of the Directors. *No quote on this?*

**Response**

We have revised the text to clarify that this reflects an interpretation of stakeholder accounts rather than a direct quotation.

**Change made (Results, Theme 1a Policies and Provisions, Theme 1a Specific Policies for Chronic Pain – Reasonable Adjustments, lines 415-416)**

In one case, this appeared to be influenced by the values and lived experience of a senior leader.

**Comment 47 (Results, Theme 1a Policies and Provisions, Theme 1a Specific Policies for Chronic Pain – Reasonable Adjustments)**

However, among stakeholders, this was the exception rather than the norm, with most organisations relying on standardised solutions. *I feel like I'm missing some data here. Can you provide all the transcripts (anonymised) in the appendix?*

**Response**

Thank you for this suggestion. While we recognise the value of transparency in qualitative research, full interview transcripts have not been included as an appendix due to the risk of participant re-identification, particularly given the small qualitative sample and inclusion of detailed workplace and role-specific information. Participants did not provide explicit consent for full transcript sharing. In line with qualitative research best practice, we have included illustrative anonymised quotations within the manuscript to support the analysis.

**Comment 48 (Results, Theme 1a Policies and Provisions, Theme 1a Specific Policies for Chronic Pain – Sickness Absence)** *I miss some results (quotes) in this section*

**Response**

Thank you for this comment. We have reviewed this section and clarified the alignment between the narrative and supporting data. Additional linking between the text and illustrative quotes has been included to ensure that key points are clearly grounded in stakeholder accounts.

**Change made (Results, Theme 1a Policies and Provisions, Theme 1a Specific Policies for Chronic Pain – Sickness Absence)**

**Comment 49 (Results, Theme 1a Policies and Provisions, Theme 1a Specific Policies for Chronic Pain – Sickness Absence)**

Stakeholders were aware that chronic pain could present as a symptom of multiple long-term health conditions, including long COVID, fibromyalgia and musculoskeletal conditions such as lower back pain and rheumatoid arthritis*. is this a summary of findings (as I have requested above)? I find it a bit difficult to detect whether you are summarising or describing (undisclosed) data?*

**Response**

This sentence summarises stakeholder accounts derived from the qualitative data. We have revised the text to make this clearer and to more explicitly signal that it reflects analysed findings rather than narrative description.

**Change made (Results, Theme 1a Policies and Provisions, Theme 1a Specific Policies for Chronic Pain – Sickness Absence, lines 448-450 inclusive)**

Analysis of stakeholder accounts indicated that chronic pain was sometimes associated with long-term health conditions, including long COVID, fibromyalgia and musculoskeletal conditions such as lower back

448-450

**Comment 50 (Results, Overview of Stakeholders’ Responses to the Pain-at-Work Toolkit)**

*I am missing an introduction to the toolkit (if this is part of the aim of the study). If this is not a part of the aim of the study, I suggest to delete it.*

**Response**

We have added a brief description of the Pain-at-Work Toolkit in the Methods section to provide context for this part of the analysis and clarified how stakeholders were introduced to the toolkit prior to providing feedback in the Overview of Stakeholders ‘Responses

**Change made (Methods lines 156-161)**

The Pain-at-Work Toolkit is a digital resource designed to support employees in the self-management of chronic pain in the workplace. It comprises five sections covering understanding chronic pain, chronic pain and disability, information on workplace adjustments and support, self-management strategies and links to additional resources. The toolkit was made available to employees within organisations randomised to the intervention arm of the feasibility trial.

**Change made (Overview of Stakeholders’ Responses to the Pain-at-Work Toolkit, lines 899-905 inclusive)**

As part of the feasibility trial, stakeholders had previously been involved in promoting the toolkit to employees. To support reflection during the interviews, stakeholders were provided with access to the toolkit and were invited to briefly remind themselves of its content prior to discussing their experiences and perceptions.

**Comment 51 (Overview of Stakeholders’ Responses to the Pain-at-Work Toolkit)**

Negative comments were sparse and focused on ease of navigation, for example, toolkit users didn’t always notice the ‘next’ arrows. *What are they?*

**Response**

We apologise for the lack of clarity We have expanded the section to place responses to the Pain-at-Work Toolkit into context.

**Change made (Overview of Stakeholders’ Responses to the Pain-at-Work Toolkit, lines 932-933 inclusive)**

Negative comments were sparse and focused on ease of navigation, for example, toolkit users didn’t always notice the arrows which were used to move forward and back through the five sections.

**Comment 51 (Overview of Stakeholders’ Responses to the Pain-at-Work Toolkit)**

Overall, the toolkit was said to be comprehensive… *How did they learn about it and what training did they receive in its use?*

**Response**

Thank you for requesting additional information. Stakeholders were provided with access to the toolkit as part of the study. No formal training was provided to stakeholders or to employees participating in the Pain-at-Work Feasibility Trial. This reflects the design of the toolkit as a standalone, accessible resource intended for independent use. Participants were, however, able to contact the trial researcher if they had any questions. The toolkit is structured so that users may work through it sequentially (from Sections 1 to 5), but each section also addresses a specific topic (e.g., workplace adjustments), enabling users to navigate the content in a self-directed way according to their needs.

**Comment 52 (Overview of Stakeholders’ Responses to the Pain-at-Work Toolkit)**

as well as the role of pain medications (rather than ‘painkillers’, which could raise false expectations) and their role in pain management. *pharmacotherapy or analgesics may be a more appropriate term? I agree that they are not "pain killers"*

**Response**

Thank you for this observation. We have revised the text to clarify that this reflects stakeholder accounts and to explicitly indicate that the terminology (‘painkillers’) was discussed within the data, with

as well as the role of pain medications (in pain management.

**Change made (Overview of Stakeholders’ Responses to the Pain-at-Work Toolkit, lines 927-929)**

stakeholders also debated the term ‘painkillers’, with some expressing concern that it could raise false expectations.

**Comment 53 (Overview of Stakeholders’ Responses to the Pain-at-Work Toolkit)**

we share stakeholder’s views on the Pain-at-Work Toolkit as one mechanism for addressing this gap in provisions. *Is this relevant?*

**Response**

We agree that this point is not directly relevant and have removed it from the manuscript.

**Change made (Overview of Stakeholders’ Responses to the Pain-at-Work Toolkit)**

**Comment 54 (Discussion)**

Overall, there was a lack of understanding of complex non-visible disabilities like chronic pain and insufficient resources to support an individual at work effectively. *I somehow feel that this is missing in the results (the complexity). The theme title seems to suggest it, but there are signs of contemporary understanding, while not really a lot of (quotes to suggest) signs of a lack of understanding? I'm particularly interested in if and how the background of the respondents and organisations may impact on this.*

**Response**

Thank you for this important observation. While some stakeholders demonstrated awareness of chronic pain as a complex and non-visible condition, this understanding was not consistent and was often accompanied by gaps in knowledge and confidence in supporting employees.

We have revised the Discussion to better reflect this nuance and to strengthen links to the Results, highlighting both areas of awareness and areas where understanding appeared limited. Exploration of differences by stakeholder background or organisational context was beyond the scope of the current analysis.

**Change made (Discussion**)

Substantially revised.

**Comment 55 (Discussion)**

An awareness of the gaps in organisational resources for chronic pain means that stakeholders welcomed The Pain-at-Work Toolkit as a comprehensive resource to support an employee in self-managing their condition in the workplace. *I don't see this well documented and wonder if it's out of scope for this paper? If not, I think it will require significantly more information about who and how*

**Response**

Thank you for this comment. This aspect is within the scope of the study (RQ3), which explores organisational perceptions of the Pain-at-Work Toolkit. We have clarified that stakeholders were not direct users of the toolkit but were asked to review and reflect on it. The text has been revised to more accurately reflect the nature of their engagement and the strength of their views.

**Change made (Discussion, lines 1036-1039 inclusive**)

Reflecting these identified gaps in organisational resources for chronic pain, stakeholders in organisations that were randomised to the intervention arm of the trial were overwhelmingly positive towards the Pain-at-Work Toolkit[24] perceiving it as a comprehensive resource to support employees in self-managing chronic pain at work.

**Comment 56 (Discussion)**

…we found that many organisations do not have disability workplace strategies and that employees with chronic pain are not specifically accommodated for within general policies for other long-term or chronic conditions [14, 41-43]. *Agree - this seems well-documented in the results*

**Response**

Thank you for this positive comment. We are pleased that this finding is clearly reflected in the results.

**Comment 57 (Discussion)**

chronic pain is hidden on many levels; its prevalence is hidden among sickness absence data, employee support is hidden within organisational policies and chronic pain is also often hidden by the employees themselves. *I see this but would not mind further elaboration (more quotes) and perhaps further analysis/thematic considerations*

**Response**

Thank you for this comment. We agree that this point benefits from further elaboration. We have revised the Discussion to more clearly integrate this interpretation across the themes and to strengthen the analytic links to the Results. The revised text highlights how chronic pain may remain hidden at multiple levels: within organisational systems (e.g., absence data), within policy structures, and through non-disclosure by employees.

**Change made (Discussion, lines 966-974 inclusive)**

Stakeholders also demonstrated that as a non-visible disability, chronic pain is hidden on many levels. Across the themes; its prevalence was described as obscured within organisational systems for example since sickness absence data did not capture chronic pain explicitly. Support for chronic pain was embedded within broader policies rather than specifically addressed[14, 39-41] and often inconsistently applied in practice. In addition, stakeholders highlighted that employees themselves may choose not to disclose chronic pain. Hence, while over one in four employees will experience chronic pain[48], stakeholder accounts in this study suggest that its true impact on both employee and organisations - particularly in terms of absenteeism, presenteeism and the individual’s mental and physical health is not fully recognised or systematically captured.

**Comment 58 (Discussion)**

…its true impact on both employee and organisations - particularly in terms of absenteeism, presenteeism and the individual’s mental and physical health is not fully recognised or systematically captured. *do you have any data on the role of the toolkit in this arena?*

Response

Thank you for this comment. Neither this stakeholder study not the feasibility trial assessed the impact (i.e., effectiveness) of the toolkit – this was not one of our study aims. Effectiveness and cost-effectiveness of the toolkit will be explored in a future study (definitive trial).

**Comment 59 (Discussion)**

This research confirms that large organisations commonly have a range of accommodations in place for long-term or chronic conditions. *the included organisations... I don't think the methods allow for extrapolation beyond this dataset?*

**Response**

Thank you for this comment. We agree that the original wording was overly broad. We have revised the text to clarify that this finding relates specifically to the organisations included in this study and does not imply wider generalisability.

**Change made (Discussion, lines 978-981 inclusive)**

This research also found that the predominantly large organisations included in the study commonly had a range of accommodations in place for long-term or chronic conditions, although not specifically for chronic pain.

**Comment 60 (Discussion)**

However, comparable with findings from prior research using an online survey and qualitative interviews [45], our research revealed that organisations tend to be ‘reactive’ rather than ‘proactive’ in dealing with chronic conditions in their workforce, only intervening when the employee was already taking frequent or extended sickness absence. *To me, this is not clear from the results section. Admittedly, there is soo much data that I might have missed a point. But I think that may be a point in itself.*

**Response**

Thank you for this comment. We agree that this interpretation required clearer signposting. We have revised the Discussion to more explicitly link this point to findings presented in Theme 1, where stakeholders described support as typically reactive and often initiated once sickness absence had become an issue.

The wording has also been refined to clarify that this reflects stakeholder accounts within this study.

**Change made (Discussion, lines 982-985 inclusive)**

However, consistent with prior research[43] findings from our stakeholder study indicated that organisations tend to be ‘reactive’ rather than ‘proactive’ in dealing with chronic conditions in their workforce, with support typically initiated when frequent or extended sickness absence was already any issue.

**Comment 61 (Discussion)**

Still, it is unlikely to allow a condition like chronic pain, which can become a separate condition, more complex than the pain caused by the original injury or disease [49] to be unpacked and effectively managed. *What does this mean/add to the sentence?*

**Response**

We agree that this sentence was unclear and have revised it to more clearly convey that high service demand may limit the effective assessment and management of chronic pain, particularly where it becomes a complex condition.

**Change made (Discussion, lines 985-987)**

Furthermore, access to specialist support services, such as occupational health, was often slow and provision limited, for example, to one-off consultations or short-term treatments which limited

the extent to which a complex condition like chronic pain could be effectively managed.

**Comment 62 (Discussion)**

In spite of its complexity stakeholders typically contextualised chronic pain as musculoskeletal…)

*but some didn't. I was surprised to find that this was the case, and I think you need to discuss this (perhaps reflect on who the respondents were)?*

Response

Thank you for this comment. We agree that there was variation in stakeholder perspectives and that this warrants further reflection. We have revised the text to acknowledge that not all stakeholders conceptualised chronic pain in this way, while noting that a predominantly musculoskeletal framing remained common. We also briefly consider the professional backgrounds of participants, noting that this framing was evident even among those with clinical experience.

**Change made (Discussion, lines 989-992 inclusive)**

Despite its complexity, stakeholders frequently contextualised chronic pain as musculoskeletal. Notably, this framing, was common even among participants with clinical backgrounds and reasonable adjustments at work tended to focus on the employee’s physical environment, such as an enhanced Display Screen Equipment (DSE) assessment.

**Comment 63 (Discussion)**

Although flexible working patterns were sometimes provided, specific provisions for chronic pain tailored to the individual employee’s requirements were rarely evident. *When were they used? And what did that mean to the organisation and/or managers? Did it make their job harder/easier? Did it have any impact on the culture (or did that culture of the organisation change first, e.g., the CEO who had personal experiences)?*

**Response**

Thank you for this comment. We have clarified that flexible working arrangements were typically implemented as part of reasonable adjustments. We have also elaborated on the implications for line managers and organisations, including the challenges of balancing operational demands with individual support needs. The impact of these adjustments on organisational culture was beyond the scope of this study and has been acknowledged as such.

**Change made (Discussion, lines 992-994 inclusive)**

Although flexible working patterns were sometimes provided, typically as part of reasonable adjustments, specific provisions for chronic pain tailored to the individual employee’s requirements… **Change made (Discussion, lines 996-1001 inclusive)**

While our stakeholders held organisational-level strategic and support roles, approximately a third also reported current or prior line management responsibilities. Both they and others recognised that managing employees who required significant and flexible workplace adjustments could be both time-intensive and, on occasion, emotionally draining. These individuals also spoke of the challenges in meeting organisational targets, for example, service delivery, while balancing the demands of reasonable adjustments for an individual with the workload and wellbeing of the broader team.

**Comment 64 (Discussion)**

While our stakeholders held organisational-level support roles, a third of them were also line managers themselves or had prior management experience. *Table... You shouldn't discuss anything that is not mentioned in the results, I think.*

**Response**

Thank you for this observation - we agree that all points interpreted in the Discussion should be clearly presented in the Results. The information regarding stakeholders’ line management experience was already reported in the Results (Stakeholder characteristics), where we noted that approximately one third of participants had current or prior line management responsibilities. However, we recognise that this detail may not have been sufficiently prominent.

**Change made (Results, Table 2, starts line 263)**

Added a row to the stakeholder characteristics table to explicitly report line management experience (current or prior), presented as an approximate proportion.

**Change made (Discussion, lines 263)**

Revised the wording in the Discussion to align more closely with the Results, clarifying that “approximately one third” of stakeholders reported current or previous line management responsibilities.

These changes improve the visibility and traceability of this point between the Results and Discussion sections.

**Comment 65 (Discussion)**

Line managers are typically the individual’s first point of contact when exploring reasonable adjustments in the workplace [14, 52]; and they also act as a ‘gatekeeper’ for accessing support [42, 53, 54]. Depending on their understanding and empathy for the impact of chronic pain on work, line managers can either help or hinder access to supportive services such as occupational health, described by employees in one study as the ‘line manager lottery’ [42]. *I don't see how this fits into the discussion. Maybe background if it matters for how or who you decided to include into the study?*

**Response**

Thank you for this comment. We have revised this section to more clearly link the discussion of line managers to our findings. Stakeholder accounts highlighted the central role of line managers in enabling or constraining access to workplace support, as well as variability in their knowledge and capability.

The revised text makes this connection more explicit and situates it in relation to existing literature.

**Change made (Discussion, lines 996-1009)**

While our stakeholders held organisational-level strategic and support roles, approximately a third also reported current or prior line management responsibilities. Both they and others recognised that managing employees who required significant and flexible workplace adjustments could be both time-intensive and, on occasion, emotionally draining. These individuals also spoke of the challenges in meeting organisational targets, for example, service delivery, while balancing the demands of reasonable adjustments for an individual with the workload and wellbeing of the broader team. However, stakeholders in the study readily acknowledged that line managers had a critical role in mediating organisational values and even in supportive organisations, local delivery of support to workers with chronic pain was not consistent. Stakeholder accounts highlighted the importance of line managers as an employee’s first point of contact when exploring reasonable adjustments in the workplace[14, 49] and as ‘gatekeepers’ for support[40, 50, 51]. Consistent with previous literature[47, 52], variation in managers’ understanding and empathy – particularly toward invisible disabilities such as chronic pain - was evident and influenced whether access to services such as occupational health was facilitated or hindered, sometimes described as a ‘line manager lottery’[40].

**Comment 66 (Discussion)**

line managers’ awareness, understanding and attitude towards chronic conditions such as pain varies considerably. *did you analyse/collect this from your cohort? If so, please report. If not; why not??*

**Response**

Thank you for this comment. This finding is supported by our data and is reported within Theme 2 (“Not all line managers are equal”), particularly within the subtheme “Individual variation,” where stakeholders described differences in line managers’ understanding, attitudes, and willingness to support employees.

We have revised the Discussion to make this link more explicit.

**Change made (Discussion, lines 1006-1009 inclusive)**

Consistent with previous literature[47, 52], variation in managers’ understanding and empathy – particularly toward invisible disabilities such as chronic pain - was evident and influenced whether access to services such as occupational health was facilitated or hindered, sometimes described as a ‘line manager lottery’[40].

**Comment 67 (Discussion)**

Stakeholders acknowledged that some line managers were less than sympathetic and supportive to hidden disabilities like chronic pain and did not understand the impact it could have on an individual’s life at home and at work. *So important - can this be thematised? I'd love to learn from your results, particularly what (subthemes) can identify or become targets for implementation of e.g., your toolkit*

**Response**

Thank you for this comment. We agree that this is an important finding. This issue is captured within Theme 2 (“Not all line managers are equal”), including the subthemes “Individual variation” and “Supporting line managers. “We have revised the Discussion to make this thematic structure more explicit and to highlight its relevance for identifying potential targets for implementation, including the need to better support and educate line managers.

**Change made (Discussion, lines 1011-1015 inclusive)**

As a result of this variability, three-quarters of stakeholders in our study identified line management as an area for improvement. This aligns with other studies that also identify a need for line manager training or resources[4, 5]. Stakeholders emphasised the importance of better supporting line managers through education about chronic pain and upskilling those with limited management experience who may find conversations with employees challenging

**Comment 68 (Discussion)**

Many spoke of the need to educate line managers about chronic pain and how to best support employees, as well as to upskill those with limited management experience. Great - can you show this (even more clearly) in your data/results?? What, how, when, who?

**Response**

We have revised the Results section to more explicitly present the detail underpinning this subtheme, including what types of support stakeholders identified, how these might be delivered, and who they were intended for. The Discussion has been amended accordingly.

**Change made Results, Theme 2: Not All Line Managers Are Equal, subtheme b: Supporting Line Managers, lines 770-782)**

During the discussion, some stakeholders spontaneously identified a need for additional resources to support line managers – including in one case a version of the Pain-at-Work employees toolkit and this concept was hence explored further. Given the variability of line manager support and the lack of organisational tools, stakeholders reacted favourably to the concept of a developing a Pain-at-Work Toolkit specifically for line managers hoping that a unified resource would educate line managers and help them better support their staff. Stakeholders suggested that such a resource should include key information (e.g. conditions that can cause chronic pain, and the symptoms an employee might present with), guidance on communication and engagement (e.g. how to have constructive conversations with employees and practical examples (e.g. of reasonable adjustments and case studies showcasing how other organisations have supported staff to continue working, as well as signposting to additional resources. Individuals suggested that these resources should be aligned with the organisation’s specific policies and practices. These suggestions were intended to support line managers, particularly those with limited experience or confidence in managing chronic conditions.

**Change made (Discussion, lines 1013-1015)**

Stakeholders emphasised the importance of better supporting line managers through education about chronic pain and upskilling those with limited management experience who may find conversations with employees challenging.

**Comment 69 (Discussion)**

Given the ‘gaps’ in organisational support, assisting people to self-manage chronic pain at work could benefit both the individual employees and improve organisational outcomes. However, a 2022 study of 107 organisations found that the majority did not provide any self-management materials or education for people with chronic pain *[14]. I completely follow your line of thinking, but can't see the link to the results?*

**Response**

Thank you for this comment. We have revised the Discussion to more clearly link this interpretation to our findings. Specifically, we now explicitly reference the gaps in organisational support identified across the themes, including variability in provision, inconsistent line manager support, and limited access to services such as occupational health.

**Change made (Discussion, lines 1023-1026 inclusive)**

In addition to additional resource for line managers, almost all participants in our stakeholder study felt that there was a need to better support employees to self-manage chronic pain. This appears to be compounded by gaps and variability in organisational support —including limited policies, inconsistent line manager support, and restricted access to services such as occupational health.

**Comment 70 (Discussion)**

Almost all participants in our stakeholder study felt that there was a need for additional tools. *can you elaborate (how have others done, what could be on the research agenda)?*

**Response**

We have elaborated this point in the Discussion by briefly situating our findings in relation to existing approaches to chronic pain self-management and outlining potential areas for future research, including the development and evaluation of workplace-focused resources.

**Change made (Discussion, lines 1027-1034 inclusive)**

Supporting self-management of chronic pain at work could benefit both employees and employers, however a 2022 study of 107 organisations found that the majority did not provide any self-management materials or education for people with chronic pain [14]. Furthermore, existing approaches to chronic pain self-management have typically focused on specific strategies (e.g., exercise or mindfulness) or particular conditions (e.g., low back pain), and existing digital interventions rarely address the challenges of managing chronic pain in the workplace. Stakeholders in our study emphasised the need for more comprehensive, workplace-relevant resources that incorporate a range of strategies and provide practical guidance for use in real-world work settings.

**Comment 71 (Discussion)**

However, The Pain-at-Work Toolkit feasibility trial [24] was commended for ‘shining a light’ on the impact of chronic pain in the workplace and highlighting the importance of prevention and early intervention before repeated or prolonged sickness absence. *Background?*

**Response**

We have clarified that this point reflects stakeholder accounts rather than background literature. The text has been revised to explicitly indicate that participants recognised and valued the contribution of the Pain-at-Work Toolkit feasibility trial.

**Change made (Discussion, lines 1036-1038 inclusive**

Reflecting these identified gaps in organisational resources for chronic pain, stakeholders in organisations that were randomised to the intervention arm of the trial were overwhelmingly positive towards the Pain-at-Work Toolkit[24] perceiving it as a comprehensive resource to support employees.

**Comment 72 (Discussion)**

The organisations that were randomised to the intervention arm of the trial were overwhelmingly positive towards the Pain-at-Work Toolkit [24], feeling it offered a comprehensive resource to enable employees to self-manage chronic pain at work. Stakeholders also reacted positively to the concept of producing resources specifically for line managers which would complement the existing employee-facing Pain-at-Work Toolkit. *outside the aim of this study?*

**Response**

Thank you for this comment. Organisational perceptions of the Pain-at-Work Toolkit are a key focus of this study (RQ3), and we have clarified this link in the manuscript. We agree that the development of resources specifically for line managers is beyond the primary scope of the study; however, this emerged inductively from stakeholder discussions and has been retained as an indicative finding. The text has been revised to reflect this more clearly.

**Change made (Discussion, lines 1039-1043 inclusive)**

In addition, stakeholder discussions highlighted a perceived gap in resources for line managers, suggesting a potential area for future development beyond the primary scope of this study. This represents a key target for implementation which we intend to address through the development of a targeted toolkit for line managers, with potential to improve both employee wellbeing and organisational outcomes.

**Comments 73, 74 and 75 (Discussion)**

Given the demographics of our sample, further research would be needed to understand the views of younger stakeholders (our stakeholders had a mean age of 48.6 years which might be expected for their level of seniority), male stakeholders (the majority in our study were female) and stakeholders from global ethnic majority backgrounds.) *? Is this a result? Results?*

**Response**

Thank you for this useful comment. We have now included participant demographic information (age, gender, and ethnicity) in the Results section under a new “Stakeholder demographics” subsection. These data are referenced in the Strengths and Limitations section to contextualise the sample and its implications for generalisability.

**Change made (Results, Table 3 stakeholder demographics, starts line 266)**

**Change made (Strengths and Limitations, lines 1060-1064 inclusive)**

The demographic profile of our sample (as reported in the Results, (mean age of 48.6 years and predominantly female) and minority representation from global ethnic majority backgrounds represents a limitation of the study. Further research is needed to capture the perspectives of younger stakeholders, male stakeholders, and those from more diverse ethnic backgrounds.

We thank you for the opportunity to revise and resubmit our manuscript and look forward to your response.

For your reference, the corresponding author is:

Professor Holly Blake, School of Health Sciences, Queen's Medical Centre, Nottingham, NG7 2HA.

Email:	holly.blake@nottingham.ac.uk
