# Supplementary material for: “Shining a light on chronic pain”: A qualitative study of stakeholder views towards chronic pain at work and the Pain-at-Work Toolkit
Source: PLoS One. 2026 Jul 2;21(7):e0351938. doi: 10.1371/journal.pone.0351938 (PMC13327183; doi:10.1371/journal.pone.0351938)
Supplement: Peer review history

# Peer review: Decision Letter 0

**DOI:** 10.1371/journal.pone.0351938.r001

**Authors:** Ilse Bloom

24 Mar 2026

PONE-D-25-48590

“Shining a light on chronic pain”: a qualitative study of stakeholder views towards chronic pain at work and the Pain-at-Work Toolkit

PLOS One

Dear Dr. Blake,

-->Thank you for submitting your manuscript to PLOS ONE. After careful consideration, we feel that it has merit but does not fully meet PLOS ONE’s publication criteria as it currently stands. Therefore, we invite you to submit a revised version of the manuscript that addresses the points raised during the review process.

Please note that we have only been able to secure a single reviewer to assess your manuscript. We are issuing a decision on your manuscript at this point to prevent further delays in the evaluation of your manuscript. Please be aware that the editor who handles your revised manuscript might find it necessary to invite additional reviewers to assess this work once the revised manuscript is submitted. However, we will aim to proceed on the basis of this single review if possible.

The comments from the reviewer can be found below and in the attached file. Please review these comments and make the appropriate revisions to your manuscript alongside a point-by-point response to the concerns raised.

Please submit your revised manuscript by May 07 2026 11:59PM. If you will need more time than this to complete your revisions, please reply to this message or contact the journal office at plosone@plos.org. When you’re ready to submit your revision, log on to https://www.editorialmanager.com/pone/ and select the 'Submissions Needing Revision' folder to locate your manuscript file.

Please include the following items when submitting your revised manuscript:

- A letter that responds to each point raised by the academic editor and reviewer(s). You should upload this letter as a separate file labeled 'Response to Reviewers'.
- A marked-up copy of your manuscript that highlights changes made to the original version. You should upload this as a separate file labeled 'Revised Manuscript with Track Changes'.
- An unmarked version of your revised paper without tracked changes. You should upload this as a separate file labeled 'Manuscript'.

If you would like to make changes to your financial disclosure, please include your updated statement in your cover letter. Guidelines for resubmitting your figure files are available below the reviewer comments at the end of this letter.

If applicable, we recommend that you deposit your laboratory protocols in protocols.io to enhance the reproducibility of your results. Protocols.io assigns your protocol its own identifier (DOI) so that it can be cited independently in the future. For instructions see: https://journals.plos.org/plosone/s/submission-guidelines#loc-laboratory-protocols. Additionally, PLOS ONE offers an option for publishing peer-reviewed Lab Protocol articles, which describe protocols hosted on protocols.io. Read more information on sharing protocols at https://plos.org/protocols?utm_medium=editorial-email&utm_source=authorletters&utm_campaign=protocols.

We look forward to receiving your revised manuscript.

Kind regards,

Ilse Bloom

Staff Editor

PLOS One

Journal Requirements:

When submitting your revision, we need you to address these additional requirements.

1. Please ensure that your manuscript meets PLOS ONE's style requirements, including those for file naming. The PLOS ONE style templates can be found at

https://journals.plos.org/plosone/s/file?id=wjVg/PLOSOne_formatting_sample_main_body.pdf and

https://journals.plos.org/plosone/s/file?id=ba62/PLOSOne_formatting_sample_title_authors_affiliations.pdf

2. Thank you for stating the following financial disclosure:

This project was funded by the Nuffield Foundation’s Oliver Bird Fund and Versus Arthritis (ref: OBF/FR-000023820), but the views expressed are those of the authors and not necessarily those of the funders.

Please state what role the funders took in the study. If the funders had no role, please state: "The funders had no role in study design, data collection and analysis, decision to publish, or preparation of the manuscript."

If this statement is not correct you must amend it as needed.

Please include this amended Role of Funder statement in your cover letter; we will change the online submission form on your behalf.

3. Thank you for stating the following in the Competing Interests/Financial Disclosure section:

HB and VA are authors of the Pain-at-Work Toolkit. HB is partially supported by the NIHR HealthTech Research Centre for Rehabilitation. KW-B reports personal fees from the Nuffield Foundation Oliver Bird Fund and Versus Arthritis to support their input to the study. DM reports grant support from Pfizer and Eli Lilly outside this study. DAW reports grant support from Pfizer, Eli Lilly, GlaxoSmithKline, UCB, and Orion Pharma outside of this study. All other authors declare no conflicts of interest.

We note that one or more of the authors are employed by commercial companies: Pfizer, Eli Lilly, GlaxoSmithKline, UCB, and Orion Pharma

a. Please provide an amended Funding Statement declaring this commercial affiliation, as well as a statement regarding the Role of Funders in your study. If the funding organization did not play a role in the study design, data collection and analysis, decision to publish, or preparation of the manuscript and only provided financial support in the form of authors' salaries and/or research materials, please review your statements relating to the author contributions, and ensure you have specifically and accurately indicated the role(s) that these authors had in your study. You can update author roles in the Author Contributions section of the online submission form.

Please also include the following statement within your amended Funding Statement.

“The funder provided support in the form of salaries for authors [insert relevant initials], but did not have any additional role in the study design, data collection and analysis, decision to publish, or preparation of the manuscript. The specific roles of these authors are articulated in the ‘author contributions’ section.”

If your commercial affiliation did play a role in your study, please state and explain this role within your updated Funding Statement.

b. Please also provide an updated Competing Interests Statement declaring this commercial affiliation along with any other relevant declarations relating to employment, consultancy, patents, products in development, or marketed products, etc.

Within your Competing Interests Statement, please confirm that this commercial affiliation does not alter your adherence to all PLOS ONE policies on sharing data and materials by including the following statement: "This does not alter our adherence to PLOS ONE policies on sharing data and materials.” (as detailed online in our guide for authors http://journals.plos.org/plosone/s/competing-interests) . If this adherence statement is not accurate and there are restrictions on sharing of data and/or materials, please state these. Please note that we cannot proceed with consideration of your article until this information has been declared.

Please include both an updated Funding Statement and Competing Interests Statement in your cover letter. We will change the online submission form on your behalf.

4. In the online submission form, you indicated that your data will be submitted to a repository upon acceptance. We strongly recommend all authors deposit their data before acceptance, as the process can be lengthy and hold up publication timelines. Please note that, though access restrictions are acceptable now, your entire minimal dataset will need to be made freely accessible if your manuscript is accepted for publication. This policy applies to all data except where public deposition would breach compliance with the protocol approved by your research ethics board. If you are unable to adhere to our open data policy, please kindly revise your statement to explain your reasoning and we will seek the editor's input on an exemption.

5. If the reviewer comments include a recommendation to cite specific previously published works, please review and evaluate these publications to determine whether they are relevant and should be cited. There is no requirement to cite these works unless the editor has indicated otherwise.

[Note: HTML markup is below. Please do not edit.]

Reviewers' comments:

Reviewer's Responses to Questions

**Comments to the Author**

1. Is the manuscript technically sound, and do the data support the conclusions?

The manuscript must describe a technically sound piece of scientific research with data that supports the conclusions. Experiments must have been conducted rigorously, with appropriate controls, replication, and sample sizes. The conclusions must be drawn appropriately based on the data presented.

Reviewer #1: Partly

**********

2. Has the statistical analysis been performed appropriately and rigorously?

Reviewer #1: N/A

**********

3. Have the authors made all data underlying the findings in their manuscript fully available?

The PLOS Data policy requires authors to make all data underlying the findings described in their manuscript fully available without restriction, with rare exception (please refer to the Data Availability Statement in the manuscript PDF file). The data should be provided as part of the manuscript or its supporting information, or deposited to a public repository. For example, in addition to summary statistics, the data points behind means, medians and variance measures should be available. If there are restrictions on publicly sharing data—e.g. participant privacy or use of data from a third party—those must be specified.

Reviewer #1: Yes

**********

4. Is the manuscript presented in an intelligible fashion and written in standard English?

PLOS ONE does not copyedit accepted manuscripts, so the language in submitted articles must be clear, correct, and unambiguous. Any typographical or grammatical errors should be corrected at revision, so please note any specific errors here.

Reviewer #1: Yes

**********

5. Review Comments to the Author

Please use the space provided to explain your answers to the questions above. You may also include additional comments for the author, including concerns about dual publication, research ethics, or publication ethics. (Please upload your review as an attachment if it exceeds 20,000 characters)

Reviewer #1: Thank you for the opportunity to review this manuscript entitled “Shining a light on chronic pain: a qualitative study of stakeholder views towards chronic pain at work and the Pain-at-Work Toolkit.”

Overall, this manuscript addresses a highly important and relevant topic. Supporting individuals with chronic pain in the workplace is both timely and necessary, and the study clearly draws on a rich and valuable qualitative dataset. The manuscript is generally well written and has the potential to make a meaningful contribution to the field. However, I have some concerns that should be addressed to improve the clarity, coherence, and methodological transparency of the paper.

First, while the manuscript is well written, I found it difficult to follow the overall logic of the results (themes and subthemes). There appears to be many “sub-sub-themes” (particularly within Theme 1), which at times, obscures the central messages. Relatedly, at least one sub-theme appears closely aligned with the interview guide questions (e.g., “What policies and practices are in place to support people to self-manage chronic or persistent pain?”), raising concerns about whether they represent fully developed interpretive themes rather than descriptive categories.

Another issue the authors may wish to consider is how participants’ perspectives are represented. Specifically, how do you decide whether they are speaking on behalf of their organisation versus expressing personal views? This distinction could be important for interpreting the findings and could be clearly described in the Methods section.

A visual overview (e.g., a figure) might help the reader better understand the analytical structure, the relationships between themes, and aspects of the methodology. For instance, it is somewhat misleading that the Pain-at-Work Toolkit is highlighted in the title but not clearly reflected in the stated aim of the study. In fact, it was difficult to understand how the toolkit was used, how it informed the analysis, and how it relates to the methods and findings. It is also not entirely clear whether this is primarily a study of organisational practices, stakeholder perspectives, or a combination of both.

As I found the Methods section somewhat difficult to follow, the Results section is consequently challenging as well. In several places, analytic claims are not consistently supported by illustrative data (e.g., participant quotations), which makes it harder to assess the credibility of the interpretations. A better balance between analytic narrative and supporting data excerpts would strengthen the manuscript. Consider using a clearer structuring of themes and subthemes with consistent use of illustrative quotations and summaries at the end of each subtheme.

While the dataset appears to be a genuine treasure trove of relevant insights, I am not convinced that the current thematic structure fully captures its potential. I would encourage the authors to revisit whether their themes most effectively address the core research question, and whether alternative thematic constructions might provide a clearer and more compelling account.

This is a valuable and promising study, but I believe major revisions are needed to improve the clarity of the analytical approach, the coherence of the results, and the alignment between aims, methods, and findings.

Kind regards,

Morten Hoegh

**********

6. PLOS authors have the option to publish the peer review history of their article (what does this mean?). If published, this will include your full peer review and any attached files.

If you choose “no”, your identity will remain anonymous but your review may still be made public.

**Do you want your identity to be public for this peer review?**  For information about this choice, including consent withdrawal, please see our Privacy Policy.

Reviewer #1: No

**********

[NOTE: If reviewer comments were submitted as an attachment file, they will be attached to this email and accessible via the submission site. Please log into your account, locate the manuscript record, and check for the action link "View Attachments". If this link does not appear, there are no attachment files.]

To ensure your figures meet our technical requirements, please review our figure guidelines: https://journals.plos.org/plosone/s/figures

You may also use PLOS’s free figure tool, NAAS, to help you prepare publication quality figures: https://journals.plos.org/plosone/s/figures#loc-tools-for-figure-preparation.

NAAS will assess whether your figures meet our technical requirements by comparing each figure against our figure specifications.

Attachment Submitted filename: Manuscript .docx

# Author comment: Author response to Decision Letter 1

**DOI:** 10.1371/journal.pone.0351938.r002

30 Apr 2026

27/04/2026

Dear Editors,

We would like to thank you and the reviewer for your careful evaluation of our manuscript and for the constructive and insightful comments provided. We have revised the manuscript in response to all points raised and believe these changes have strengthened the clarity and quality of the paper.

Below, we provide a point-by-point response to each comment. Reviewer comments are shown in italics, followed by our responses in regular text. All changes in the manuscript are highlighted/tracked. The line numbers specified refer to the clean version of the manuscript rather than the one with tracked changes in red.

Please note that we have added a Data Availability Statement after Keywords and before Introduction and that the Discussion has been substantially revised in line with reviewer comments.

Comment 1(Introduction):

While there is a clear need to better support people with chronic pain at work, a recent survey…Consider paraphrasing?

Response

Thank you for this suggestion. We have revised this sentence to improve clarity and reduce similarity with the original source.

Change made (Introduction, line,94)

“The revised sentence now reads: There is increasing recognition of the need to better support people with chronic pain in the workplace.

Comment 2 (Introduction)

However existing tools do not tend to focus specifically on workplace strategies or provide in depth resources. Can you elaborate? Are there some that do?

Response

We thank the reviewer for questioning this. To our knowledge the Pain-at-Work Toolkit is novel but we have clarified its positioning.

Change made (Introduction, lines 104-105 inclusive)

However existing tools digital tools rarely focus specifically on workplace self-management strategies and where work-related content is included it is typically limited and lacks in depth resources.

Comment 3 (Introduction)

The Pain-at-Work Toolkit was developed to address this gap. Is there a gap or is this "a tool" for this particular purpose?

Response

We agree that the Pain-at-Work Toolkit is a tool designed for this purpose; however, it was developed specifically in response to a gap in existing digital interventions, which rarely address workplace self-management of chronic pain. We have clarified this in the manuscript:

Change made (Introduction, lines 105-107 inclusive)

The Pain-at-Work Toolkit was developed to address this identified gap by providing a digital resource specifically designed to support workplace self-management of chronic pain.

Comment 4 (Introduction)

This digital toolkit was co-created with healthcare professionals, employers and people with chronic pain… Not line managers or managers? If so why not?

Response

We thank the reviewer for seeking clarification. The cited study (Blake et al., 2022) used the umbrella term “employers” to encompass a range of organisational roles and responsibilities. This included line managers, as well as middle managers/team leaders and senior managers, all of whom were represented in both the initial stakeholder consultation and the subsequent employer survey. We have clarified this interpretation in our response but retained the original wording in the manuscript for consistency with the cited study.

Comment 5 (Introduction)

…aiming to provide a comprehensive resource…did you aim at producing or test the feasibility of the Pain-at-Work Toolkit? Or both?

Response

We thank the reviewer for this comment. The statement referring to the toolkit as a “comprehensive resource” relates specifically to its development, rather than the feasibility study. The aim at this stage was to produce a resource to support individuals in self-managing chronic pain at work. The feasibility study, described separately in the manuscript, had a distinct aim: to assess the feasibility, acceptability, usability, and safety of evaluating the toolkit ahead of a definitive trial.

Comment 6 (Introduction)

An open-label, 2-arm multicentre pragmatic cluster-randomised feasibility trial of the Pain-at-Work Toolkit. This compared an intervention group with ‘treatment as usual’ controls in working adults with chronic pain in England [24]. This is ongoing and will be reported separately. Is this relevant to the current paper? I find it more distracting than relevant.

Response

We agree and have deleted accordingly.

Change made (Introduction)

Comment 7 (Introduction)

…feasibility trial of the Pain-at-Work Toolkit. reference to published study protocol or online repository?

Response

We agree and have cited the published study protocol.

Change made (Introduction, lines 107)

The Pain-at-Work Toolkit was developed to address this identified gap by providing a digital resource specifically designed to support workplace self-management of chronic pain[24].

Comment 8 (Introduction)

The study aimed to explore how individuals with chronic pain are supported in the workplace from the perspective of organisational stakeholders…This study?

Response

We thank the reviewer for highlighting this lack of clarity. We have removed this text to avoid ambiguity and to reduce repetition of the research objectives.

Change made (Introduction)

Comment 9 (Methods, Study design)

This was a qualitative interview study, with individual semi-structured interviews. Reporting aligns with the consolidated criteria for reporting qualitative research (COREQ) [25] (S1). Since the aim was to explore the individual stakeholder’s views rather than gain a global perspective [26]; semi-structured interviews were conducted. This approach allowed the discussion to be focused on key topics while also allowing the researcher the flexibility and autonomy to explore relevant topics if and when they were raised [27]. I feel like you are repeating yourself somewhat in this section.

Response

We thank the reviewer for this helpful observation. To reduce repetition, we have removed overlapping study details from the Introduction and streamlined the description of the qualitative approach in the Methods section.

Comment 10 (Methods, Recruitment and Interview Schedule)

All but two of the interviews were audio and video recorded. How did you store the data and were there any personal data collected that may interfere with GDPR-regulations?

Response

We thank the reviewer for this important point. The study involved the collection of qualitative interview data, which included personal data. All data were handled in accordance with the General Data Protection Regulation. Participants provided informed consent prior to participation, and all data were pseudonymised during analysis and anonymised for reporting. Data were stored securely in line with institutional data protection policies.

Change made (Methods, lines 139-141 inclusive)

Study forms were held securely in locked cabinets and access was limited to authorised personnel. All data were held on secure password protected servers with restricted access.

Comment 11 (Methods, Recruitment and Interview Schedule)

Interviews lasted between 30 and 58 minutes with a mean of 44 minutes. To me this is a result?

Response

We agree that this information is more appropriately reported as a result. We have therefore moved the description of interview duration to the Results section under stakeholder characteristics.

Change made (Results, lines 253-254 inclusive)

Comment 12 (Results, Stakeholder Characteristics)

The target population was individuals (stakeholders) employed in a role providing management or support for employees… Maybe the answer to my earlier question about participants? Consider aligning.

Response

We thank the reviewer for this enquiry. The earlier query relates to participants in the feasibility trial, whereas this section refers to a separate qualitative study involving organisational stakeholders, conducted for different purposes and with a distinct target population. We have more clearly differentiated the two studies where mentioned

Comment 13 (Results, Stakeholder Characteristics)

…eligible organisation participating in the feasibility trial of the Pain-at-Work Toolkit [24]. Eligible organisations were defined as organisations located in England, from any sector (public, private or third) with 10 or more employees…Why this number (10)?

Response

We thank the reviewer for this requesting clarification. The threshold of ≥10 employees was selected in the feasibility trial - small organizations were excluded because very small workforces are less likely to employ multiple employees with chronic pain, limiting recruitment feasibility. We have not added this explanation to the current manuscript as this detail will be published in the broader feasibility study paper.

Comment 14 (Research Questions/RQs)

RQ3: How do organisations perceive the Pain-at-Work Toolkit as a mechanism for supporting people with chronic pain at work? (how) were the participants informed about or instructed in the use of the toolkit?

Response

We thank the reviewer for this helpful question. We have now clarified how participants were introduced to the Pain-at-Work Toolkit in the section entitled Overview of Stakeholders’ Responses to the Pain-at-Work Toolkit. This information relates specifically to a subset of participants (n=8) from organisations in the intervention arm of the feasibility trial, who were asked to reflect on the implementation of the toolkit within their organisation. As this formed a relatively small and specific component of the study, it has been described in the Results section rather than in the main Methods section.

Change made (Overview of Stakeholders’ Responses to the Pain-at-Work Toolkit, lines 899-905 inclusive)

As part of the feasibility trial, stakeholders had previously been involved in promoting the toolkit to employees. To support reflection during the interviews, stakeholders were provided with access to the toolkit and were invited to briefly remind themselves of its content prior to discussing their experiences and perceptions.

Comment 15 (Research Questions)

Stakeholders were also asked to estimate the number of employees in the organisation and the percentage of workers with chronic pain in their organisation at recruitment into the trial and then at interview. Why? Is this relevant to the aim of the study? Or was there a secondary/tertiary aim?

Response

We thank the reviewer for this important question. These data were collected to provide contextual insight into organisational stakeholders’ perceptions of the prevalence of chronic pain within their workforce. While not a primary aim, this information helped to inform interpretation of the qualitative findings. Specifically, as reported in Theme 1 (“Not all disabilities are visible”), several stakeholders were unable to estimate the proportion of employees with chronic pain, while others provided only broad approximations. This finding reinforces the perception of chronic pain as an often unseen and poorly recognised condition in the workplace. To improve clarity, we have clarified the purpose of collecting these data in the manuscript.

Change made (Research Questions, lines 180-183 inclusive)

Stakeholders were also asked to estimate the number of employees in the organisation and the percentage of workers with chronic pain in their organisation at recruitment into the trial and then at interview. These data were collected to provide contextual insight into organisational stakeholders’ perceptions of the prevalence of chronic pain within their workforce.

Comments 16, 17, 18, 19, 20, 21, 22, 23, 24, 25 (Analysis)

Comment 16

The first step involved the researcher (AT) familiarising themselves with the data and since the same researcher (AT) interviewed stakeholders, checked transcripts, and collaboratively conducted analysis, they retained a close connection. Only one researcher? Normally two or more would be involved in this phase.

Comment 17

How did beliefs and experiences of AT influence this phase? Did you (can you) elaborate on "where you come from" and how your (the teams') beliefs and experiences impacts/synthesises with the analysis and results?

Comment 18

These generated preliminary themes which reflected patterns of shared meaning across interviews. along with those of AT, I assume?

Comment 19

These overarching themes were discussed and reviewed with two other researchers (HB and EW). did they not familiarise themselves with the raw data (transcripts)? Or did they compare the preliminary themes to their own experiences and beliefs?

Comment 20

These themes were then further defined… with what / which underlying focus? I find it both necessary and highly interesting to know the impact of the researchers in this process.

Comment 21

alone anchored by a unifying concept… according to what standards or ideals? Did you have a specific theory in mind?

Comment 22

Analysis was conducted manually in Microsoft Word rather than using analysis software like NVivo. Since you have not informed the reader about all your "not choices", I suggest you delete this.

Comment 23

This decision was influenced by the researcher’s personal preference [33] and a desire to ensure the context of the qualitative data was retained. Is there any particular reason why this is included? I - personally - believe that NVivo makes the process easier and more structured, but it's by no means the "right way" to do it. However, I would suggest that you discuss if/how this can have impacted your results in the limitations section. Particularly, in the light of only having one researcher doing the initial analysis.

Comment 24

Reflexive thematic analysis places the researcher at the heart of the process rather than considering their subjectivity as bias or a lack of validity [28] and hence it is important to articulate the role of the researchers in the team. thank you -) Maybe move this section up?

Comment 25

HB is a behavioural scientist with 30 years of experience in public health and applied health research. WJC and EW have a background in health psychology and a particular research interest in work and health, including chronic pain and work. AT has a background in psychology and over 35 years’ experience as an industry researcher specialising in healthcare. Please elaborate a bit about how you see the topic, e.g., what are your preconceptions about the role of the managers, and what are you motivation to look into this particular topic (workplace interventions as a mean to support people with pain to remain at work)? This is likely to inform and influence your themes.

Response to comments 16 to 25 inclusive

We thank the reviewer for these thoughtful and related comments regarding researcher involvement, analytic approach, reflexivity, and methodological transparency. We agree that transparency regarding analytic rigour is important, particularly given a single researcher led the analysis. In response, we have substantially revised the Analysis section to provide a more detailed account of the analytic process, including the role of reflexivity and the contribution of the wider research team in reviewing and refining codes and themes. Three researchers were involved and we have clarified this. While initial coding was conducted by one researcher (AT), developing interpretations were discussed and critically reviewed with other members of the research team (HB and EW), who contributed additional disciplinary perspectives and supported the refinement of themes. We have also expanded the reflexivity section to more explicitly describe how the researchers’ backgrounds, perspectives, and motivations informed the analytic process, in line with the principles of reflexive thematic analysis, where researcher subjectivity is understood as an integral component of knowledge production rather than a source of bias to be eliminated. We have removed the previous reference to software choice, as we consider this to reflect analytic approach rather than a determinant of methodological quality. While qualitative data analysis software such as NVivo can support data management and organisation, its use is not required for rigorous analysis, particularly within a reflexive thematic analysis framework. Given the expanded detail now provided on analytic proced

Attachment Submitted filename: Response to reviewers.docx

# Peer review: Decision Letter 1

**DOI:** 10.1371/journal.pone.0351938.r003

**Authors:** Heather Leggett

4 Jun 2026

“Shining a light on chronic pain”: a qualitative study of stakeholder views towards chronic pain at work and the Pain-at-Work Toolkit

PONE-D-25-48590R1

Dear Dr. Blake,

We’re pleased to inform you that your manuscript has been judged scientifically suitable for publication and will be formally accepted for publication once it meets all outstanding technical requirements.

Within one week, you’ll receive an e-mail detailing the required amendments. When these have been addressed, you’ll receive a formal acceptance letter and your manuscript will be scheduled for publication.

An invoice will be generated when your article is formally accepted. Please note, if your institution has a publishing partnership with PLOS and your article meets the relevant criteria, all or part of your publication costs will be covered. Please make sure your user information is up-to-date by logging into Editorial Manager at Editorial Manager® and clicking the ‘Update My Information' link at the top of the page. For questions related to billing, please contact billing support.

If your institution or institutions have a press office, please notify them about your upcoming paper to help maximize its impact. If they’ll be preparing press materials, please inform our press team as soon as possible -- no later than 48 hours after receiving the formal acceptance. Your manuscript will remain under strict press embargo until 2 pm Eastern Time on the date of publication. For more information, please contact onepress@plos.org.

Kind regards,

Heather Leggett

Academic Editor

PLOS One

Additional Editor Comments (optional):

Reviewers' comments:

Reviewer's Responses to Questions

**Comments to the Author**

1. If the authors have adequately addressed your comments raised in a previous round of review and you feel that this manuscript is now acceptable for publication, you may indicate that here to bypass the “Comments to the Author” section, enter your conflict of interest statement in the “Confidential to Editor” section, and submit your "Accept" recommendation.

Reviewer #1: All comments have been addressed

**********

2. Is the manuscript technically sound, and do the data support the conclusions?

The manuscript must describe a technically sound piece of scientific research with data that supports the conclusions. Experiments must have been conducted rigorously, with appropriate controls, replication, and sample sizes. The conclusions must be drawn appropriately based on the data presented.

Reviewer #1: Yes

**********

3. Has the statistical analysis been performed appropriately and rigorously?

Reviewer #1: Yes

**********

4. Have the authors made all data underlying the findings in their manuscript fully available?

The PLOS Data policy requires authors to make all data underlying the findings described in their manuscript fully available without restriction, with rare exception (please refer to the Data Availability Statement in the manuscript PDF file). The data should be provided as part of the manuscript or its supporting information, or deposited to a public repository. For example, in addition to summary statistics, the data points behind means, medians and variance measures should be available. If there are restrictions on publicly sharing data—e.g. participant privacy or use of data from a third party—those must be specified.

Reviewer #1: Yes

**********

5. Is the manuscript presented in an intelligible fashion and written in standard English?

PLOS ONE does not copyedit accepted manuscripts, so the language in submitted articles must be clear, correct, and unambiguous. Any typographical or grammatical errors should be corrected at revision, so please note any specific errors here.

Reviewer #1: Yes

**********

6. Review Comments to the Author

Please use the space provided to explain your answers to the questions above. You may also include additional comments for the author, including concerns about dual publication, research ethics, or publication ethics. (Please upload your review as an attachment if it exceeds 20,000 characters)

Reviewer #1: Thank you for your thorough and thoughtful revisions.

I have reviewed the revised manuscript and am satisfied with the changes made. The addition of the thematic analysis flowchart (Supplementary File 5) and the summary statements at the end of each subtheme have significantly enhanced the clarity and scannability of the results. Furthermore, the expansions in the methodology section (regarding GDPR and reflexivity) and the stronger alignment between the results and the discussion have elevated the overall quality of the paper.

I want to congratulate the authors on a relevant, and interesting project. This study sheds important light on a critical and often overlooked workplace issue, and your findings provide valuable insights that will undoubtedly benefit both future research and practical workplace interventions.

I have no further comments and am happy to recommend this manuscript for publication.

**********

7. PLOS authors have the option to publish the peer review history of their article (what does this mean?). If published, this will include your full peer review and any attached files.

If you choose “no”, your identity will remain anonymous but your review may still be made public.

**Do you want your identity to be public for this peer review?**  For information about this choice, including consent withdrawal, please see our Privacy Policy.

Reviewer #1: No

**********

# Editor report: Acceptance letter

**DOI:** 10.1371/journal.pone.0351938.r004

**Authors:** Heather Leggett

PONE-D-25-48590R1

PLOS One

Dear Dr. Blake,

I'm pleased to inform you that your manuscript has been deemed suitable for publication in PLOS One. Congratulations! Your manuscript is now being handed over to our production team.

At this stage, our production department will prepare your paper for publication. This includes ensuring the following:

* All references, tables, and figures are properly cited

* All relevant supporting information is included in the manuscript submission,

* There are no issues that prevent the paper from being properly typeset

You will receive further instructions from the production team, including instructions on how to review your proof when it is ready. Please keep in mind that we are working through a large volume of accepted articles, so please give us a few days to review your paper and let you know the next and final steps.

Lastly, if your institution or institutions have a press office, please let them know about your upcoming paper now to help maximize its impact. If they'll be preparing press materials, please inform our press team within the next 48 hours. Your manuscript will remain under strict press embargo until 2 pm Eastern Time on the date of publication. For more information, please contact onepress@plos.org.

You will receive an invoice from PLOS for your publication fee after your manuscript has reached the completed accept phase. If you receive an email requesting payment before acceptance or for any other service, this may be a phishing scheme. Learn how to identify phishing emails and protect your accounts at https://explore.plos.org/phishing.

If we can help with anything else, please email us at customercare@plos.org.

Thank you for submitting your work to PLOS ONE and supporting open access.

Kind regards,

PLOS ONE Editorial Office Staff

on behalf of

Dr. Heather Leggett

Academic Editor

PLOS One
